# Multiplex immunofluorescence and single‐cell transcriptomic profiling reveal the spatial cell interaction networks in the non‐small cell lung cancer microenvironment

**DOI:** 10.1002/ctm2.1155

**Published:** 2023-01-01

**Authors:** Haoxin Peng, Xiangrong Wu, Shaopeng Liu, Miao He, Chao Xie, Ran Zhong, Jun Liu, Chenshuo Tang, Caichen Li, Shan Xiong, Hongbo Zheng, Jianxing He, Xu Lu, Wenhua Liang

**Affiliations:** ^1^ Department of Thoracic Oncology and Surgery China State Key Laboratory of Respiratory Disease & National Clinical Research Center for Respiratory Disease the First Affiliated Hospital of Guangzhou Medical University Guangzhou China; ^2^ Department of Clinical Medicine Nanshan School Guangzhou Medical University Guangzhou China; ^3^ Department of Computer Science Guangdong Polytechnic Normal University Guangzhou China; ^4^ Department of Artificial Intelligence Research Pazhou Lab Guangzhou China; ^5^ Medical Department Genecast Biotechnology Co., Ltd Beijing China; ^6^ Department of Medical Oncology The First People's Hospital of Zhaoqing Zhaoqing China

**Keywords:** cell interaction networks, deep learning algorithm, multiplex immunofluorescence, single‐cell RNA sequencing, tumour microenvironment

## Abstract

**Background:**

Conventional immunohistochemistry technologies were limited by the inability to simultaneously detect multiple markers and the lack of identifying spatial relationships among cells, hindering understanding of the biological processes in cancer immunology.

**Methods:**

Tissue slices of primary tumours from 553 IA∼IIIB non‐small cell lung cancer (NSCLC) cases were stained by multiplex immunofluorescence (mIF) assay for 10 markers, including CD4, CD38, CD20, FOXP3, CD66b, CD8, CD68, PD‐L1, CD133 and CD163, evaluating the amounts of 26 phenotypes of cells in tumour nest and tumour stroma. StarDist depth learning model was utilised to determine the spatial location of cells based on mIF graphs. Single‐cell RNA sequencing (scRNA‐seq) on four primary NSCLC cases was conducted to investigate the putative cell interaction networks.

**Results:**

Spatial proximity among CD20+ B cells, CD4+ T cells and CD38+ T cells (*r* ^2^ = 0.41) was observed, whereas the distribution of regulatory T cells was associated with decreased infiltration levels of CD20+ B cells and CD38+ T cells (*r* ^2^ = −0.45). Univariate Cox analyses identified closer proximity between CD8+ T cells predicted longer disease‐free survival (DFS). In contrast, closer proximity between CD133+ cancer stem cells (CSCs), longer distances between CD4+ T cells and CD20+ B cells, CD4+ T cells and neutrophils, and CD20+ B cells and neutrophils were correlated with dismal DFS. Data from scRNA‐seq further showed that spatially adjacent N1‐like neutrophils could boost the proliferation and activation of T and B lymphocytes, whereas spatially neighbouring M2‐like macrophages showed negative effects. An immune‐related risk score (IRRS) system aggregating robust quantitative and spatial prognosticators showed that high‐IRRS patients had significantly worse DFS than low‐IRRS ones (HR 2.72, 95% CI 1.87–3.94, *p* < .001).

**Conclusions:**

We developed a framework to analyse the cell interaction networks in tumour microenvironment, revealing the spatial architecture and intricate interplays between immune and tumour cells.

## Graphical abstract

- Deep learning algorithm on multiplex immunofluorescence images identified cell spatial patterns with prognostic effects in the lung cancer microenvironment.
- Single‐cell RNA‐sequencing revealed the cell interaction networks suggested by spatial paradigm analyses.
- Proximity among CD4+ T cells, CD20+ B cells and N1‐like neutrophils, and spatial compartmentalisation between cancer stem cells and CD8+ T cells, were associated with significantly longer disease‐free survival.

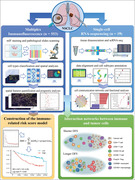

## 1 INTRODUCTION

The tumour microenvironment (TME) contains both cancer cells and multifarious lineages of stromal cells, including various assemblages of immune, vascular and mesenchymal cells, contributing to the de novo development of cancers.^1^, ^2^ Emerging evidence showed that, apart from the amounts and types of cells within the TME, the spatial configuration is crucial for modulating response to therapies and survival in various types of tumours, such as lung cancer (LC),^3^ breast cancer (BC)^4^ and gastric cancer.^5^, ^6^

Approaches to comprehending the intricate biology and the predictive and prognostic connotations of different cell populations in TME are mushrooming. Research focuses are transitioning from single‐color immunohistochemistry (IHC) imaging assays to multiplex IHC and multiplex immunofluorescence (mIF) techniques, enabling simultaneous detection of diverse protein markers in one tissue slice for the identification of multiple cell phenotypes.^7^, ^8^ Given that conventional IHC assays are confronted with several limitations like the inability to simultaneously detect multiple markers per tissue slice and the lack of identification of spatial relationships among cells, methods incorporating spatial organisation analyses are further developed. For instance, the metrics which quantify this spatial architecture, like nearest neighbour distance^9^ and mixing scores,^4^ have been proposed recently. Understanding the location of cells facilitates deeper insights into how immune cells interplay with tumour cells because both indirect and direct cell signal mechanisms require proximity between cells.^10^

LC is the most common cause of cancer death globally.^11^ Despite significant progress in new medicines and systemic therapies, like immune checkpoint inhibitors targeting TME, patients’ overall survival rate was still dismal.^12^ Consequently, there is an urgent need to improve the understanding of the TME of LC to better stratify patients for treatments and recognise novel targets for therapies to improve outcomes. The advances in spatial paradigm analyses, like deep convolutional neural networks, allow for a better description of cellular proximity in TME.^13^ Wang et al.^14^ developed a prognostic model of lung adenocarcinoma (LUAD) based on spatial features extracted by a deep learning algorithm on hematoxylin and eosin (HE)‐stained pathological graphs, improving the understanding of spatial locations of different cells and patient outcomes. However, they did not categorise the identified lymphocytes into different sub‐populations, hampering the interpretation of the interactions among different lineages of lymphocytes. A study by Enfield and colleagues^3^ reported that CD3+ CD8− T cells and CD8+ T cells surrounded by tumour cells were correlated with no recrudescence of LC, underscoring the cell sociology patterns play a crucial part in anti‐tumour immune response. Nonetheless, the sample size of their study was rather limited (*n* = 20), which may lack generalisation of the results.

Herein, we investigated the spatial immune‐tumour cell communication patterns and prognostic implications through stepwise procedures. First, the mIF test detecting 10 markers from 553 non‐small cell lung cancer (NSCLC) cases was conducted, evaluating the amounts of 26 phenotypes of cells in tumour stroma (TS) and tumour nest (TN). Second, the StarDist depth learning model was trained and developed using more than 2700 whole‐slide mIF images, generating the features to depict the TME from the identified phenotypes and spatial location of these cells.^15^ Eventually, to further understand the potential biological processes underlying the prognostic effects of different spatial patterns, we profile the transcriptomic features of the primary tumours and explore the putative cell interaction networks through receptor–ligand and downstream function analyses based on single‐cell RNA sequencing (scRNA‐seq). We found complex interactions among immune and tumour cells and revealed novel spatial parameters that were associated with the recurrence of LC. We further developed a prognostic tool, the immune‐related risk score (IRRS), to predict disease‐free survival (DFS) across different stages, resulting in stable and great performance.

## 2 METHODS

### 2.1 Study population and tissue collection

Formalin‐fixed, paraffin‐embedded (FFPE) tumour samples of NSCLC were retrospectively collected from 553 patients who underwent sub‐lobectomy/lobectomy with lymphadenectomy in the First Affiliated Hospital of Guangzhou Medical University (GZMU) between 2009 and 2011.

Patients inclusion criteria were: (a) staged IA to IIIB primary NSCLC based on the National Comprehensive Cancer Network (NCCN) criteria^16^, ^17^; (b) resected tissues were sufficient for mIF test; (c) pathological confirmation of all lymph nodes and resected tissues were provided; (d) without macroscopically or microscopically positive surgical margins. Patients exclusion criteria were: (a) patients with non‐invasive LC (e.g., minimally/ in situ invasive LUAD); (b) patients with preoperative neoadjuvant therapy; and (c) preoperative diagnostic biopsy.

The period from radical excision to local recurrence was defined as DFS. The ethics committee of First Affiliated Hospital of GZMU approved this study, and the study was carried out based on the Declaration of Helsinki.^18^ All patients provided informed consent for sample collection and mIF analysis, and all participants consented to the publication of research results.

### 2.2 Multiplex immunofluorescence test

The mIF staining was completed at Genecast Biotechnology Co., Ltd. (Beijing, China). Two panels, including a total of 10 markers, were detected, which were CD38, CD20, CD4, FOXP3, and CD66b in panel 1, and PD‐L1, CD163, CD8, CD68, and CD133 in panel 2. Multiple images were from serial sections from the same block per patient, and they were stained by DPAI and five markers in panel 1 or panel 2.

Detection of each panel was based on a 4‐μm thick slide cutting from FFPE NSCLC tissues. After deparaffinisation and rehydration, the slides were subjected to epitope retrieval through boiling for 20 min in Tris–EDTA buffer (pH = 9; Klinipath #643901, the Netherlands) at 97°C. Subsequently, endogenous peroxidase was blocked by incubation for 10 min in Antibody Block/Diluent (PerkinElmer #72424205, USA), and later the protein was blocked in 0.05% Tween solution at 26°C for 30 min. Then the five antigens in each panel were labelled by cyclic staining, including incubation of primary and secondary antibodies, tyramine signal amplification (TSA) visualisation and removing the TSA–antibody complex in Tris–EDTA buffer by microwave treatment for 20 min at 97°C. In each round, antibody labelling was followed after epitope retrieval and protein blocking as mentioned above. After cycle staining, each slide was counterstained with DAPI for 5 min and mounted in Pro‐Long Diamond Antifade Mountant (Thermo Fisher). As a positive control, fresh whole‐slide of normal human tonsils were included in every staining batch to evaluate the experiments’ reproducibility.

Furthermore, primary antibodies for CD133 and CD4 were incubated overnight at 4°C; CD20, CD38, PD‐L1, CD66b, CD8, CD68, CD163 and FOXP3 were incubated for 1 h at indoor temperature. Information of primary antibodies used was provided in Table S1. For secondary antibodies, we utilised anti‐rabbit/mouse horseradish peroxidase antibodies (Zsbio # PV‐6002 or PV‐8000) and incubated them for 10 min at 37°C. TSA visualisation was carried out through the Opal seven‐colour mIF Kit as we previously described.^19^

All slices were scanned utilising the PerkinElmer Vectra (Vectra 3.0.5). First, we used a 4× microscope objective lens to preview the whole picture of the slide, and then the 20× microscope objective lens was utilised to capture the details of the visual field. Finally, the images of each field were spliced to obtain the full view of the slide. The images were of high resolution (4028 × 3012 px), contributing to precise detection. Through the inform Advanced Image Analysis software (version 2.3.0), multi‐spectral images were unmixed with spectral libraries constructed from images with single stained tissue for each antigen.

First, 10–15 mIF images with high resolution in the image dataset were selected by random sampling (Supplementary Data 1). Second, our experienced pathologist (Dr. Bai Xuejuan) delineated the areas of TS and TN on the images to train the algorithm in the inForm software. Third, the inForm software was capable of detecting and segmenting the tissue slides into TS and TN according to their morphologies automatically. Segmentation of cells was also carried out in the inForm software (Figure S1). Determination of the suitable positive threshold X of each marker was implemented by two experienced pathologists (Dr. Bai Xuejuan and Dr. Wang Xin) independently, and disagreements were settled by consensus. Respectively, X, 2X and 3X was defined as the threshold of low (‘+’), median (‘++’) and high fluorescence strength (‘+++’). As illustrated by our previously article,^20^ the histochemistry score (H‐score) was conducted as:

$$\begin{array}{c} H-\mathrm{score}=\left(\mathrm{cells}\;\mathrm{with}\;\mathrm{low}\;\mathrm{fluorescence}\;\mathrm{strength}\right)\%\times 1\\ \;\;+\;\left(\mathrm{cells}\;\mathrm{with}\;\;\mathrm{median}\;\mathrm{fluorescence}\;\mathrm{strength}\right)\%\times 2\\ \;\;+\;\left(\mathrm{cells}\;\mathrm{with}\;\;\mathrm{high}\;\mathrm{fluorescence}\;\mathrm{strength}\right)\%\times 3 \end{array}$$

Markers for annotation of different cell were available in Table S2.^21^

### 2.3 Identification, segmentation and localisation of cells by deep learning model

**FIGURE 1:**
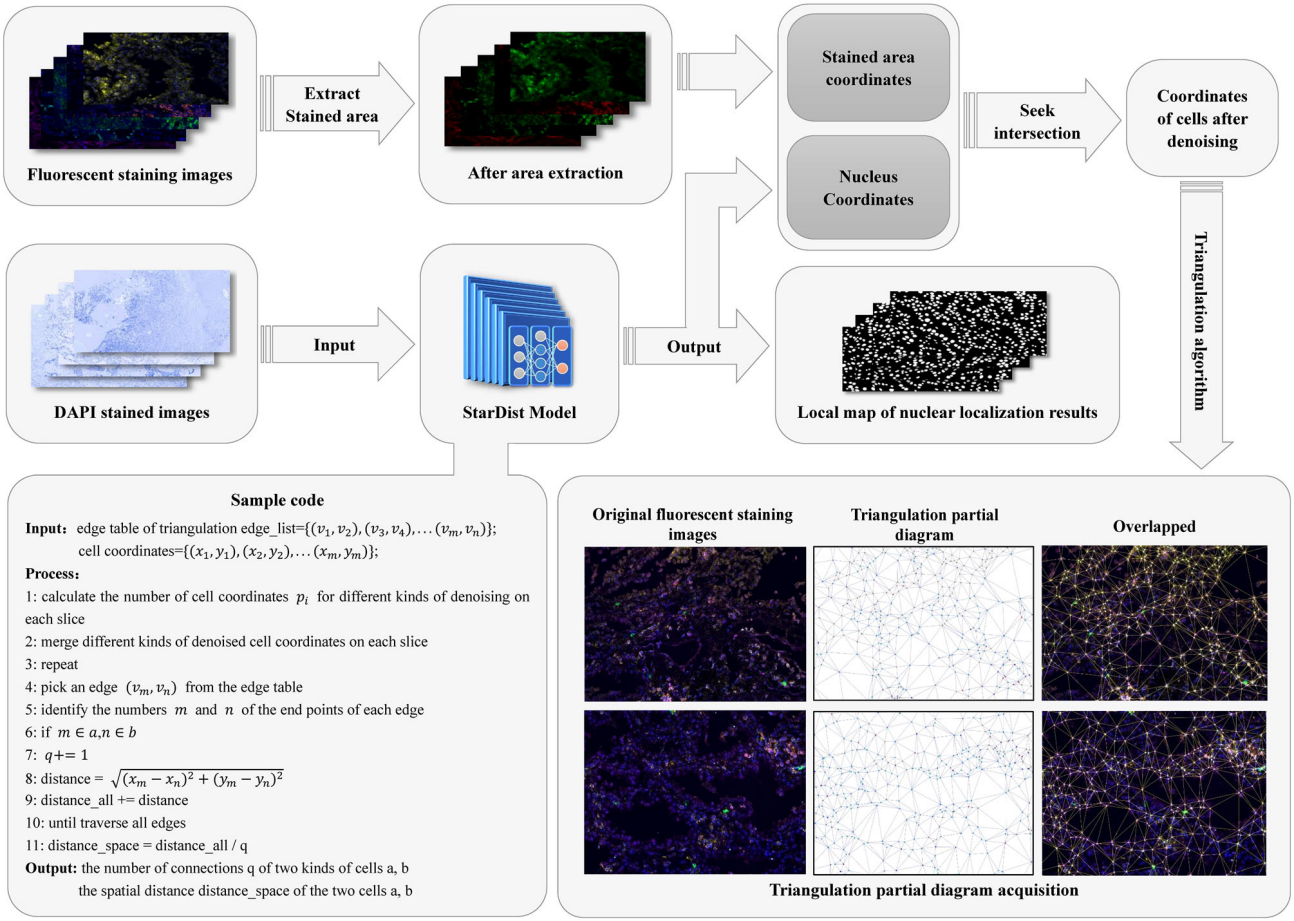
Deep learning pipeline to identify, segment and locate cells in the tumour microenvironment using multiplex immunofluorescence images.

The dataset included more than 2700 whole‐slide pathological images of 553 LC patients, and multiple images for one patient were from the serial sections from the same block. The deep learning pipeline to identify, segment and locate cells is illustrated in Figure 1.

We attempted to extract the fluorescent and get the cell coordinates after removing the noise because some non‐target cells may also be stained. Our fluorescent staining images contained R, G and B (i.e., red, green and blue, respectively) three channels. Given that the recognition performance was better in the R and G channels than in the B channel, then we separated the G and R channels of the fluorescent staining images, identifying six (CD4, CD20, CD38, CD66b, CD133 and CD163) and four (CD8, CD68, PD‐L1 and FOXP3) types of markers, respectively. Finally, we extracted the fluorescently stained areas for each image. For DAPI‐stained images, we used the StarDist depth training model to identify, segment and locate nuclei.^15^ The model predicted the polygon corresponding to each pixel, then parameterised the radial distance to obtain the target probability. Then U‐Net network was used to predict convex polygons, and finally, a final set of polygons was selected by non‐maxima suppression, each polygon representing a separate object instance. We inputted the DAPI‐stained image into the StarDist model to obtain the coordinates of the nucleus, that is, the cell coordinates, and then take the intersection of these cell coordinates and the coordinates of the fluorescent staining region extracted in the previous step to obtain the specific coordinates of each fluorescently stained cell after denoising.

We use the Delaunay triangulation algorithm to connect cells. The feature of this algorithm is to ensure that the nearest points form a triangle, the sum of the side lengths of the triangles is as small as possible, and each Delaunay triangle is circumscribed. The circle does not contain any other points on the surface. It also ensures that the minimum interior angle of the triangle in the Delaunay triangulation that the point set can form is as large as possible. And the triangle is as close to an equilateral triangle as possible. The connection diagram obtained by this connection method is reasonable and is more in line with the biological characteristics of cells in the complex TME.

### 2.4 Evaluation of segmentation performance

The ratio between the identified nuclei and the total ground truth nuclei was defined as detection coverage. Every ground truth nucleus matched the segmented nucleus, generating the maximum intersection over union (IoU). It is acknowledged that, at the object level, the higher the F1‐score is, the higher the model's segmentation quality. At the same time, the IoU is a crucial factor contributing to the F1‐score. Schmidt et al.^15^ have developed the Stardist model based on the pathological slides, and it achieved optimal cell segmentation performance with the threshold value of IoU set as 0.6. Given the large sample size (ours vs. Schmidt U's: 2700 images vs. 1000 images) and high resolution (ours vs. Schmidt U's: 4028 × 3012 px vs. 256 × 256 px) of our image dataset, it was time consuming and inefficient to label all the ground truth nuclei. Consequently, utilising the same Stardist model in the present study, the threshold of IoU was also set as 0.6. The nucleus was marked ‘matched’ if IoU for a ground truth nucleus > 0.6; otherwise, it was marked ‘unmatched’.

The pixel accuracy (PA) and F1‐score were used to assess the segmentation accuracy. Two experienced pathologists (Dr. Bai Xuejuan and Dr. Wang Xin) manually labelled the cells on 15 pathological images with random sampling and localised the nuclei by star‐convex polygons in the Stardist model. Combining the labels of cells provided by pathologists and the results predicted by the algorithm, we counted the values of true positive (TP), false positive (FP) and false negative (FN) (Figure S2). The accuracy and recall of cell nucleus segmentation were also calculated to generate the F1‐score based on the formula^22^:

$$F1\;=\;2\times \frac{\mathrm{precision}*\mathrm{recall}}{\mathrm{precision}+\mathrm{recall}}=2\times \left(\frac{\frac{\mathrm{TP}}{\mathrm{TP}+\mathrm{FP}}\times \frac{\mathrm{TP}}{\mathrm{TP}+\mathrm{FN}}}{\frac{\mathrm{TP}}{\mathrm{TP}+\mathrm{FP}}+\frac{\mathrm{TP}}{\mathrm{TP}+\mathrm{FN}}}\right).$$

### 2.5 Features extraction to describe the spatial relationships among cells

We extracted the number of connections and spatial distances between different types of cells. According to the length of each type of cell list, the subscript intervals of the coordinates of different types of cells in the general table could be obtained. The merged coordinates were inputted into the triangulation algorithm for connection, and the list of each edge in the output triangulation is called an edge table. Each edge in the edge table was a small list consisting of the numbers of the two endpoints that the edge corresponds to.

Here, we purposely matched the number of the input coordinates of the triangulation algorithm with the subscript of each element in the general table. Each edge in the table corresponded to the cell type and specific coordinates of the endpoint. On this basis, we established a loop to traverse all the edges on the triangulation, quickly determining the cell type and coordinates of the two endpoints of each edge and performing connection statistics and Euclidean distance calculations. After the loop was over, the number of connections between the two cells was output, and the spatial distance was obtained by dividing the total distance of each connection between the two cells by the number of connections.

Because 10 markers, including CD66b, CD4, CD38, FOXP3 and CD20 in panel 1, and CD163, PD‐L1, CD8, CD133 and CD68 in panel 2, were enrolled in the present work, the edges of the image were classified into 30 categories based on the formula of combination:

$$\left[C_{5}^{2}+5\;=\;\frac{5!}{2!\;\times \;\left(5-2\right)!}+5\right]\times 2\;=\;30$$

The number of connections (i.e., edges) for different classifications (yielding 30 features) and the average lengths of the connections (another 30 features) for each edge category were calculated for each graph patch. Eventually, 60 image features were extracted in total. The amounts of features were averaged for each patient if more than one pathology slide of one patient were available.

### 2.6 Quantitative and spatial features of cells in tumor microenvironment

Immune infiltration profiles, including the amounts and spatial distribution of cells, were used to decipher the TME characterisation. The percentage (%/sight) and density (per mm^2^) of cells in both TN and TS were measured by the mIF test, while the spatial variables, including the number of connections and the average length of connections between two types of cells (Figure S3), were summarised by mIF image‐derived deep learning model. Noteworthily, given that both indirect and direct cell signal mechanisms require proximity between cells, more connections and shorter length of connections between two types of cells indicate closer interplays between them. In contrast, fewer connections and longer lengths of connections suggest weakened interactions. We further calculated the relative amounts of spatial variables in each mIF slice for downstream analyses:

(a)

$$\begin{array}{ccc} & & \%\;\mathrm{average}\;\mathrm{length}\;\mathrm{of}\;A-B\;\mathrm{connections}=\mathrm{average}\;\mathrm{length}\\ & & \;\mathrm{of}\;A-B\;\mathrm{connections}/\left[\Sigma \left(n\right)i\times \left(\mathrm{length}\right)i/\Sigma n\right] \end{array}$$

in which *A* represents one type of cell while *B* represents another, (*n*)*i* equals the number of connections between a pair of cell types (e.g., *A* and *B*) and (length)*i* equals the length between them.

(b)

$$\begin{array}{ccc} & & \%\mathrm{number}\;\mathrm{of}\;A-B\;\;\mathrm{connections}=\mathrm{number}\;\mathrm{of}\\ & & \;A-B\;\mathrm{connections}/\Sigma \mathrm{number}\;\mathrm{of}\;Xi-Yi\;\mathrm{connections} \end{array}$$

in which *Xi* and *Yi* represent the *i*‐th of a pair of cell types.

A total of 66 cell amounts variables and 60 spatial variables were yielded ultimately. Through the Spearman rank correlation test, correlations between two variables were evaluated to explore the distribution patterns and possible communications.

### 2.7 Generation of the immune‐related risk score model

The entire cohort (*n* = 553) was split into a training cohort (*n* = 387, 70%) and a testing cohort (*n* = 166, 30%) using stratified sampling referring to the 10 events per variable (10 EPV) principle.^23^ Using the univariate and multi‐variate Cox regression analyses, markers significantly associated with DFS were identified. Second, a Least Absolute Shrinkage and Selection Operator (LASSO) Cox regression model was fitted to select the robust prognosticators among candidate factors with prognostic ability in univariate Cox results. Utilising the 10‐fold cross‐validations, we obtained the optimal lambda with the minimal partial likelihood deviance value.^24^ Finally, the proportion of the selected prognosticators and their regression coefficients of the univariate Cox regression were multiplied to construct the IRRS model. The IRRS for each patient was calculated as follows^25^:

$$\sum_{z\;=\;1}^{n}\ln\left(HR_{z}\right)\times \mathrm{proportio}n_{z}$$

wherein HR*_z_* is the HR for prognosticators, and proportion*_z_* represents the proportion for the *z*‐th variables. To evaluate the prognostic performance of the IRRS system, multi‐variate Cox regression analysis was applied. The assignment of high‐IRRS and low‐IRRS groups within the training cohort was executed following the optimal cut‐off value. In the testing and entire cohort, identical formulas and cut‐off value were utilised to validate the robustness of IRRS model.

### 2.8 Implementation of the single‐cell RNA sequencing

Four primary invasive adenocarcinoma samples from four patients after surgical resection (Table S3) were rapidly transported in ice‐cold H1640 (Gibco, Life Technologies) culture media. The tumour tissues were rinsed with phosphate‐buffered saline (PBS), cut into 1 mm^3^ cubic pieces, and digested with 0.25% trypsin. After being terminated by culture media supplemented with 10% foetal bovine serum, dispase (0.6 U/ml) and 10 ml of digestion medium with collagenase IV (100 U/ml) were added to the specimens. The digested samples were filtered by a 70‐μm nylon mesh, and filtered cells were collected through centrifuging for 5 min at 120×*g*, and 4°C. Cell pellets were re‐suspended with ice‐cold red blood cell lysis buffer and subjected to a 40‐μm nylon mesh. Following centrifugation, the cells were then collected with PBS, and the number of live cells were then calculated by an automatic cell counter. Cells were maintained on the ice during the process, and the entire process was completed in less than 1 h. Single‐cell suspensions were switched to barcoded scRNA‐seq libraries by the Gel Bead Kit, Single Cell 3′ Library, and Chromium Single Cell A Chip Kit (10X Genomics). To generate single‐cell gel beads in the emulsion (GEMs), the cell suspension was recorded into the Chromium single‐cell controller. The released RNA from lysed cells was barcoded by reverse transcription reaction in individual GEMs, and complementary DNA was then generated. The scRNA‐seq libraries were then established utilising the Single Cell 3′ Library Gel Bead Kit V3, and sequencing was done by an Illumina NovaSeq 6000 with a paired‐end 150–base pair (PE150) reading strategy (CapitalBio Technology, Beijing).

### 2.9 Processing single‐cell RNA sequencing data

Using human reference version GRCh38, raw expression matrices of each sample were created by the Cell Ranger (version 6.0.1) pipeline, and the Seurat package (version 4.0.3) was employed to analyse output‐filtered gene expression matrices.^26^ Once the following criteria were met, the cells were classified as low‐quality ones: (i) <200 or >5000 genes, (ii) <500 unique molecular identifiers (UMI) or (iii) >10% UMIs derived from the genome of mitochondria. After removing the low‐quality cells, the NormalizeData function was utilised for the normalisation of the gene expression matrices, and the FindVariableFeatures function was applied for calculating 2000 features with great intercellular variability. To reduce the dimensionality of the datasets after linear transformation with default parameters by the ScaleData function to generate scaled data, the RunPCA function was employed. Finally, all cells were clustered by the FindClusters and the FindNeighbors functions, and non‐linear dimensional reduction was conducted through the RunUMAP function. In brief, after the identification of 2000 features with great intercellular variability, the FindIntegrationAnchors function was used to generate the anchors between individual datasets. The IntegrateData function was then applied with the anchors to produce an expression matrix of cells with batch‐corrected from different datasets used for further analyses.

### 2.10 Annotating cell types and identifying cluster markers

Through a uniform manifold approximation and projection (UMAP) approach after nonlinear dimensionality reduction, cells from primary tumours were projected into the two‐dimensional field. Cells sharing similar characteristics could be clustered together subsequently. Widely accepted and classical markers of the corresponding cell types were utilised to annotate the clusters manually.^26^

### 2.11 Profiling interaction networks among different cell types

The receptor–ligand analysis based on the CellChat algorithm, which could evaluate the intercellular interaction networks from scRNA‐seq data, was implemented to assess the putative communications among different cell types.^27^ The Secreted Signaling Pathways and the Cell‐Cell Contact databases were selected for the main analyses. Two vital functions, including computeCommunProb and netVisual_bubble, were carried out to compare the probabilities of interactions mediated by ligand–receptor pairs from a certain cell group to another. To validate the observed communication networks, we obtained external processed scRNA‐seq data of LUAD (*n* = 13) in the GSE123904 cohort from the Gene Expression Omnibus (GEO) database (https://www.ncbi.nlm.nih.gov/geo/) and LUAS (*n* = 2) in the E‐MTAB‐6149 cohort from the ArrayExpress database (https://www.ebi.ac.uk/arrayexpress/) for the same analyses.^28^, ^29^

### 2.12 Gene set variation and enrichment analysis

To investigate the differences of function and allocate differential activities of pathways between different cell lineages, gene set variation analysis (GSVA) (R version 1.40.1) in hallmark gene sets and gene set enrichment analysis (GSEA) (version 1.44.0) in gene ontology sets (GO) were carried out.^30^, ^31^, ^32^ Pathways with a p_adj value less than 0.05 were considered significantly enriched.

### 2.13 Statistical analysis

Spearman rank correlation test was exploited to explore the associations between spatial and amounts variables. Comparisons of continuous variables between two or more groups were conducted by the T‐test and Kruskal–Wallis *H* test. To compare the differences in categorical data, the Chi‐square test and Fisher's exact test were employed. Prognostic effects of variables were assessed by univariate and multi‐variate Cox regression analysis controlling variables of sex, age, N stage, T stage, vascular cancer embolus and the number of lymph nodes dissection. Discrepancies of DFS between two groups were estimated by the log‐rank test and the Kaplan–Meier method. The receiver operating characteristic (ROC) and time‐dependent area under curve (AUC) curve were implemented for evaluating the classification performance in DFS of the IRRS model. The AUC between IRRS system and traditional TNM system was compared by DeLong's test. X‐tile software was utilised to choose the optimal cutoff value of IRRS. Plotting and statistical analyses were implemented in SPSS (version 25.0), R (version 4.0.4), GraphPad Prism (version 8.0) and X‐tile (version 3.6.1). Statistical tests were two‐sided, and *p* value < .05 was considered significant.

## 3 RESULTS

### 3.1 Demographic characteristics of included patients

A total of 553 patients with stage IA‐IIIB NSCLC that met inclusion criteria were eventually enrolled, among which 240 cases (43.4%) relapsed during the follow‐up time (Table 1). The median DFS was 1678 days, and the 1‐, 3‐ and 5‐year recurrence rates were 13.9, 36.9 and 60.4%, respectively. Male (58.8%), stage IB (30.2%) and LUAD (69.1%) patients constituted the majority of the cases. Patients with more advanced clinical (cTNM) stage (*p* < .001) and confirmed visceral pleural invasion (VPI) (*p* = .015) had a greater propensity for recurrence (Table 1).

**TABLE 1:** Clinicopathologic characteristics of the included patients

Characteristics	Number	%	Without occurrence (*n* = 313)	With occurrence (*n* = 240)	Mann–Whitney *U* test (*p* value)
Age (%)					
≤60	284	51.4	163 (52.1%)	121 (50.4%)	.251
>60	269	48.6	150 (47.9%)	119 (49.6%)	
Mean ± SD	60 ± 11				
Gender (%)					
Male	325	58.8	164 (52.4%)	161 (67.1%)	.001
Female	228	41.2	149 (47.6%)	79 (32.9%)	
Histology (%)					
Lung adenocarcinoma	382	69.1	219 (70.0%)	163 (67.9%)	.634
Squamous cell lung cancer	131	23.7	74 (23.6%)	57 (23.8%)	
Others	40	7.2	20 (6.4%)	20 (8.3%)	
Clinical stage (%)					
IA	127	23	90 (28.8%)	37 (15.4%)	<.001
IB	167	30.2	123 (39.3%)	44 (18.3%)	
IIA	89	16.1	44 (14.1%)	45 (18.8%)	
IIB	30	5.4	12 (3.8%)	18 (7.5%)	
IIIA	138	25	44 (14.1%)	94 (39.2%)	
IIIB	2	0.4	0	2 (0.8%)	
T stage (%)					
T1	239	43.2	146 (46.6%)	93 (38.8%)	.004
T2	186	33.6	113 (36.1%)	73 (30.4%)	
T3	85	15.4	39 (12.5%)	46 (19.2%)	
T4	43	7.8	15 (4.8%)	28 (11.7%)	
N stage (%)					<.001
N0	346	62.6	242 (77.3%)	104 (43.3%)	
N1	79	14.3	31 (9.9%)	48 (20.0%)	
N2	128	23.1	40 (12.8%)	88 (36.7%)	
VPE (%)					
Yes	259	46.8	178 (56.9%)	81 (33.8%)	<.001
No	294	53.2	135 (43.1%)	159 (66.2%)	
VPI (%)					.015
PL0	206	37.3	131 (41.9%)	75 (31.3%)	
PL1	311	56.2	163 (52.1%)	148 (61.7%)	
PL2	36	6.5	19 (6.1%)	17 (7.0%)	
Dissected LNs (%)					
0–14	167	30.2	231 (73.8%)	85 (35.4%)	.140
≥15	386	69.8	82 (26.2%)	155 (64.6%)	
Mean ± SD	20 ± 11				

VTE, vascular tumour emboli; VPI, visceral pleural invasion; LNs, lymph nodes.

### 3.2 Segmentation performance of the StarDist model

In the pixel‐level accuracy, the value of PA was 99.996%, and the F1‐score was 81.39% in the object‐level accuracy. In conclusion, using the StarDist model to identify, segment and locate the cells was effective and stable.

### 3.3 Deciphering immune landscape and interactions among cells

**FIGURE 2:**
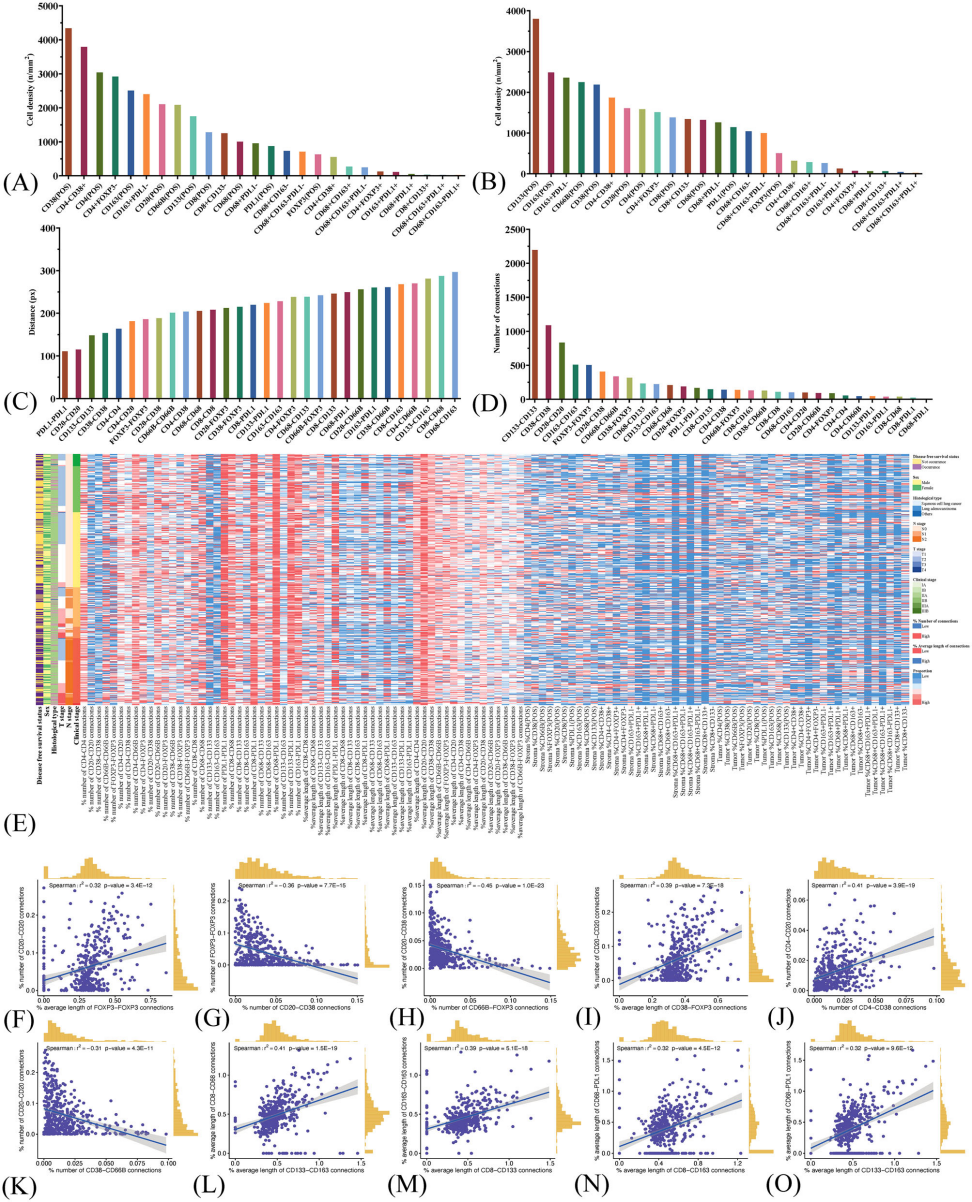
The cell composition and spatial relationships between different cells in the tumour microenvironment. Quantitative and spatial characteristics of different cells, including cell density in stroma (A) and tumour nest (B), the average length of connections (measured by pixel counts) (C) and the number of connections (D) between two markers. Clinical features, cell composition and spatial distribution of cells per patient on multiplex immunofluorescence images (E). The Scatter plots show close correlations between the spatial location of different cell types in the tumour microenvironment: average length of FOXP3–FOXP3 connections and number of CD20–CD20 connections (F); number of CD20–CD38 connections and number of FOXP3–FOXP3 connections (G); number of CD66b–FOXP3 connections and CD20–CD38 connections (H); average length of CD38–FOXP3 connections and number of CD20–CD20 connections (I); number of CD4–CD38 connections and number of CD4–CD20 connections (J); number of CD38–CD66b connections and number of CD20–CD20 connections (K); average length of CD133–CD163 connections and average length of CD8–CD68 connections (L); average length of CD8–CD133 connections and average length of CD163–CD163 connections (M); average length of CD8–CD163 connections and average length of CD68–PDL1 connections (N); average length of CD133–CD163 connections and average length of CD68–PDL1 connections (O). CD4, CD4+ T cell; CD38, CD38 + T cell; CD66b, CD66b+ neutrophil; FOXP3, FOXP3+ regulatory T cell; CD20, CD20+ B cell; CD8, CD8+ T cell; PD‐L1, PD‐L1+ cell; CD163, CD163+ macrophage; CD68, CD68+ macrophage; CD133, CD133+ cell.

It is acknowledged that cell–cell interaction is an integral part of the biological process of antitumour/protumour immunity, exemplified by the intricate network of communications between target cells and diverse immune cells. We first explored the correlations between different cell populations by their amounts. We found that CD38+ T cells and CD133+ cancer stem cells (CSCs) were the most abundant cell populations in TS and TN, respectively (Figures 2A and B). CD163+ macrophages (i.e., M2 macrophages) were enriched in TN while CD4+ T cells and CD20+ B cells were enriched in TS (both cell density > 2000/mm^2^). Neutrophils were abundant both in TN (2247/mm^2^) and TS (2087/mm^2^). Strong and significant associations between intrastromal Tregs and neutrophils (*r* ^2^ = 0.50), intrastromal CD20+ B cells and CD4+ T cells (*r* ^2^ = 0.45), intrastromal CD38+ T cells and CD20+ B cells (*r* ^2^ = 0.38), intratumoural CD8+ T cells and PD‐L1+ cells (*r* ^2^ = 0.44), intratumoural CD133+ CSCs and M1 macrophages (*r* ^2^ = 0.38) were observed (Figure S4), similar to our previous findings.^19^

With the development of spatial transcriptome study, it is anticipated that the spatial analysis can produce more useful and advanced information which cannot be answered by rough measures of cell amounts. Consequently, we then investigated the cell distribution patterns to better decipher the TME (Table S4). Closer proximity and more connections were observed in CD20+ B cells, CD38+ T cells and CD133+ CSCs. In comparison, longer distances and fewer connections were found in CD68+ macrophages and CD8+ T cells, and neutrophils and CD4+ T cells (Figures 2C–E).

It is well known that regulatory T cells (Tregs), marked by FOXP3, are the major contributors to forming immunosuppressive TME by releasing cytokines like transforming growth factor (TGF)‐β and interleukin (IL)‐4.^33^ Accordingly, we found modest and inverse associations between the distribution of FOXP3+ Tregs and CD20+ B cells. That is, the closer proximity between Tregs predicted fewer CD20‐CD20 connections (*r* ^2^ = 0.32) (Figure 2F). And fewer connections between CD20+ B cells indicated weaker interactions, which may lead to their dysfunction. The distribution of FOXP3+ Tregs was additionally correlated with decreased infiltration level of CD38+ T cells (a subset of CD4+ T cells) (*r* ^2^ = −0.36) (Figure 2G).^34^

Interestingly, proximity between CD66b+ neutrophils and FOXP3+ Tregs were associated decreased infiltrating levels of CD20+ B cells and CD38+ T cells (*r* ^2^ = −0.45) (Figure 2H). Given CD20+ B cells and CD38+ T cells are involved in activating cytotoxic T cells for killing tumour cells, such distributed pattern seemed to show that FOXP3+ Tregs and CD66b+ neutrophils populations may synergistically attenuate this process (average length of CD38‐FOXP3 connections and number of CD20–CD20 connections, *r* ^2^ = 0.39; number of CD38‐CD66b connections and number of CD20‐CD20 connections, *r* ^2^ = −0.31). Moreover, we found proximity among CD4+ T cells, CD20+ B cells and CD38+ T cells, and such spatial structure has been reported to aid the formation of tertiary lymphoid structure (TLS)^35^ (Figures 2I–K).

Tumour‐associated macrophages (TAMs), mainly M2 macrophages (marked by CD163), are pivotal players in shaping TME.^36^ It has been reported that CSCs can produce TGF‐β in the surrounding milieu, favouring the functional polarisation of protumour TAMs.^37^ And in turn, TAMs facilitate protecting CSCs from the assaults of immune cells through expressing higher levels of PD‐L1 and releasing cytokines like IL‐10 and TGF‐β.^38^ Accordingly, we found close and positive associations between the distribution of macrophages, CD133+ CSCs and PD‐L1+ cells (average length of CD133‐CD163 connections and average length of CD68‐PDL1, *r* ^2^ = 0.32) (Figure 2N). Additionally, we found proximity among macrophages, CD133+ CSC and CD8+ T cells (average length of CD8–CD133 and average length of CD163–CD163, *r* ^2^ = 0.39; average length of CD133–CD163 connections and average length of CD8–CD68 connections, *r* ^2^ = 0.41; average length of CD8–CD163 and average length of CD68–PDL1, *r* ^2^ = 0.32) (Figures 2L–O), and such spatial pattern has been shown to prime T cells for exhaustion, possibly by the long‐term synaptic interactions between them.^39^ Ultimately, consistency analysis showed that higher infiltration levels of CD8+ T cells or CD133+ CSCs were associated with closer proximity between them (Figures S5A–E). And such spatially distributed patterns were supported by previous studies that CSCs tend to distribute closely to form the ‘niches’, which facilitate protection against the assault of the immune system and reserve their potential to metastasize.^40^

### 3.4 Differences in cell infiltration and spatial location between lung adenocarcinoma and squamous carcinoma

LUAD showed significantly higher infiltrating levels of CD4+ T cells in TS, while lung squamous cell carcinoma (LUSC) was characterised by higher infiltrating levels of PD‐L1+ cells, CD133+ CSCs and macrophages both in TS and TN, suggesting immune suppressive milieu (Figure S6). Closer proximity and stronger interaction between CD8+ T cells were observed in LUAD, whereas LUSC featured spatial proximity among PD‐L1+ cells, CD133+ CSCs and macrophages. In conclusion, T cells, CSCs and macrophages were crucial contributors to the heterogeneity in TME between LUAD and LUSC, aligning with the recent findings by Wang et al.^41^

### 3.5 Differences of cell spatial distribution patterns between patients with or without recurrence

**FIGURE 3:**
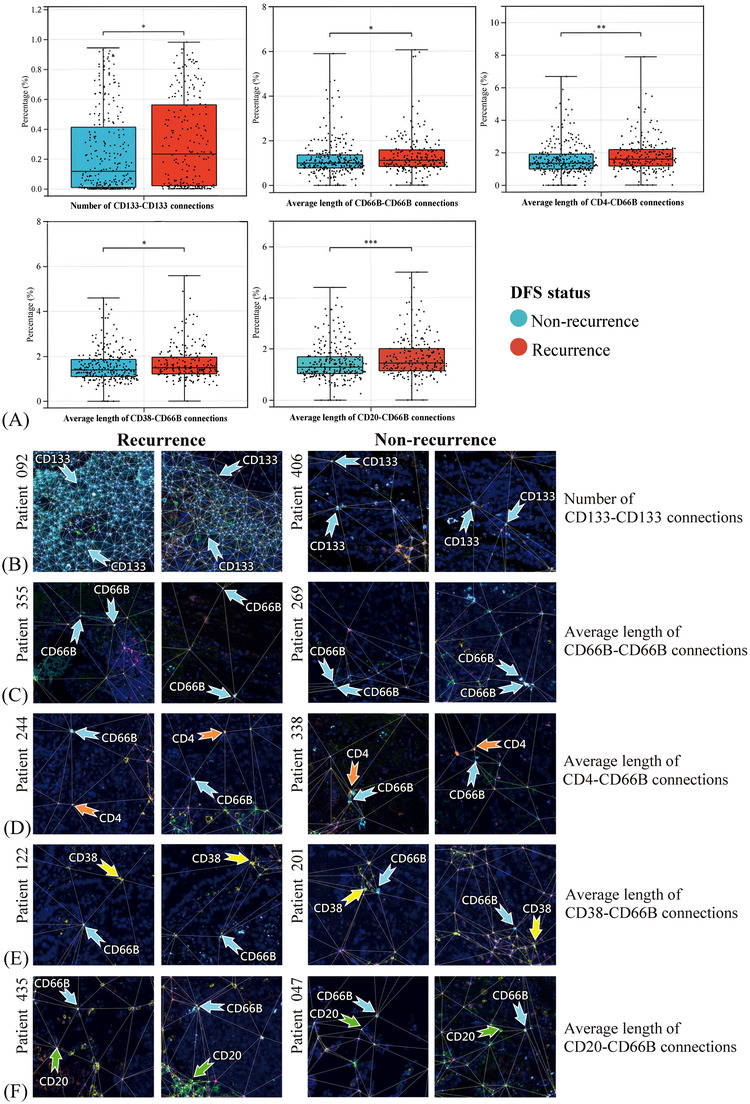
Comparison of spatial cell location in non‐small cell lung cancer (NSCLC) cases with or without recurrence. Discrepancies of the spatial distribution of cells between recurrent and non‐recurrent patients as evaluated by the Kruskal–Wallis *H* test (A). Representative multiplex immunofluorescence images exhibited the spatial configuration of cells within two fields from one tissue slide. More number of CD133–CD133 connections (B), longer distances of CD66b–CD66b connections (C), longer distances of CD4–CD66b connections (D), longer distances of CD38–CD66b connections (E) and longer distances of CD20–CD66b connections (F) were significantly associated with NSCLC recurrence. *p < .05; **p < .01; ***p < .001.

**FIGURE 4:**
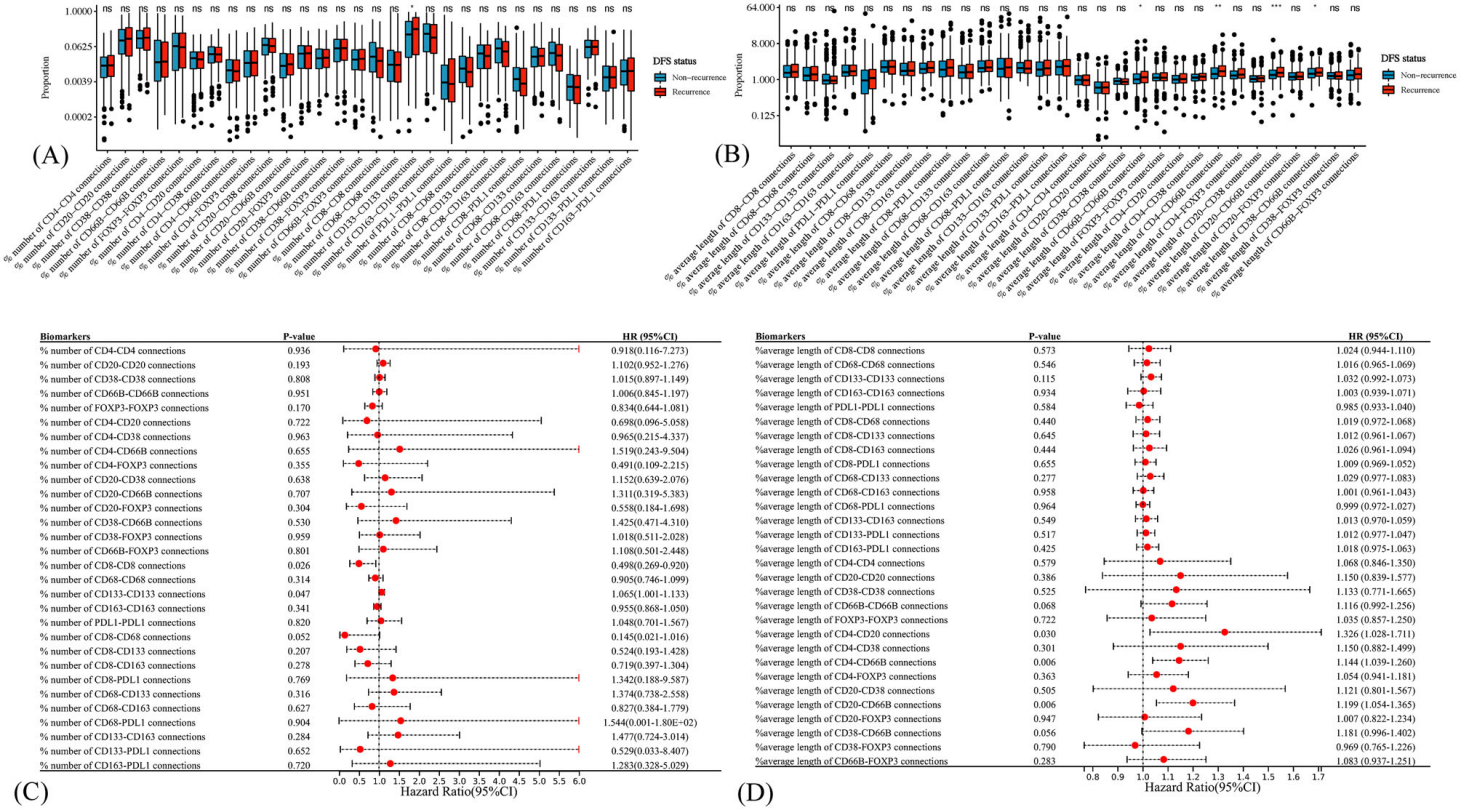
Prognostic effects of cell spatial variables. Cell location differences between patients with or without recurrence as evaluated by the Kruskal–Wallis *H* test (A and B). The prognostic effects of spatial features, including the number of connections (C) and the average length of connections (D), in disease‐free survival (DFS) as evaluated by univariate Cox analysis. *p < .05; **p < .01; ***p < .001; ns, non‐significant.

The proximity between CD133+ CSCs was observed in patients with tumour relapse than without (Figures 3A and B). Concurrently, longer distances between CD66b+ neutrophils (Figure 3C), CD66b+ neutrophils and CD4+ T cells (Figure 3D), CD66b+ neutrophils and CD38+ T cells (Figure 3E) and CD66b+ neutrophils and CD20+ B cells (Figure 3F) were observed in patients with recurrence than without, suggesting the putative vital effects of neutrophils on spatially adjacent T and B lymphocytes. Apart from these cell types, different spatial landscapes in patients with or without relapse were observed (Figures 4A and B).

### 3.6 Associations between spatial features and clinical characteristics

The distance between CD20+ B cells and CD66b+ neutrophils gradually increased with the advancing T stage (Figures S7A–F). Although null prognostic significance was found by multi‐variate Cox regression analysis, the distance between CD38+ T cells and CD4+T cells increased along with the more advanced cTNM and N stage (Figure S7D).

### 3.7 Prognostic effects of spatial variables

Univariate Cox results showed that closer proximity between CD8+ T cells predicted longer DFS (HR 0.498, 95%CI 0.269–0.920, *p* = .026). In contrast, closer proximity between CD133+ CSCs (HR 1.065, 95%CI 1.001–1.133, *p* = .047), longer distances between CD4+ T cells and CD20+ B cells (HR 1.326, 95%CI 1.028–1.711, *p* = .030), CD4+ T cells and CD66b+ neutrophils (HR 1.144, 95%CI 1.039–1.260, *p* = .006), and CD20+ B cells and CD66b+ neutrophils (HR 1.199, 95%CI 1.054–1.365, *p* = .006) were correlated with dismal DFS (Figures 4C and D). Multi‐variate Cox analyses further revealed proximity between CD8+ T cells (HR 0.515, 95%CI 0.283–0.937, *p* = .030), proximity between CD133+ CSCs (HR 1.079, 95%CI 1.014–1.148, *p* = .017) and longer distance between CD20+ B cells and CD66b+ neutrophils (HR 1.206, 95%CI 1.026–1.417, *p* = .023) were independent prognosticators in DFS (Figures S8A and B).

### 3.8 Construction and validation of the immune‐related risk score model

**FIGURE 5:**
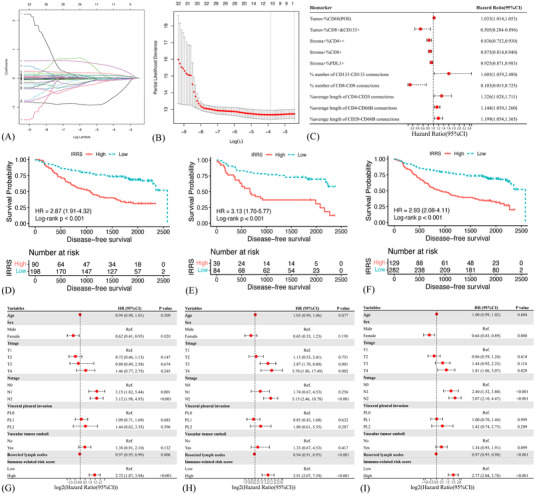
Construction of the immune‐related risk score (IRRS) model. Least absolute shrinkage and selection operator (LASSO) coefficient profiles of 35 spatial and quantitative variables in the 10‐fold cross‐validation (A). Partial likelihood deviance demonstrated by the LASSO regression model (B). Ten variables robustly associated with disease‐free survival (DFS) were selected by the LASSO model (C). Kaplan–Meier curves of DFS based on different IRRS groups in the training (D), testing (E) and entire (F) cohort. Forest plot of the multi‐variate Cox regression model demonstrated the prognostic significance of the IRRS model and clinical characteristics on DFS in the training (G), testing (H) and entire (I) cohort.

A total of 35 variables, including 30 cell amounts variables (Figure S8C) and five spatial variables, which were strongly predictive of DFS in the univariate Cox analyses, were incorporated into the LASSO regression model and ten‐fold cross‐validation to generate the best model. Eventually, the IRRS model incorporating five amounts variables and five spatial variables achieved the optimal efficiency to speculate the DFS (Figures 5A and B).

The identified robust prognosticators could be crudely classified into several categories: antigen processing and presentation (intratumoural CD68‐positive macrophages, average length of CD4–CD20 connections, average length of CD20‐CD66b connections), helper T cells (intratumoural CD4++ T cells and average length of CD4–CD66b connections), cytotoxic T cells (intratumoural CD8+CD133+ T cells, intrastromal CD8+ T cells and number of CD8–CD8 connections) and immune escape (intrastromal PD‐L1+ cells and number of CD133–CD133 connections) (Figure 5C).

The IRRS was calculated for each patient according to the linear combination of these variables weighted by their coefficients of univariate Cox analyses (Table S5). We further split the cases into a high‐IRRS subset and a low‐IRRS subset. Low‐IRRS patients showed significantly better DFS compared with those of high‐IRRS (log‐rank *p* < .001) (Figures 5D–F and Table S6). Such correlation remained significant in the multi‐variate Cox results in the training (HR 2.72, 95%CI 1.87–3.94, *p* < .001), testing (HR 3.91, 95%CI 2.07–7.39, *p* < .001) and entire (HR 2.77, 95%CI 2.04–3.78, *p* < .001) cohort (Figures 5G–I). Additionally, female and more lymph nodes resection implied longer DFS, whereas the advanced N stage indicated the worse in the training cohort (Figure 5G).

**FIGURE 6:**
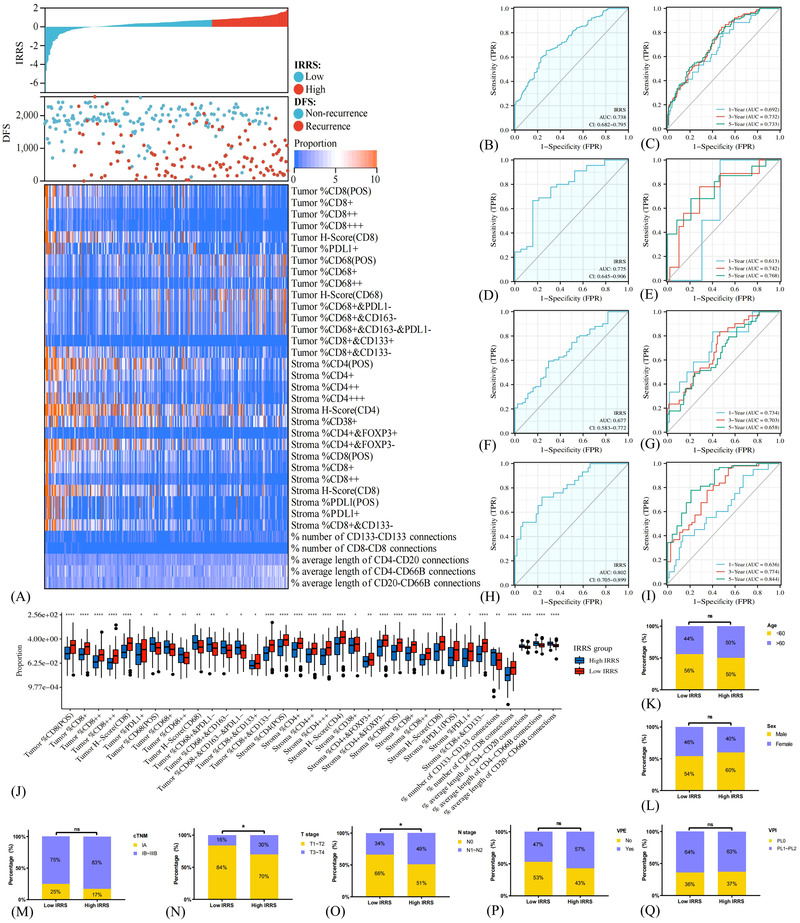
The immune landscape between different immune‐related risk score (IRRS) groups and the recurrence predictive performance of IRRS model in the training cohort. The distribution of IRRS, recurrence status and immune profiles of patients (A). Receiver operating characteristic (ROC) curves and area under curve (AUC) values of IRRS model for prediction of recurrence risk at 1, 3 and 5 years (B and C). The predictive performance of the IRRS model at 1, 3 and 5 years of stage I (E and F), stage II (G and H) and stage III (I and J) non‐small cell lung cancer. The infiltration disparities between high and low IRRS subgroups as evaluated by the Kruskal–Wallis *H* test (K). The disparities in the clinical characteristics between high and low IRRS subgroups as evaluated by the Chi‐square test (L–R). *p < .05; **p < .01; ***p < .001; ****p < .0001; ns, non‐significant.

To recognise the immune features responsible for LC recurrence, a heatmap of the 35 candidate variables’ immune landscapes and the scatterplot of DFS with corresponding IRRS were plotted (Figure 6A). We found that helper and cytotoxic T cells, such as CD4+ T cells, CD38+ T cells, CD8+ T cells and CD8+CD133+ T cells, were markedly enriched in the low‐IRRS subset, while high‐IRRS group showed a higher infiltrating degree of macrophages, such as CD68+ macrophages and CD68+CD163‐PDL1‐ macrophages (both *p* < .05) (Figure 6J). Significantly more CD8–CD8 connections were detected in the low‐IRRS subset, whereas the high‐IRRS subset showed more CD133–CD133 connections and longer distances of CD4–CD20, CD4–CD66b and CD20–CD66b connections (both *p* < .01) (Figure 6J). Moreover, high‐IRRS patients were in more advanced T stage and N stage compared with low‐IRRS populations (both *p* < .05) (Figures 6K–Q). Analyses in the testing cohort and entire cohort further validated these findings (Figure S9–10).

With sensitivity and specificity of 0.655 and 0.724, the IRRS model possessed relatively high predictive accuracy of DFS (AUC 0.738, 95%CI 0.682–0.795) (Figure 6B). The AUCs for predicting relapse at 1, 3 and 5 years were 0.692, 0.732 and 0.733, respectively (Figure 6C). The predictive performance of the IRRS model modestly improved when restricted to a specific cTNM stage (Figures 6D–I). The testing cohort (AUC 0.740, 0.707, 0.751 and 0.698 for overall, 1‐, 3‐ and 5‐year, respectively) and entire cohort (AUC 0.738, 0.696, 0.738 and 0.722 for overall, 1‐, 3‐ and 5‐year, respectively) showed comparable results (Figures S9 and S10). Despite without statistical significance, the IRRS model manifested better predictive ability than the cTNM system (0.738 vs. 0.686 in the training cohort; 0.740 vs. 0.624 in the testing cohort; 0.738 vs. 0.667 in the entire cohort) (Figure S11). Taken together, the IRRS signature presented stable and great performance in separating the recurrence and recurrence‐free LC patients after radical resection.

### 3.9 Single‐cell transcriptomic landscape of primary tumours

**FIGURE 7:**
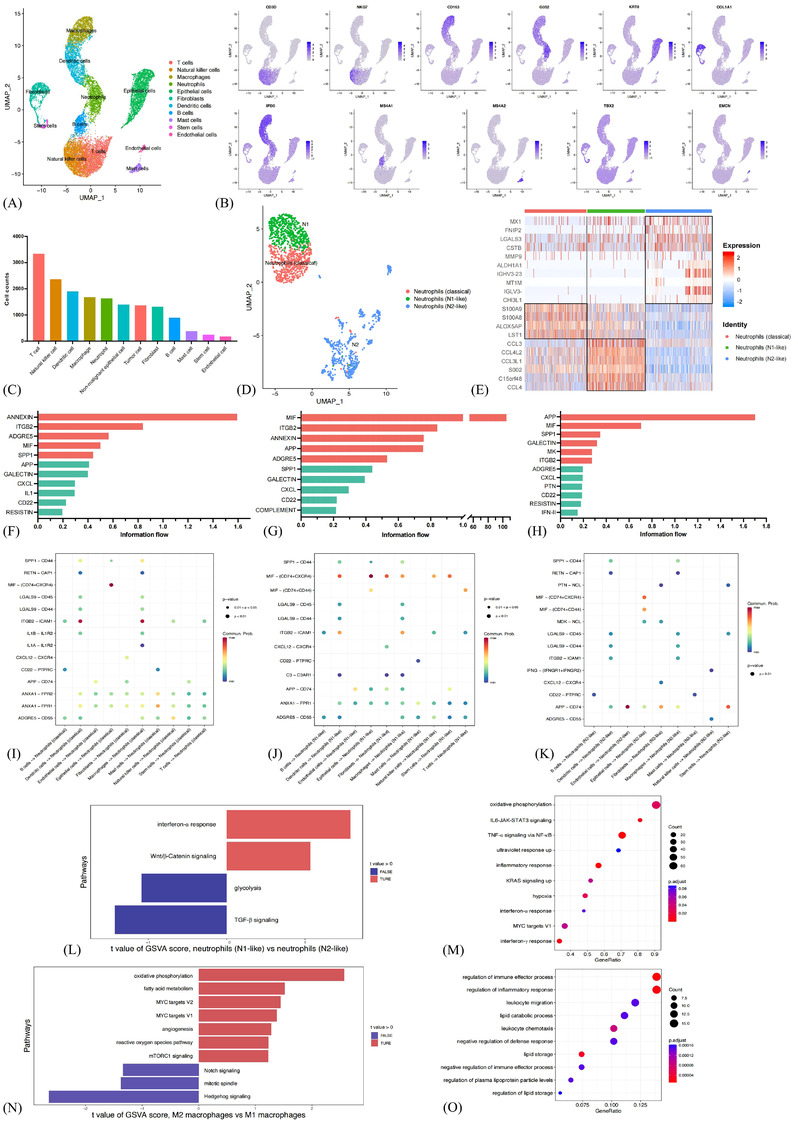
Single‐cell RNA sequencing profiling reveals cell composition and cell interaction networks in the lung cancer microenvironment. Eleven major cell lineages as represented in the UMAP plot (A). Canonical gene markers to label clusters in the UMAP plot, including CD3D for T cells, NKG7 for natural killer cells, CD163 for macrophages, G0S2 for neutrophils, KRTS for epithelial cells, COL1A1 for fibroblasts, IFI30 for dendritic cells, MS4A1 for B cells, MS4A2 for mast cells, TBX2 for stem cells and EMCN for endothelial cells (B). Contents of major cell types in the primary tumours (C). Classical, N1‐like and N2‐like neutrophils as represented in the UMAP plot (D). Heatmap of functional gene sets in neutrophil clusters (E). The overall information flow of each signal pathway with classical (F), N1‐like (G) and N2‐like (H) neutrophils as signal receivers. Communication probabilities mediated by ligand–receptor pairs in classical (I), N1‐like (J) and N2‐like (K) neutrophils. Bar plots indicating the altered hallmark pathways scored by gene set variation analysis (GSVA) in N1‐like than N2‐like neutrophils (L). Dot plots demonstrating enrichment of genes differentially expressed by N1‐like than N2‐like neutrophils through gene ontology (GO) analysis (M). Bar plots indicating the altered hallmark pathways scored by GSVA in M2‐like than M1‐like macrophages (N). Dot plots demonstrating enrichment of genes differentially expressed by M2‐like than M1‐like macrophages through GO analysis (O).

With quality filtering of scRNA‐seq data on 4 primary tumour samples, around 33.8 million unique transcripts were obtained from 16,459 cells (Figure S12). After correcting for reading depth, cells were merged into a dataset with adjustments for batch effects. Dimensionality reduction by UMAP and principal components analysis methods were subsequently conducted. Eleven major cell lineages, including T cells, epithelial cells, natural killer (NK) cells, dendritic cells, macrophages, neutrophils, fibroblasts, B cells, mast cells, stem cells and endothelial cells, were detected based on the expression of classical gene markers (Figures 7A and B and Table S7). T cells were the most abundant cell types in the primary tumours, followed by epithelial cells and NK cells. Myeloid cells, including dendritic cells, macrophages and neutrophils, were also enriched (Figure 7C). We further distinguished between tumour cells and non‐malignant epithelial cells using several marker genes, including MMP7, MMP9, MMP13 and HOXB2 (Figure S13). A total of 805 tumour cells and 563 non‐malignant epithelial cells were identified eventually.

### 3.10 Spatially adjacent N1‐like neutrophils could boost the proliferation and activation of T and B lymphocytes

To further understand the potential biological processes underlying the prognostic effects of neutrophils on spatially adjacent T and B lymphocytes, we first investigated the transcriptomic features of the neutrophil population and three subsets were identified (Figures 7D and E). The classical subtype was characterised by major expression of canonical neutrophil markers like S100A8 and S100A9. The N1‐like sub‐cluster displayed features of high expression levels of chemokines and cytokines (e.g., CCL3, CCL4 and C15orf48) and antigen presentation (e.g., HLA–DMB and CD74). The N2‐like sub‐population was characterised by immunosuppressive (e.g., TGFBI and LAGLS3) and pro‐angiogenic (e.g., MMP9) features.^42^, ^43^, ^44^

Then the CellChat algorithm was used to investigate the intercellular interaction networks between different clusters of neutrophils and other cell types. First, the overall information flow of each signal pathway was evaluated. The leading intercellular ligand–receptor pairs with neutrophils as signal receivers were distinct among classical (ANNEXIN, ITGB2, ADGRE5) (Figure 7F), N1‐like (MIF, ITGB2, ANNEXIN) (Figure 7G) and N2‐like (APP, MIF, SPP1) clusters (Figure 7H). N1‐like cluster showed increased stimulating interactions like MIF‐(CD74+CD44), ITGB2–ICAM1 and ADGRE5–CD55, which can promote proliferation and activation of T and B lymphocytes (Figure 7I). Stimulating interactions with chemotaxis and development functions on dendritic cells and NK cells, such as MIF‐(CD74+CD44)/(CD74+CXCR4), also increased in the N1‐like cluster than N2‐like cluster. However, inhibiting interactions like CD22–PTPRC and LAGLS9–CD44/CD45 on T and B lymphocytes increased in N2‐like than N1‐like cluster (Figure 7J). Classical neutrophils showed similar interaction networks to N1‐like neutrophils (Figure 7K).

In the external scRNA‐seq cohort of LUAD, eleven kinds of cell lineages were identified (Figure S14A and B and Table S8). Three subsets, including classical, N1‐like, and N2‐like, of neutrophils were classified based on different expression patterns as well (Figures S14C–F). Major information flow involved in antigen presentation and T cell activation (e.g., MHC‐II and MIF) were significantly higher in the classical and N1‐like neutrophils (Figures S14G and H). In contrast, the N2‐like neutrophils showed significantly higher information flow involved in tumour development (e.g., SPP1 and RESISTIN) (Figure S14I).^45^, ^46^ Increased stimulating interactions on T and B lymphocytes, including MIF‐(CD74+CD44)/(CD74+CXCR4) and HLA–DRA–CD4, were observed in the N1‐like and classical clusters (Figures S14J and K). However, the N2‐like cluster showed increased inhibiting signals like LAGLS9–CD44 (Figure S14L). Similar results were found in the external scRNA‐seq cohort of LUSC (Figure S15 and Table S9), validating the above findings.

Then the GSVA analysis comparing N1‐like and N2‐like neutrophils was conducted to further demonstrate the signalling pathways change. A reduction of TGF‐β signalling and an increment of interferon (IFN)‐α response was observed in N1‐like than N2‐like cluster, validating that N1‐like neutrophils may contribute to stimulating the anti‐tumour immune response (Figure 7L). GO enrichment analysis further illustrated that the modulated genes significantly enriched in inflammatory response and response to IFN‐α and IFN‐γ biological processes (Figure 7M). Collectively, an increment of stimulating signals and a reduction of inhibiting signals between N1‐like neutrophils and major immune effector cells were observed, and the IFN‐α and IFN‐γ signalling pathways showed a paramount role which drove the functional differences between N1‐like and N2‐like neutrophils, interpreting the potential biological processes underlying the prognostic effect of neutrophils on spatially adjacent T and B lymphocytes.

### 3.11 Spatially neighbouring M2‐like macrophages could blunt the activation of T lymphocytes

The transcriptomic characteristics of the macrophages were profiled, and three subtypes were identified (Figure S16A). The pan‐macrophage sub‐cluster was characterised by markers of cell growth regulation (e.g., EGR1) and tissue‐resident (e.g., NR4A3). The M1‐like subtype showed major expression levels of IFN (e.g., ISG15) and chemokine (e.g., CXCL10) inducible genes. The M2‐like subtype showed similar expression profiles to TAM (e.g., SPP1 and AREG) (Figure S16B).^47^

The overall information with macrophages as signal transmitters were similar among the three subtypes (MIF, MHC‐II and APP) (Figures S16C–E). M1‐like and M2‐like sub‐clusters showed an increased signal of angiogenesis than pan‐macrophage. Three subsets of macrophages both showed inhibiting signals like LGALS9–CD44/CD45, which may blunt the activation of T and B lymphocytes (Figures S16F and H). Stimulating signals involved in tumour angiogenesis, like VEGFA–VEGFR2 and VEGFB–VEGFR1, were observed. The M2‐like sub‐cluster additionally showed increased interactions with tumour cells (e.g., SPP1–CD44) (Figure S16H).^48^ Similar interaction networks were found in the external scRNA‐seq cohort of LUAD (Figure S17) and LUSC (Figure S18).

We further sought to investigate the signalling pathways change between M2‐like and M1‐like macrophages in the GSVA analysis, and an increment of genes regulated by MYC and genes involved in angiogenesis was observed (Figure 7N). GO enrichment analysis further indicated that the altered genes were enriched in chemotaxis and migration of leukocytes and negative regulation of immune effector process (Figure 7O). Collectively, the above analyses illustrated that spatially neighbouring M2‐like macrophages might blunt the activation of T lymphocytes and stimulate tumour growth by promoting angiogenesis.

**FIGURE 8:**
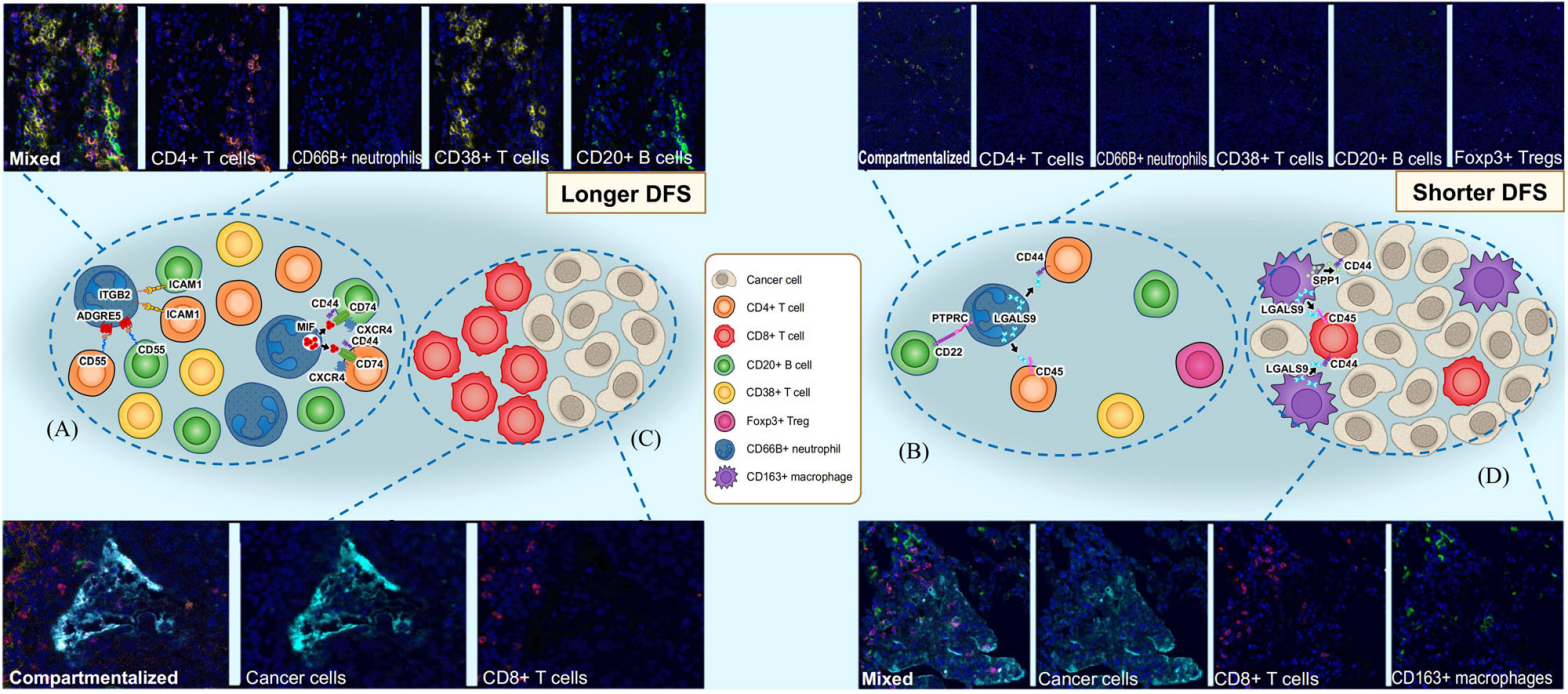
Proposed model of crosstalk between immune and tumour cells of non‐small cell lung cancer. Description of patients with a spatially mixed structure among CD4+ T cells, CD20+ B cells and N1‐like neutrophils. Spatially adjacent N1‐like neutrophils can boost the proliferation and activation of T and B lymphocytes, and such spatial pattern is associated with better disease‐free survival (DFS) (A). Depiction of patients with a spatially compartmentalised structure among immune cells (B). More FOXP3+ regulatory T cells and longer distances between neutrophils and CD4+ T cells and CD20+ B cells are observed, and such spatial pattern is associated with shorter DFS. Description of patients with a spatially compartmentalised structure between tumour and CD8+ T cells and have better DFS (C). Greater proximity and strengthened interactions between CD8+ T cells are also found. Depiction of patients with a spatially mixed structure between CD8+ T cells and tumour cells (D). Few CD8+ T cells scatter within these tumours and locate close to CD163+ (i.e., M2‐like) macrophages. Spatially neighbouring M2‐like macrophages can blunt the activation of T lymphocytes, and such spatial pattern is associated with shorter DFS.

We herein proposed the model of crosstalk between immune and tumour cells in TME based on the integrated findings from the spatial, receptor–ligand and downstream function analyses (Figure 8).

## 4 DISCUSSION

Utilising mIF coupled with deep learning‐based spatial assays, we identified various cell phenotypes and measured spatial cell infiltration patterns in the TME of 553 NSCLC cases. We further profiled the transcriptomic features of primary tumours and explored the putative cell interaction networks suggested by spatial analyses. Our findings leveraged insights into the spatial landscape and heterogeneity in TME of NSCLC, underscoring that the spatial relationships between immune and tumour cells might impact clinical outcomes.

The correlations between the spatial cell features and prognosis were comprehensively investigated. Proximity among CD20+ B cells, CD4+ T cells and CD38+ T cells was observed, and such spatial configuration has been shown to assist the development of TLS. Moreover, the presence of TLS in NSCLC has been reported to associate with better outcomes.^49^, ^50^ Proximity between CD66b+ neutrophils themselves, CD4+ T cells and CD66b+ neutrophils, CD38+ T cells and CD66b+ neutrophils and CD20+ B cells and CD66b+ neutrophils were observed in patients without recurrence than with recurrence, implying the vital role of neutrophils on spatially adjacent cells. To further understand the potential biological processes underlying the prognostic effects of these spatial patterns, then we explored the communication networks mediated by ligand–receptor pairs between different clusters of neutrophils and other cell types and conducted functional analyses using scRNA‐seq data. In brief, an increment of stimulating signals and a reduction of inhibiting signals between N1‐like neutrophils and major immune effector cells were observed, and the IFN‐α and IFN‐γ signalling pathways showed a paramount role which drove the functional differences between N1‐like and N2‐like neutrophils. Consequently, spatial proximity between T and B lymphocytes and N1‐like neutrophils, rather than N2‐like neutrophils, facilitated the effective anti‐tumour immune response, and accordingly, such spatial pattern was associated with a better prognosis (Figure 8A). Recent studies also showed that neutrophils could stimulate the adaptive immune response by presenting antigens to T cells and producing IFN‐γ in early‐stage LC.^51^, ^52^ They could also coordinate with B cells to mediate the antibody‐dependent cellular cytotoxicity process.^53^

On the contrary, we found that proximity between Tregs and neutrophils was associated with decreased infiltration levels of CD38+ T cells and CD20+ B cells. We also observed that increased distances between CD66b+ neutrophils and T and B lymphocytes were associated with significantly worse DFS (Figure 8B). Possible mechanisms may be that the neutrophils (mainly pro‐tumour subtype) induced by tumour cells could recruit Tregs to form immunosuppressive TME and promote angiogenesis, contributing to tumour invasion.^54^, ^55^

Consequently, our study showed that the prognostic effects of neutrophils could be influenced by their spatial location and neighbour cells or milieu, apart from their amounts, whereas few studies have evaluated this issue before. It is acknowledged that due to the lack of distinctive cell surface markers, different functional phenotypes were utilised to define different subsets of neutrophils.^56^ Generally, anti‐tumour (N1) and pro‐tumour (N2) phenotypes of neutrophils were identified and adopted across different cancer types.^57^ The phenotypes of neutrophils also alter during the evolution from marrow resident to circulatory and senescent processes.^58^ Therefore, the conflicting results reported by different studies could be attributed to different phenotypes of neutrophils. Recently, with the advances of high‐resolution approaches, like scRNA‐seq, novel and crucial phenotypes of neutrophils were identified, contributing to better understanding their dual roles in TME.^43^, ^59^

We found CD133+ CSCs were the most abundant cell populations in TN and closer proximity between CD133+ CSCs predicted significantly worse DFS, similar to the study by Wang et al.^14^ that more connections between tumour cells were a poor prognostic factor of LC. Meanwhile, closer distance between CD133+ CSCs was more significantly correlated with recurrence than with more CD133+ CSCs amounts alone, highlighting that spatial analysis could produce a higher‐order of useful information than simply measuring cell amounts. Furthermore, as the second most abundant cell lineages, M2 macrophages were observed to locate close to CD133+ CSCs and CD8+ T cells. Signalling pathways change between M2‐like and M1‐like macrophages were evaluated, and an increment of genes regulated by MYC and genes involved in angiogenesis was observed. Functional analysis further indicated that the altered genes were enriched in negative regulation of immune effector process, indicating that spatially neighbouring M2 macrophages may blunt the activation of T lymphocytes and stimulate tumour growth by promoting angiogenesis, and accordingly, such spatial pattern was associated with a poor prognosis (Figure 8D). Previous studies also supported that interactions between M2 macrophages and CD133+ CSCs can mediate the exhaustion of cytotoxic CD8+ T cells and further cause tumour progression.^38^, ^60^ Moreover, we found that greater proximity between CD8+ T cells improved DFS. Such trends have been confirmed in other cancer types as well, like colorectal cancer^61^ and BC.^62^ The findings mentioned above implied that the spatial compartmentalisation between tumour cells and CD8+ T cells (Figure 8C), instead of mixing (Figure 8D), was correlated with better DFS. However, the explicit biological events behind the spatial interaction networks were still unclear, and cellular and animal function experimental studies are needed to validate and support our findings.

We then developed a prognostic model integrating spatial and quantitative features that characterised the TME and impacted complex biological pathways. The IRRS was an independent and vicious indicator of recurrence in IA∼IIIB NSCLC patients. The IRRS system displayed relatively high accuracy in predicting recurrence across cTNM stages, contributing to identifying patients at high risk of early relapse and may profit from additional systemic therapies.^63^ Moreover, despite without statistical significance, the IRRS model manifested better predictive ability than the cTNM system. The IRRS model presented in the current study has several advantages compared with other prognostic models. First, studies built on cell population estimation algorithms (e.g., CIBERSORT and ssGSEA) only used a limited set of genes. However, gene expression profiles do not necessarily associate with protein levels, and different transcriptional activity among different cell populations can deflate or inflate the appraisal of inactive or active cells potentially. Second, algorithms like CIBERSORT encountered statistical collinearity when estimating the immune cells.^64^ Third, studies based on traditional transcriptome or microarray data could not evaluate the localisation of identified cells.

The immune checkpoint blockade therapies have reformed the treatment of NSCLC over the years. Strikingly, the immune profiles, including immunotherapeutic markers, in different IRRS groups were remarkably different. Patients with low‐IRRS displayed an ‘immune‐hot’ subtype, showing the abundant infiltration of CD4+ T cells and CD8+ T cells, and a higher expression of PD‐L1 was also detected. As is well known, T lymphocytes are the core of anti‐tumour immunity and offer backup sources for immunotherapy.^65^ Consequently, with a great abundance of T cells, low‐IRRS patients may benefit from immunotherapy potentially. High‐IRRS patients were characterised by rich infiltration of macrophages and subsets, and may profit from emerging therapies targeting TAMs.^66^ More strikingly, the spatial landscapes between the high‐IRRS and the low‐IRRS groups were distinct. We found that the crosstalk between CSCs was strengthened, whereas the crosstalk between CD8+ T cells was weakened in high‐IRRS patients, hinting at the impaired immune response. Significantly longer distances among CD20+ B cells, CD4+ T cells, and neutrophils were also observed in the high‐IRRS group.

Apart from its prognostic value of patients’ outcomes, spatial features mining from images also showed potency in early diagnosis of cancer, predicting histologic grade and treatment response. Li and colleagues^67^ have established a diagnosis tool for gastric cancer using spatial features from the fluorescence hyperspectral images and reported an overall accuracy of 96.5%. Lagree et al.^68^ demonstrated that the spatial characteristics of tumour nuclei in HE images helped improve the classification performance of histologic grade of BC. Xie et al.^69^ reported a computational marker derived from HE images that could predict immunotherapy response of LC with an AUC of 0.69. However, most of the models were developed at a single centre and whether these findings could generalise to external datasets remained challenging. Notwithstanding the limitations, previous studies have done a great job of formulating a framework for future studies.

Our study has several distinct advantages. First, the mIF method facilitated detection of multiple protein targets in a single‐slide simultaneously, providing a unique perspective of the immune landscape.^3^ Additionally, the dataset used in the deep learning model included more than 2700 mIF images of 553 LC patients, that is, a median of 4–5 images were analysed per patient, therefore reducing the bias from blank regions or tissue artefacts that may confound the downstream analyses. Besides, the deep learning model exploited the Delaunay triangulation algorithm without requiring arbitrary definitions of connections, providing a better characterisation of cellular proximity in the TME.^70^ Furthermore, the cell communication networks were supported by spatial analyses from mIF graphs and functional analyses from internal and external scRNA‐seq datasets.

Despite these advantages, our study has some limitations. First, our study did not include markers for other non‐immune components in TME, like fibroblasts.^71^ Second, the marker for labelling tumour cells lacked specificity. Although CD133 is well‐recognised as a stem cell marker of CSCs, it can also express on normal tissues like immature progenitor cells.^3^, ^72^ Also, CD38 is not only expressed on activated T cells, but it can also express on plasma cells and haematological tissues.^73^ Likewise, despite being widely adopted in other studies,^74^, ^75^ we acknowledged that our method to annotate TN and TS was less objective or precise than directly staining unique markers in TN and TS (e.g., pan keratin and collagen). Third, we did not detect the expression patterns of PD1, which may help estimate the competence of immune cells, like non‐exhausted (CD8+PD‐1−) or exhausted (CD8+PD‐1+) T cells. Moreover, we merely sketched and differentiated the regions of TN and TS in the whole‐slide images, without separately analysing the cell composition in the fibrotic or the necrotic regions. Additional limitations included a single institute setting and a lack of external validation of the IRRS model. Future studies in multi‐centre settings across populations will be needed to evaluate the strength and validity of our observations.

Future studies should focus on the dynamic change in the composition and spatial distribution of cells during the treatment process, for example, neoadjuvant immunotherapy and adjuvant immunotherapy. Furthermore, spatial transcriptomics, in vivo and in vitro experiments are needed for precisely elucidating the rebuilding of the heterogeneous and complex TME in response to different treatment options. As for the IRRS model, future research with treatment information and large sample size is required to assess its value for predicting treatment response.

## 5 CONCLUSIONS

We developed a framework to analyse the cell interaction networks in TME based on mIF images, which is efficient for understanding the biological events in which spatial interplays among cells determine functionality. Several spatial architectures, including mixing distributed patterns among CD20+ B cells, CD4+ T cells and N1‐like neutrophils, and spatial compartmentalisation between CD133+ CSCs and CD8+ T cells, were underscored to play a vital role in immune response and helped stratify patients who may have an early relapse.

## CONFLICT OF INTEREST

The authors declare no potential conflicts of interest.

## Supporting information

Supporting information.
**Supplementary figure 1**. The procedure of segmenting tissues and cells on whole‐slide multiplex immunofluorescence images in the inForm software.

Supporting information.
**Supplementary figure 2**. Examples of matched and unmatched objects for the segmentation evaluation of the Stardist model. Cells circled by the red boxes meant the “matched” objects, whereas cells circled by the green boxes were the “unmatched” objects (A). Demonstration of the segmentation process in the Stardist model (B).

Supporting information.
**Supplementary figure 3**. The visual example of the cell spatial organization‐related features in the tumor microenvironment, including the number of connections and the average lengths of connections between two cell types.

Supporting information.
**Supplementary figure 4**. Correlation analyses between different cell types in tumor nest and tumor stroma based on the quantitative parameters.

Supporting information.
**Supplementary** **figure** **5**. Correlation analyses between cell composition and spatial distribution. Spearman rank correlation matrix demonstrated the associations between quantitative and spatial variables (A). CD8+ T cells (B‐C) and CD133+ cells (D‐E) showed significant associations between cell composition and spatial distribution.

Supporting information.
**Supplementary figure 6**. Discrepancies of immune structure in tumor microenvironment between different histology subtypes of lung cancer. Differences in spatial cell location (A‐B) and cell infiltration (C‐D) between lung adenocarcinoma and squamous carcinoma

Supporting information.
**Supplementary figure 7**. Associations between cell spatial distribution and patients' clinical features. Discrepancies of spatial variables across the different cTNM stage (A‐B), N stage (C‐D), and T stage (E‐F) as evaluated by the Kruskal‐Wallis H test. *P < 0.05; **P < 0.01; ns, non‐significant.

Supporting information.
**Supplementary figure 8**. Prognostic effects in disease‐free survival of spatial and quantitative variables. The multivariate Cox regression analysis evaluated the prognostic significance of spatial features with adjustments for age, sex, N stage, T stage, vascular cancer embolus, and the number of lymph nodes resection (A‐B). Quantitative features with significant prognostic effects of as evaluated by the univariate Cox regression analysis (C).

Supporting information.
**Supplementary figure 9**. Validation of the immune‐related risk score (IRRS) model in the testing cohort. The distribution of IRRS, recurrence status, and immune profiles of patients (A). Receiver operating characteristic (ROC) curves and area under curve (AUC) values of IRRS model for predicting recurrence risk at 1, 3, and 5 years (B‐C). The cell infiltration and spatial location disparities between high and low IRRS subgroups as evaluated by the Kruskal‐Wallis H test (D). The disparities in the clinical characteristics between high and low IRRS subgroups as evaluated by the Chi‐square test (E‐K). *P < 0.05; **P < 0.01; ***P < 0.001; ****P < 0.0001; ns, non‐significant.

Supporting information.
**Supplementary figure 10**. Validation of the immune‐related risk score (IRRS) system in the entire cohort. The distribution of IRRS, disease‐free survival, and immune profiles of patients (A). Receiver operating characteristic (ROC) curves and area under curve (AUC) values of IRRS system for predicting recurrence risk at 1, 3, and 5 years (B‐C). The cell infiltration and spatial location disparities between high and low IRRS subgroups as evaluated by the Kruskal‐Wallis H test (D). The disparities of clinical characteristics between high and low IRRS subgroups as evaluated by the Chi‐square test (E‐K). *P < 0.05; **P < 0.01; ***P < 0.001; ****P < 0.0001; ns, non‐significant.

Supporting information.
**Supplementary figure 11**. Comparison of the predictive accuracy of the immune‐related risk (IRRS) model and cTNM system. Predictive performance of the IRRS model and cTNM system in the training (A), testing (B), and entire (C) cohort.

Supporting information.
**Supplementary figure 12**. Cell composition in the tumor microenvironment for each patient as evaluated by single‐cell RNA sequencing. The UMAP plots (A‐D) and canonical gene markers (E‐F) to label different cell clusters for each patient.

Supporting information.
**Supplementary figure 13**. Profiling the composition of epithelial cells through single‐cell RNA sequencing. Tumor cells and non‐malignant epithelial cells as represented in the UMAP plot (A). Canonical marker genes, including MMP7, MMP9, MMP13, and HOXB2, were used to label and distinguish between tumor cells and non‐malignant epithelial cells (B).

Supporting information.
**Supplementary figure 14**. External single‐cell RNA sequencing cohort of lung adenocarcinoma reveals cell composition and cell interaction networks in tumor microenvironment. Eleven major cell lineages as represented in the UMAP plot (A). Canonical gene markers to label clusters in the UMAP plot, including NKG7 for natural killer cells, CD3D for T cells, CD163 for macrophages, G0S2 for neutrophils, CD83 for dendritic cells, EPCAM for stem cells, MZB1 for B cells, COL3A1 for fibroblasts, KRT7 for epithelial cells, TPSAB1 for mast cells, and EMCN for endothelial cells (B). Classical, N1‐like, and N2‐like neutrophils as represented in the UMAP plot (C). Canonical gene markers to label three subclusters of neutrophils, including IRF4 and IRF8 for N1‐like neutrophils (D), S100A12 and FCN1 for classical neutrophils (E), and CSTB and LGALS3 for N2‐like neutrophils (F). The overall information flow of each signal pathway in classical (G), N1‐like (H), and N2‐like (I) neutrophils. Communication probabilities mediated by ligand‐receptor pairs with classical (J), N1‐like (K), and N2‐like (L) neutrophils as signal receivers.

Supporting information.
**Supplementary figure 15**. External single‐cell RNA sequencing cohort of squamous cell lung cancer reveals cell composition and cell interaction networks in tumor microenvironment. Nine major cell lineages as represented in the UMAP plot (A). Canonical gene markers to label clusters in the UMAP plot, including CD3D for T cells, NKG7 for natural killer cells, CD68 for macrophages, S100A9 for neutrophils, SLC18A2 for mast cells, KRT8 for epithelial cells, COL1A1 for fibroblasts, CD79A for B cells, CST3 for dendritic cells (B). Classical, N1‐like, and N2‐like neutrophils as represented in the UMAP plot (C). Canonical gene markers to label three subsets of neutrophils, including S100A8 and FCN1 for classical neutrophils (D), C15orf48 and HLA‐DQB2 for N1‐like neutrophils (E), and CSTB and LGALS3 for N2‐like neutrophils (F). The overall information flow of each signal pathway in classical (G), N1‐like (H), and N2‐like (I) neutrophils. Communication probabilities mediated by ligand‐receptor pairs with classical (J), N1‐like (K), and N2‐like (L) neutrophils as signal receivers.

Supporting information.
**Supplementary figure 16**. Results of the CellChat analyses with macrophages as signal transmitters based on internal single‐cell RNA sequencing cohort of lung adenocarcinoma. UMAP plot (A) and heatmap of differentially expressed gene sets in macrophage subsets (B). The overall information flow of each signal pathway in pan‐macrophages (C), M1‐like (D), and M2‐like (E) macrophages. Communication probabilities mediated by ligand‐receptor pairs in pan‐macrophages (F), M1‐like (G), and M2‐like (H) macrophages.

Supporting information.
**Supplementary figure 17**. Results of the CellChat analyses with macrophages as signal transmitters based on external single‐cell RNA sequencing cohort of lung adenocarcinoma. UMAP plot (A) and canonical gene markers to label three subsets of macrophages, including MARCO and CD52 for pan‐macrophages (B), SLC40A1 and FOLR2 for M1‐like macrophages (C), and CCL18 and CXCL8 for M2‐like macrophages (D). The overall information flow of each signal pathway in pan‐macrophages (E), M1‐like (F), and M2‐like (G) macrophages. Communication probabilities mediated by ligand‐receptor pairs in pan‐macrophages (H), M1‐like (I), and M2‐like (J) macrophages.

Supporting information.
**Supplementary figure 18**. Results of the CellChat analyses with macrophages as signal transmitters based on external single‐cell RNA sequencing cohort of squamous cell lung cancer. UMAP plot (A) and canonical gene markers to label three subsets of macrophages, including CD14 and C3AR1 for pan‐macrophages (B), TMEM176B and FOLR2 for M1‐like macrophages (C), and FN1 and SPP1 for M2‐like macrophages (D). The overall information flow of each signal pathway in pan‐macrophages (E), M1‐like (F), and M2‐like (G) macrophages. Communication probabilities mediated by ligand‐receptor pairs in pan‐macrophages (H), M1‐like (I), and M2‐like (J) macrophages.

Supporting information.
**Supplementary table 1**. Information of primary antibodies used in the multiplex immunofluorescence test.

Supporting information.
**Supplementary table 2**. Markers and corresponding cell types in multiplex immunofluorescence detection.

Supporting information.
**Supplementary table 3**. Characteristics of the four non‐small cell lung cancer patients included in the single‐cell RNA sequencing.

Supporting information.
**Supplementary table 4**. Cell spatial features in the lung cancer microenvironment of the included patients.

Supporting information.
**Supplementary table 5**. Demographic characteristics and immune‐related risk score of the included patients.

Supporting information.
**Supplementary table 6**. Univariate Cox regression model demonstrated the prognostic effects of the immune‐related risk score model.

**Supplementary table 7**. Marker genes for annotation of major cell types in internal single‐cell RNA sequencing dataset of lung adenocarcinoma.

Supporting information.
**Supplementary table 8**. Marker genes for annotation of major cell types in external single‐cell RNA sequencing dataset of lung adenocarcinoma.

Supporting information.
**Supplementary table 9**. Marker genes for annotation of major cell types in external single‐cell RNA sequencing dataset of squamous cell lung cancer.

Supporting information.
Supporting information.
**Supplementary data 1**. Multiplex immunofluorescence images used to train the algorithm in the inform software and the corresponding clinical characteristics of these patients.

## Data Availability

Data used to support the findings of this study are available from the corresponding author upon reasonable request.
