# Supplementary figures and images for: Multiplex immunofluorescence and single‐cell transcriptomic profiling reveal the spatial cell interaction networks in the non‐small cell lung cancer microenvironment
Source: Clin Transl Med. 2023 Jan 1;13(1):e1155. doi: 10.1002/ctm2.1155 (PMC9806015; doi:10.1002/ctm2.1155)

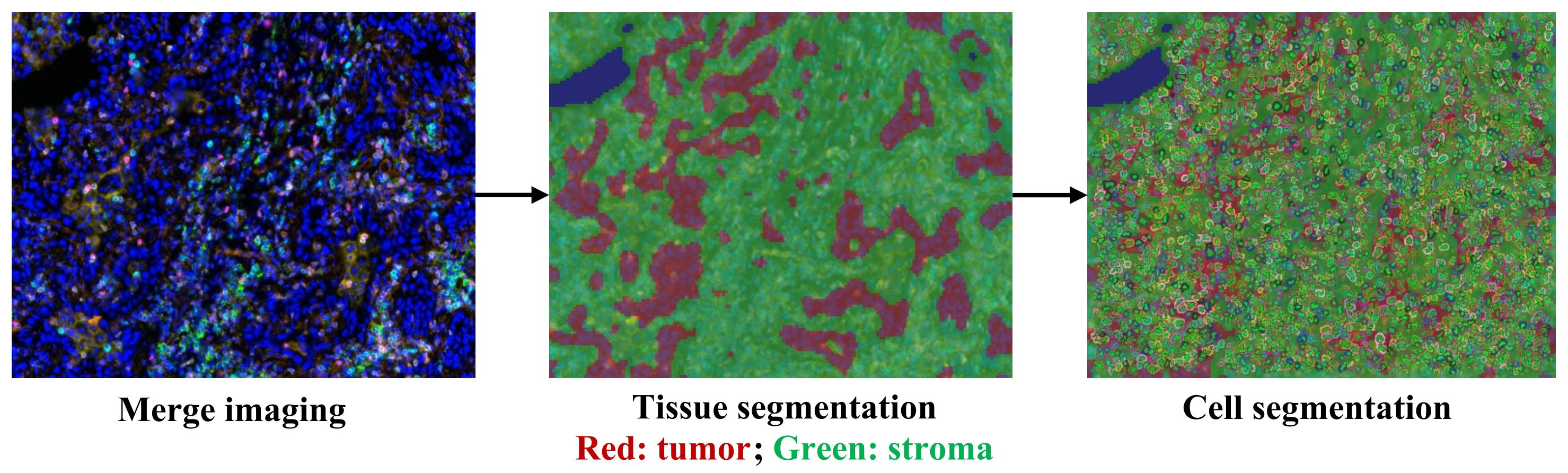

Supplement: Supplementary file 1 — Supporting information. Supplementary figure 1. The procedure of segmenting tissues and cells on whole‐slide multiplex immunofluorescence images in the inForm software. [file CTM2-13-e1155-s017.jpg]

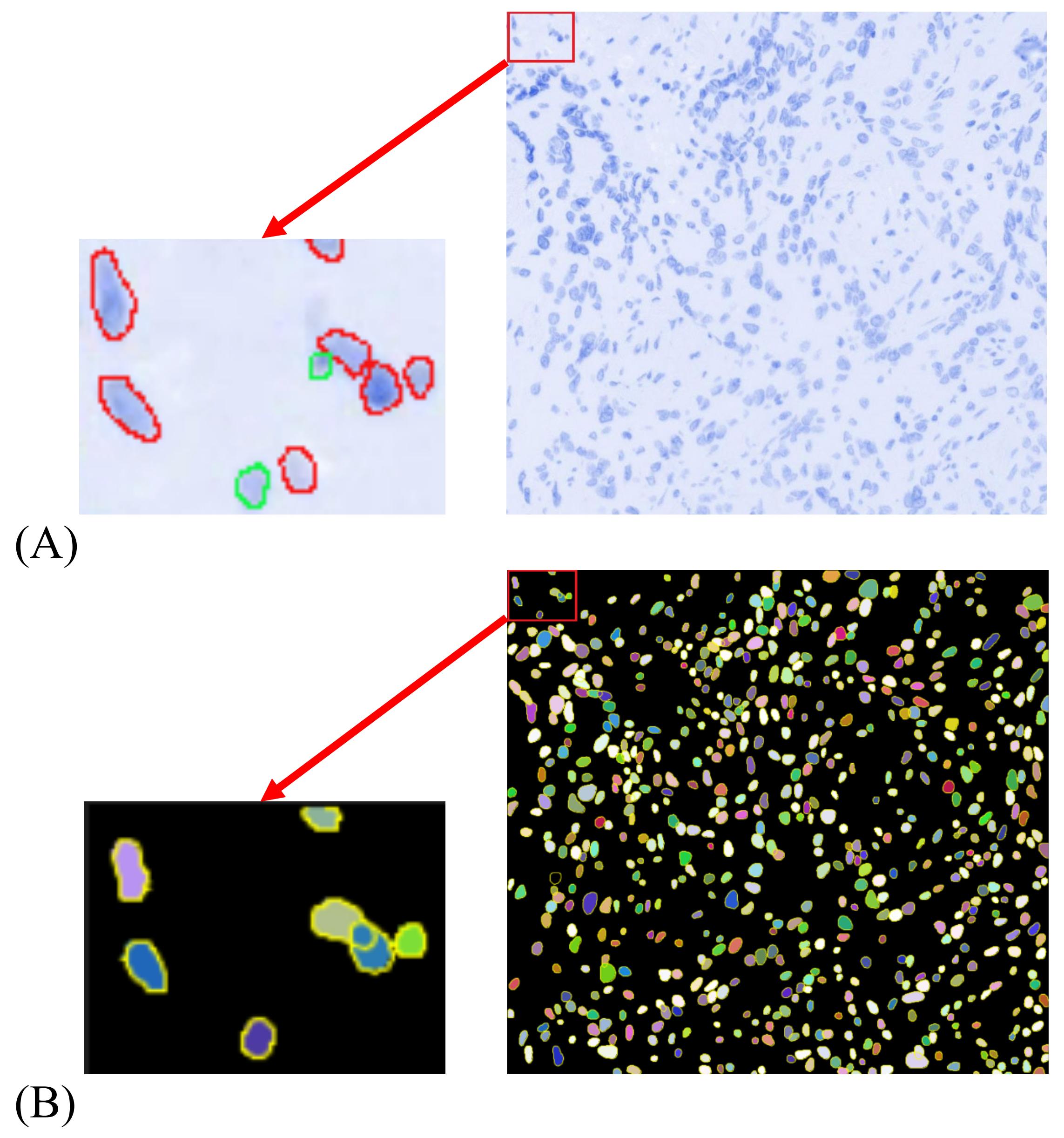

Supplement: Supplementary file 2 — Supporting information. Supplementary figure 2. Examples of matched and unmatched objects for the segmentation evaluation of the Stardist model. Cells circled by the red boxes meant the “matched” objects, whereas cells circled by the green boxes were the “unmatched” objects (A). Demonstration of the segmentation process in the Stardist model (B). [file CTM2-13-e1155-s018.jpg]

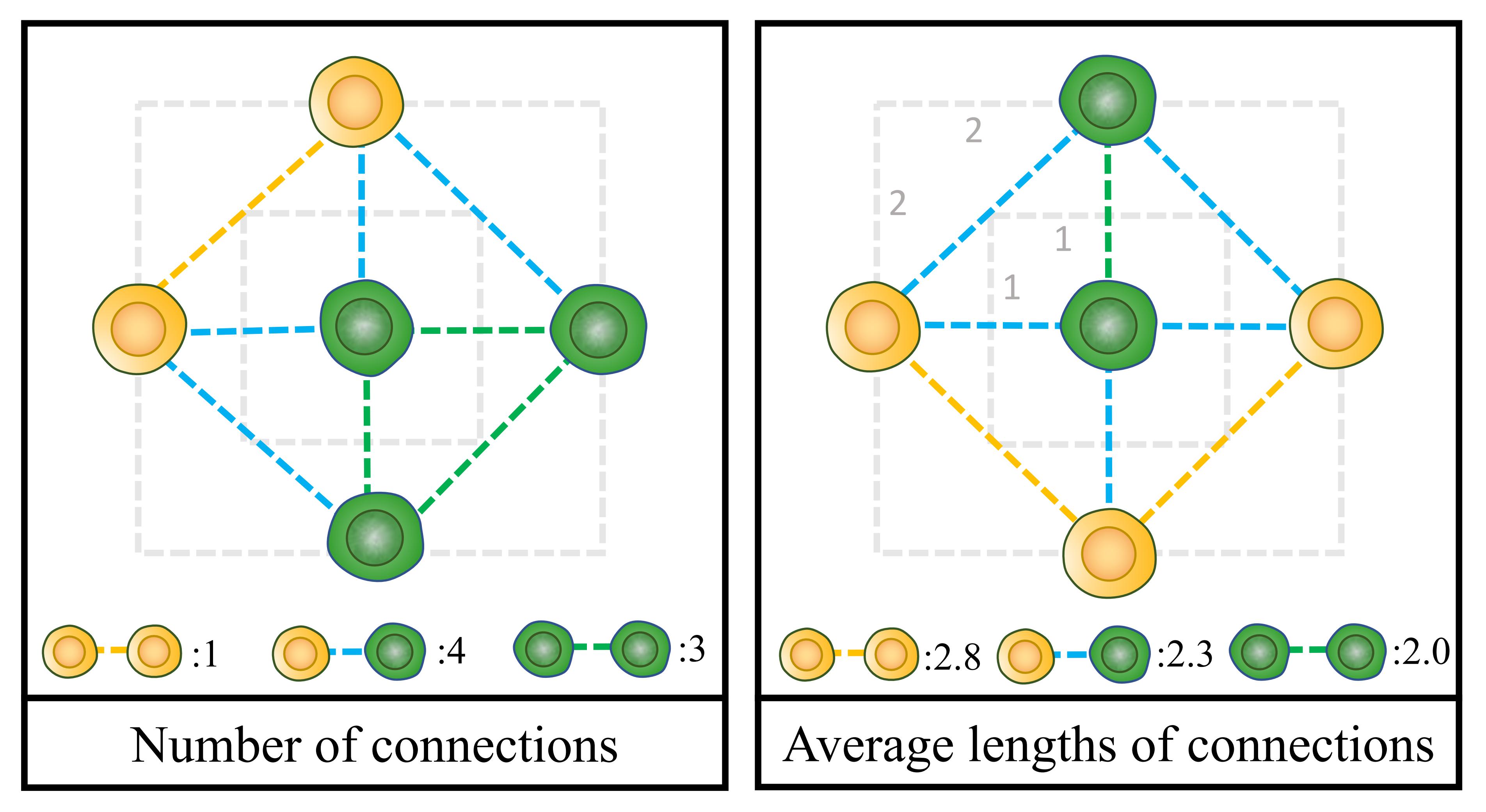

Supplement: Supplementary file 3 — Supporting information. Supplementary figure 3. The visual example of the cell spatial organization‐related features in the tumor microenvironment, including the number of connections and the average lengths of connections between two cell types. [file CTM2-13-e1155-s006.jpg]

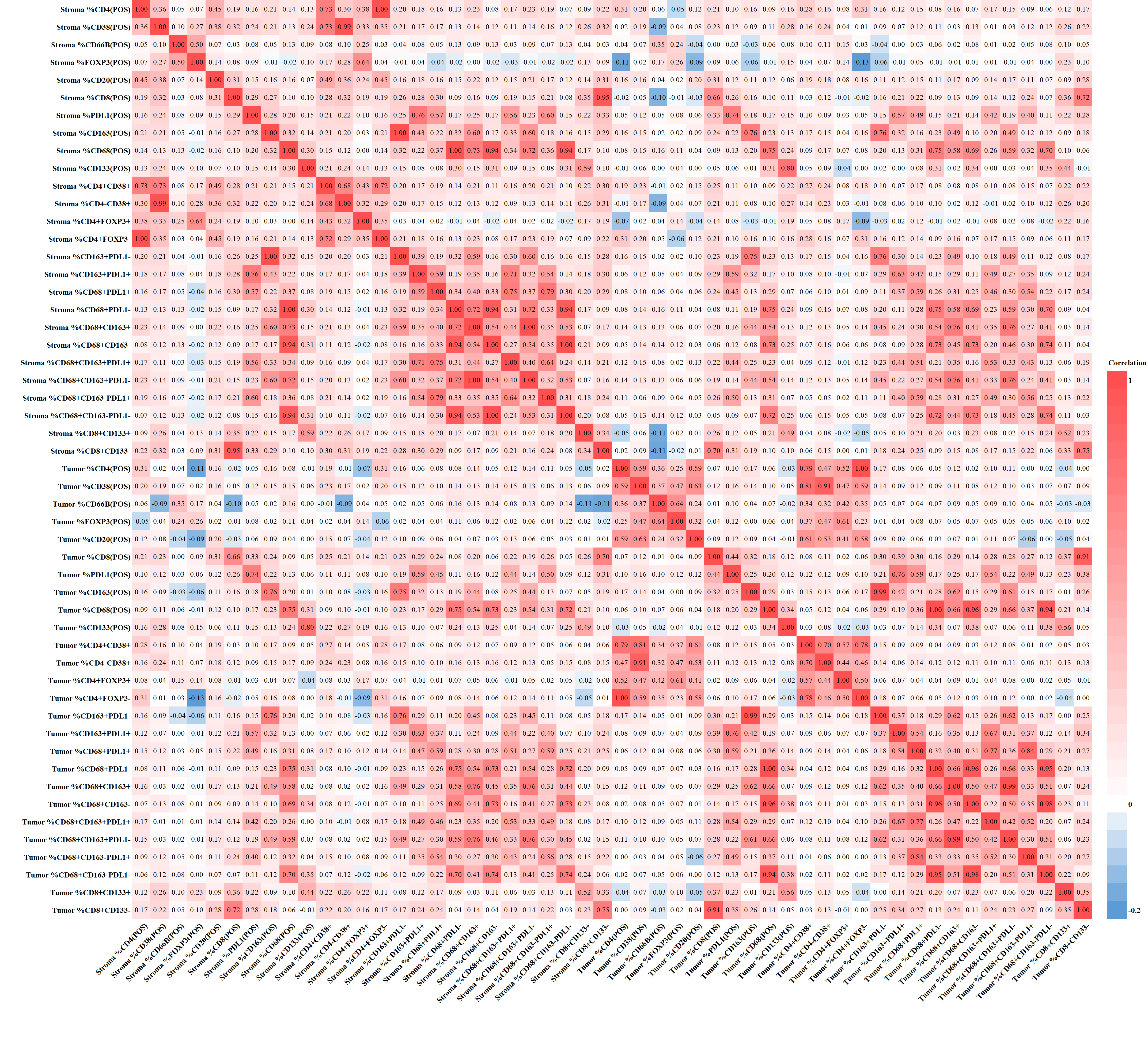

Supplement: Supplementary file 4 — Supporting information. Supplementary figure 4. Correlation analyses between different cell types in tumor nest and tumor stroma based on the quantitative parameters. [file CTM2-13-e1155-s025.jpg]

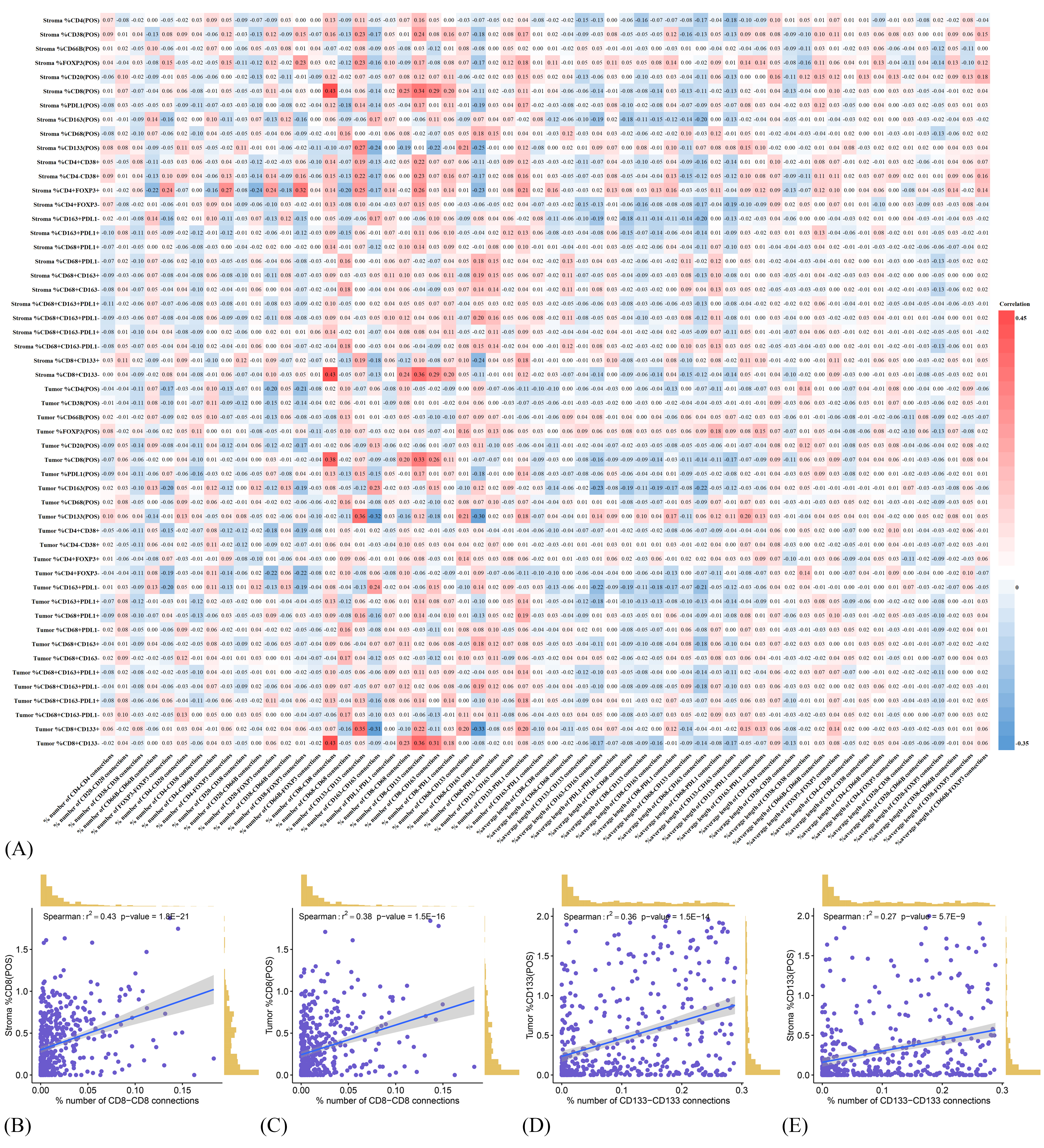

Supplement: Supplementary file 5 — Supporting information. Supplementary figure 5. Correlation analyses between cell composition and spatial distribution. Spearman rank correlation matrix demonstrated the associations between quantitative and spatial variables (A). CD8+ T cells (B‐C) and CD133+ cells (D‐E) showed significant associations between cell composition and spatial distribution. [file CTM2-13-e1155-s008.jpg]

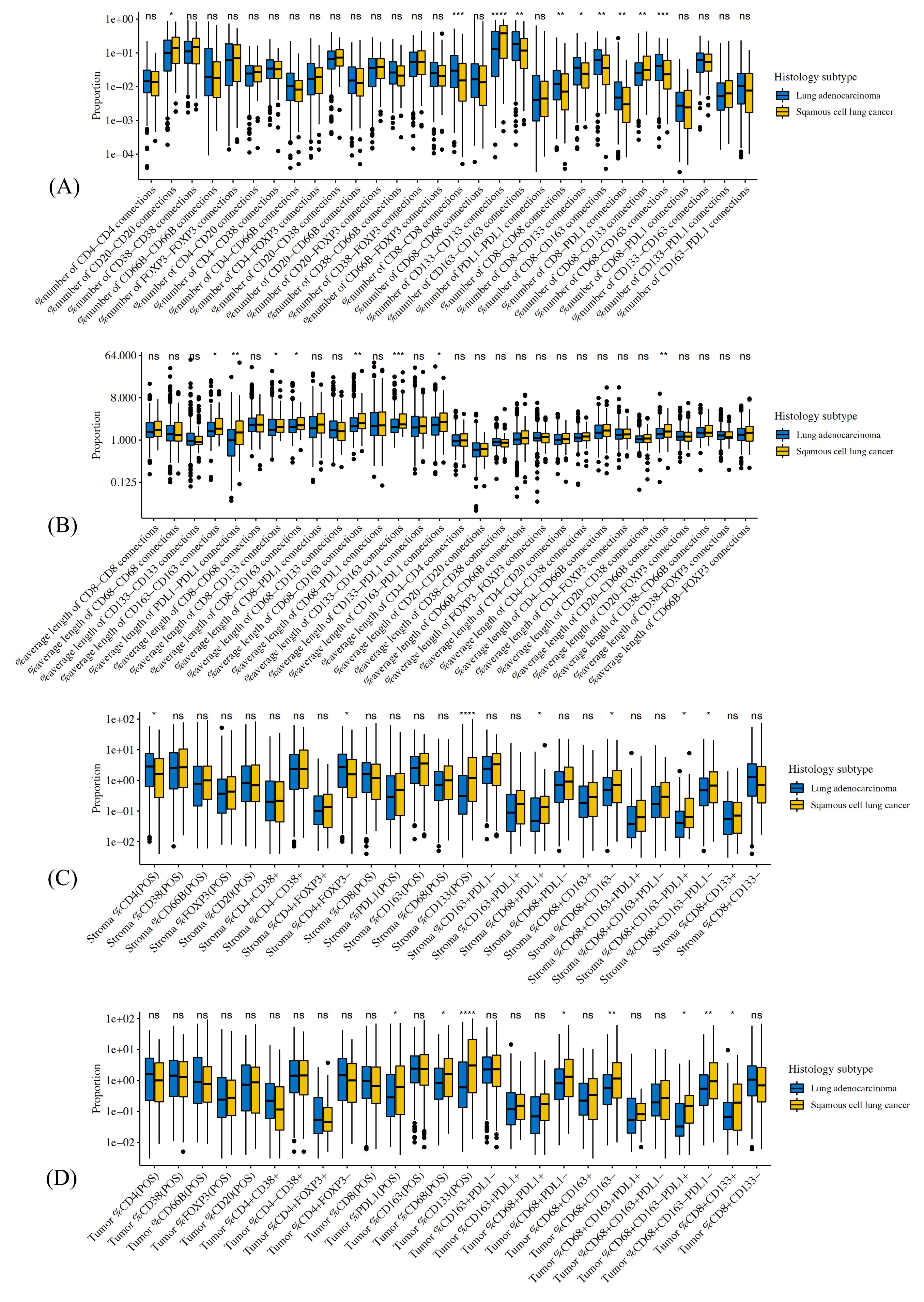

Supplement: Supplementary file 6 — Supporting information. Supplementary figure 6. Discrepancies of immune structure in tumor microenvironment between different histology subtypes of lung cancer. Differences in spatial cell location (A‐B) and cell infiltration (C‐D) between lung adenocarcinoma and squamous carcinoma [file CTM2-13-e1155-s021.jpg]

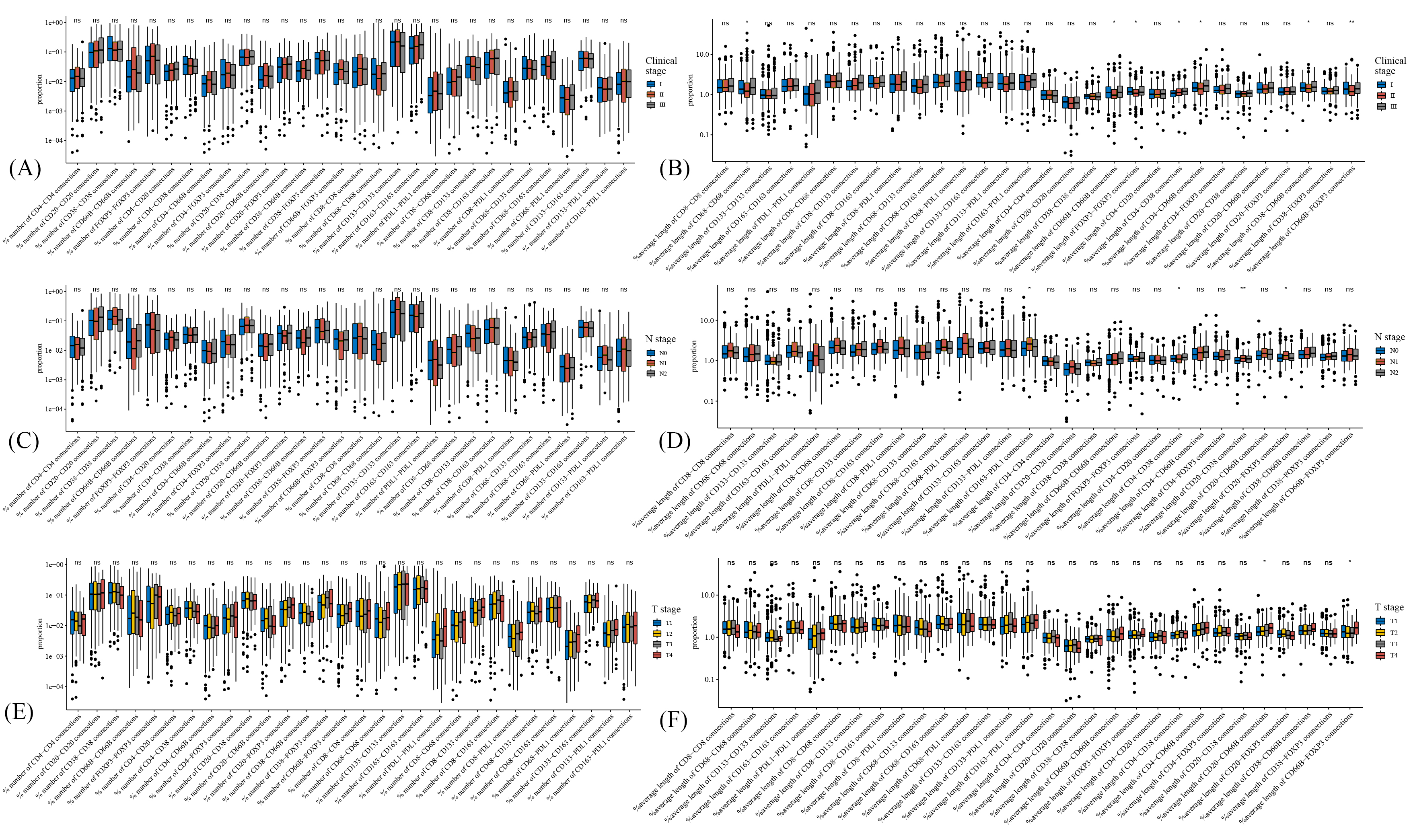

Supplement: Supplementary file 7 — Supporting information. Supplementary figure 7. Associations between cell spatial distribution and patients' clinical features. Discrepancies of spatial variables across the different cTNM stage (A‐B), N stage (C‐D), and T stage (E‐F) as evaluated by the Kruskal‐Wallis H test. *P < 0.05; **P < 0.01; ns, non‐significant. [file CTM2-13-e1155-s011.jpg]

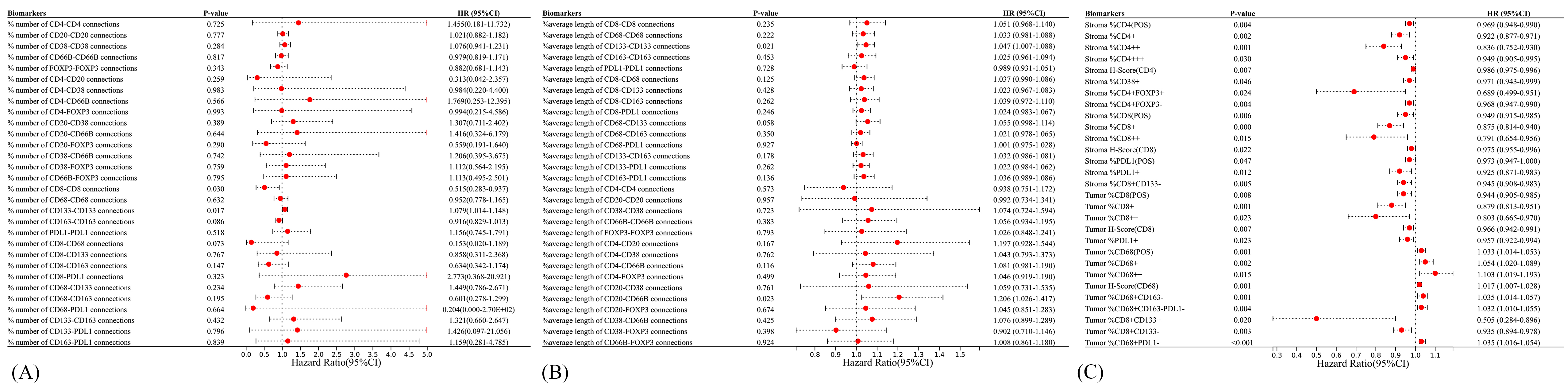

Supplement: Supplementary file 8 — Supporting information. Supplementary figure 8. Prognostic effects in disease‐free survival of spatial and quantitative variables. The multivariate Cox regression analysis evaluated the prognostic significance of spatial features with adjustments for age, sex, N stage, T stage, vascular cancer embolus, and the number of lymph nodes resection (A‐B). Quantitative features with significant prognostic effects of as evaluated by the univariate Cox regression analysis (C). [file CTM2-13-e1155-s010.jpg]

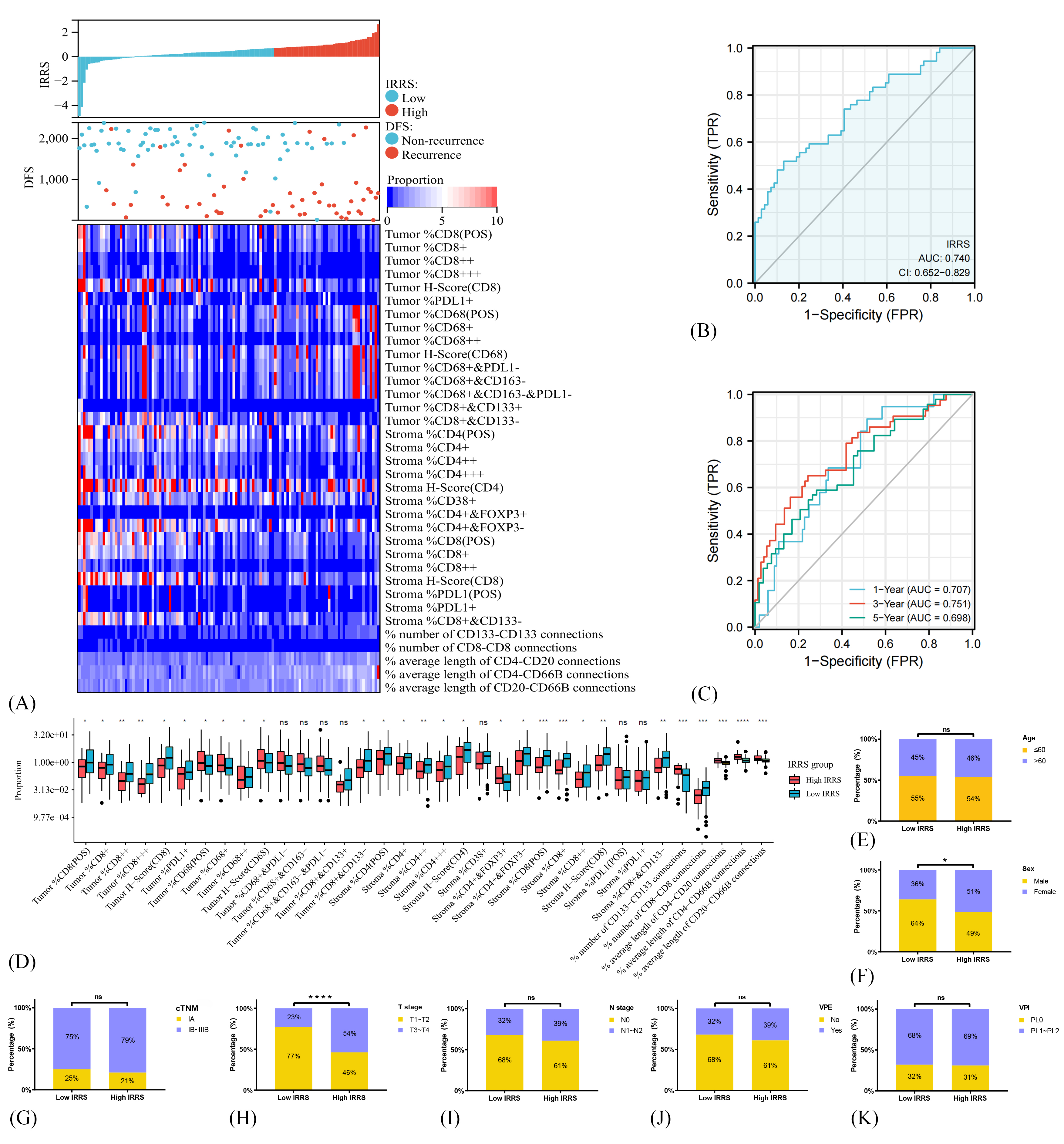

Supplement: Supplementary file 9 — Supporting information. Supplementary figure 9. Validation of the immune‐related risk score (IRRS) model in the testing cohort. The distribution of IRRS, recurrence status, and immune profiles of patients (A). Receiver operating characteristic (ROC) curves and area under curve (AUC) values of IRRS model for predicting recurrence risk at 1, 3, and 5 years (B‐C). The cell infiltration and spatial location disparities between high and low IRRS subgroups as evaluated by the Kruskal‐Wallis H test (D). The disparities in the clinical characteristics between high and low IRRS subgroups as evaluated by the Chi‐square test (E‐K). *P < 0.05; **P < 0.01; ***P < 0.001; ****P < 0.0001; ns, non‐significant. [file CTM2-13-e1155-s016.jpg]

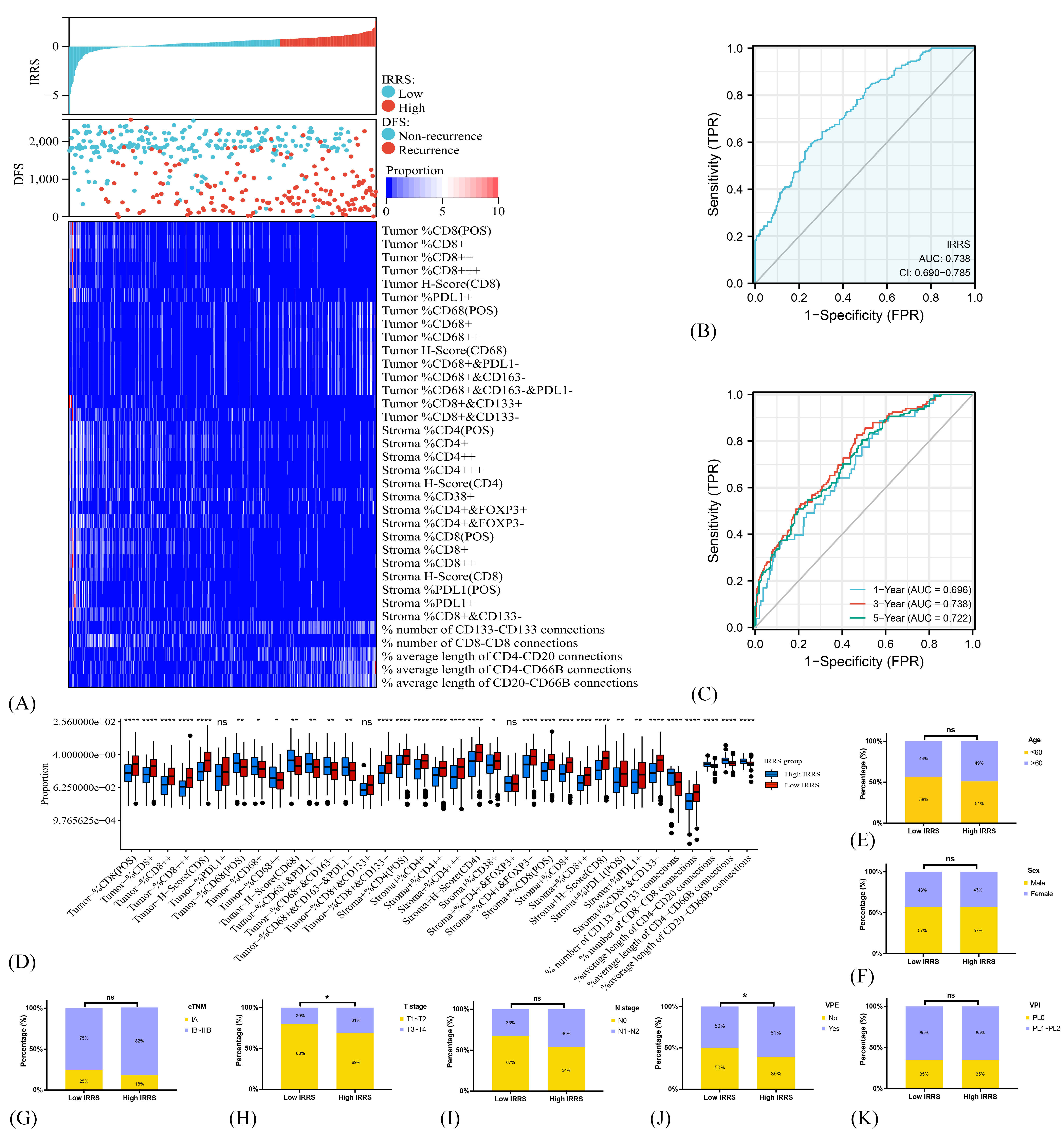

Supplement: Supplementary file 10 — Supporting information. Supplementary figure 10. Validation of the immune‐related risk score (IRRS) system in the entire cohort. The distribution of IRRS, disease‐free survival, and immune profiles of patients (A). Receiver operating characteristic (ROC) curves and area under curve (AUC) values of IRRS system for predicting recurrence risk at 1, 3, and 5 years (B‐C). The cell infiltration and spatial location disparities between high and low IRRS subgroups as evaluated by the Kruskal‐Wallis H test (D). The disparities of clinical characteristics between high and low IRRS subgroups as evaluated by the Chi‐square test (E‐K). *P < 0.05; **P < 0.01; ***P < 0.001; ****P < 0.0001; ns, non‐significant. [file CTM2-13-e1155-s002.jpg]

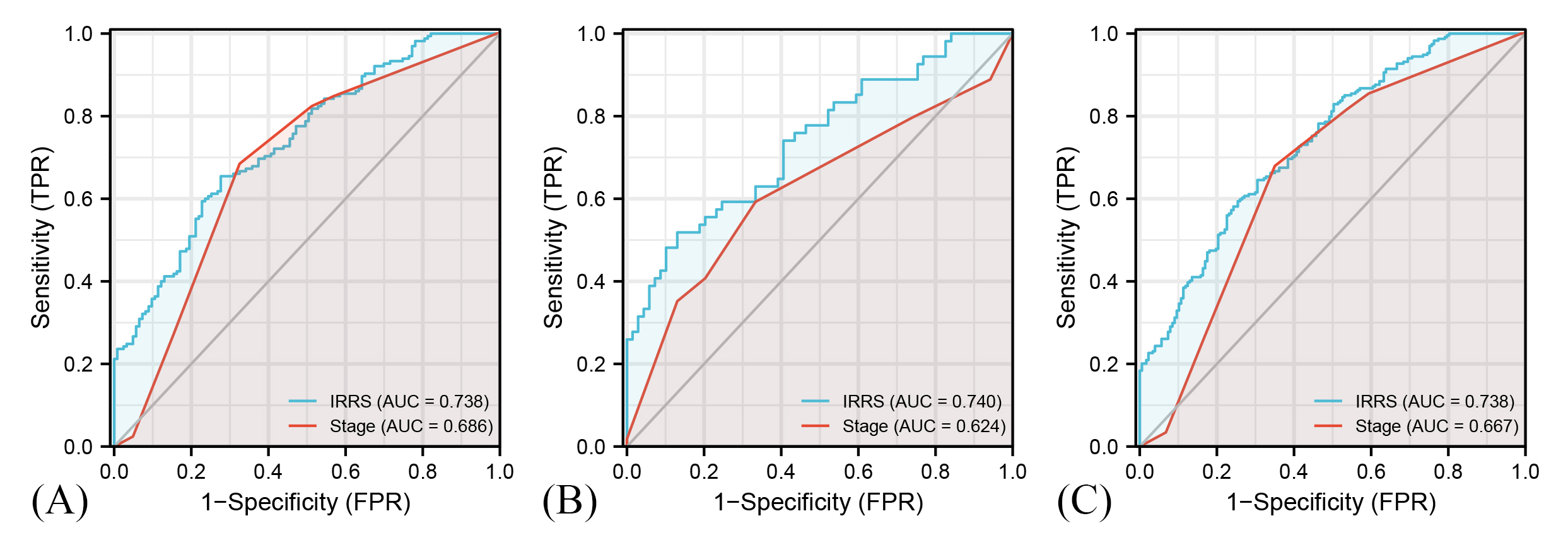

Supplement: Supplementary file 11 — Supporting information. Supplementary figure 11. Comparison of the predictive accuracy of the immune‐related risk (IRRS) model and cTNM system. Predictive performance of the IRRS model and cTNM system in the training (A), testing (B), and entire (C) cohort. [file CTM2-13-e1155-s019.jpg]

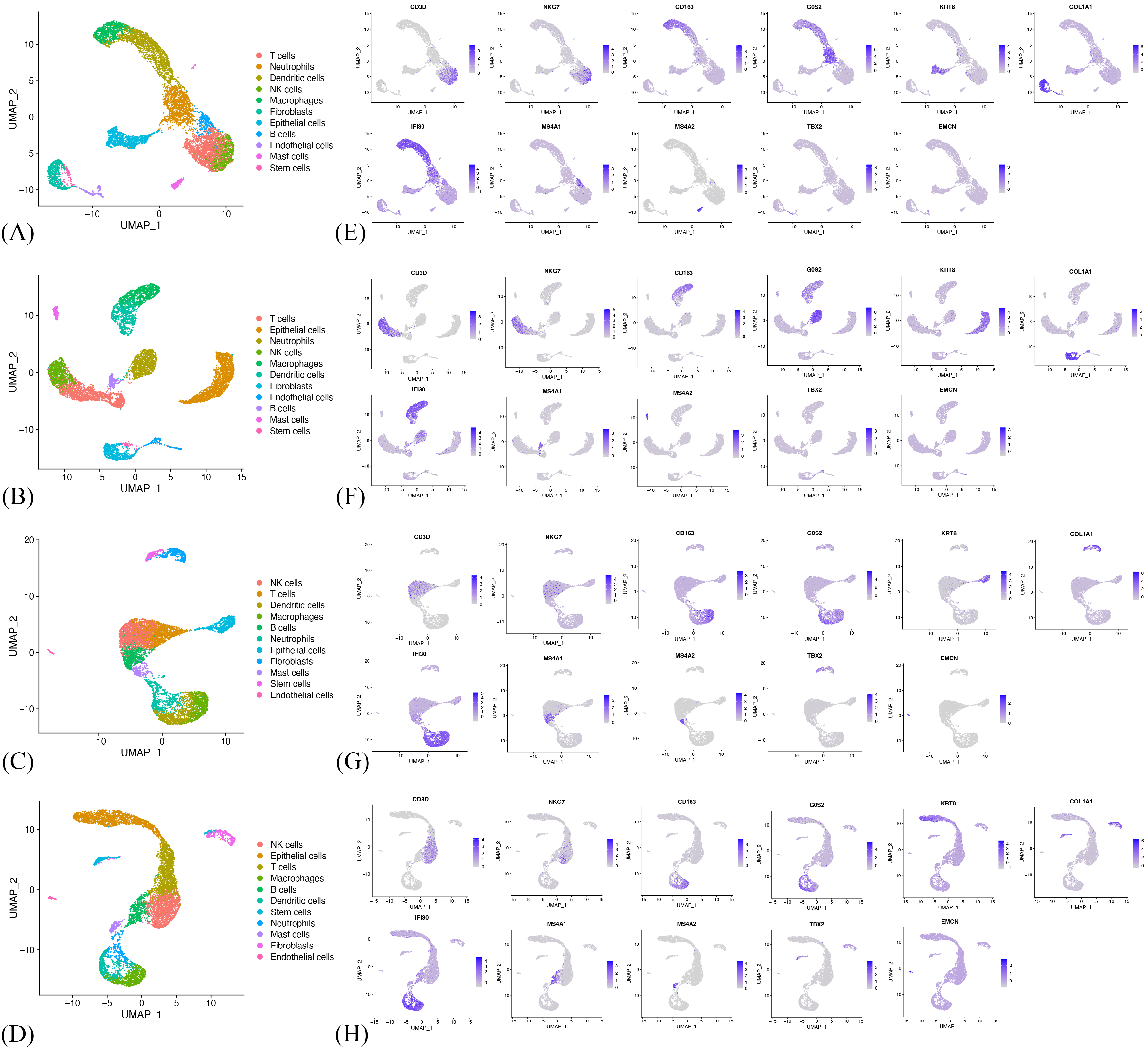

Supplement: Supplementary file 12 — Supporting information. Supplementary figure 12. Cell composition in the tumor microenvironment for each patient as evaluated by single‐cell RNA sequencing. The UMAP plots (A‐D) and canonical gene markers (E‐F) to label different cell clusters for each patient. [file CTM2-13-e1155-s012.jpg]

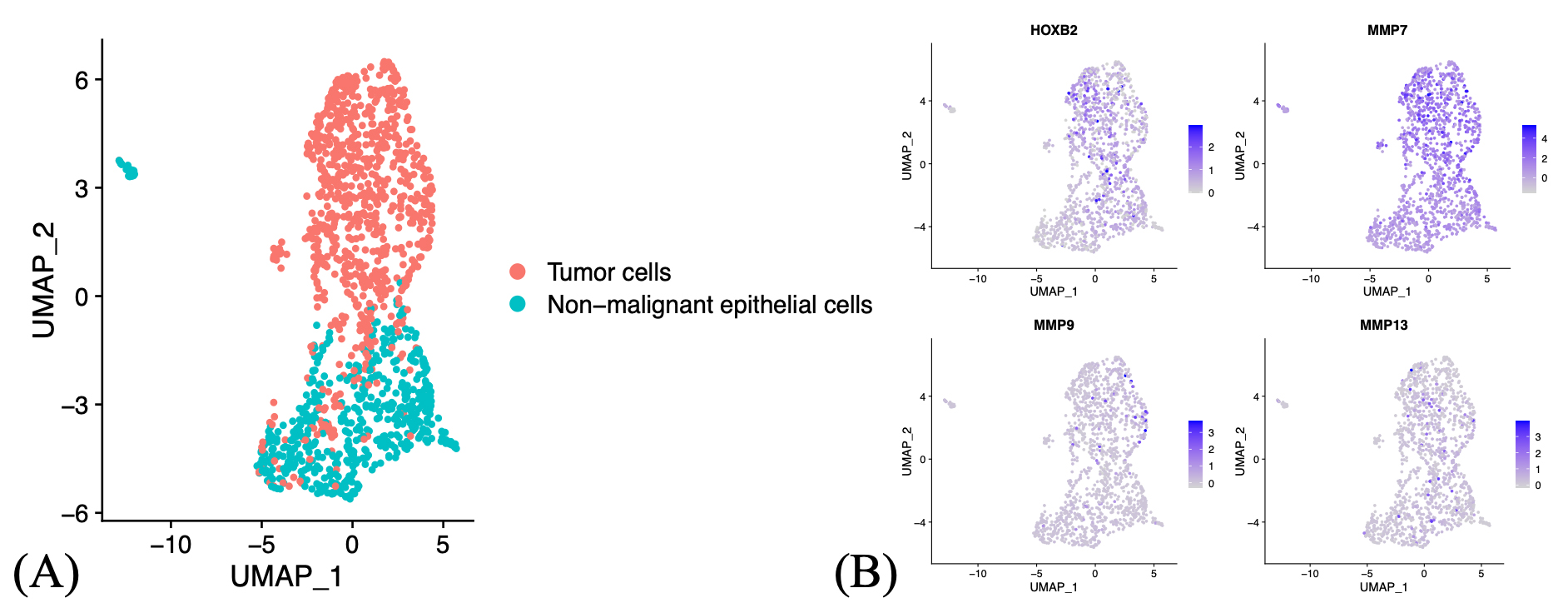

Supplement: Supplementary file 13 — Supporting information. Supplementary figure 13. Profiling the composition of epithelial cells through single‐cell RNA sequencing. Tumor cells and non‐malignant epithelial cells as represented in the UMAP plot (A). Canonical marker genes, including MMP7, MMP9, MMP13, and HOXB2, were used to label and distinguish between tumor cells and non‐malignant epithelial cells (B). [file CTM2-13-e1155-s007.jpg]

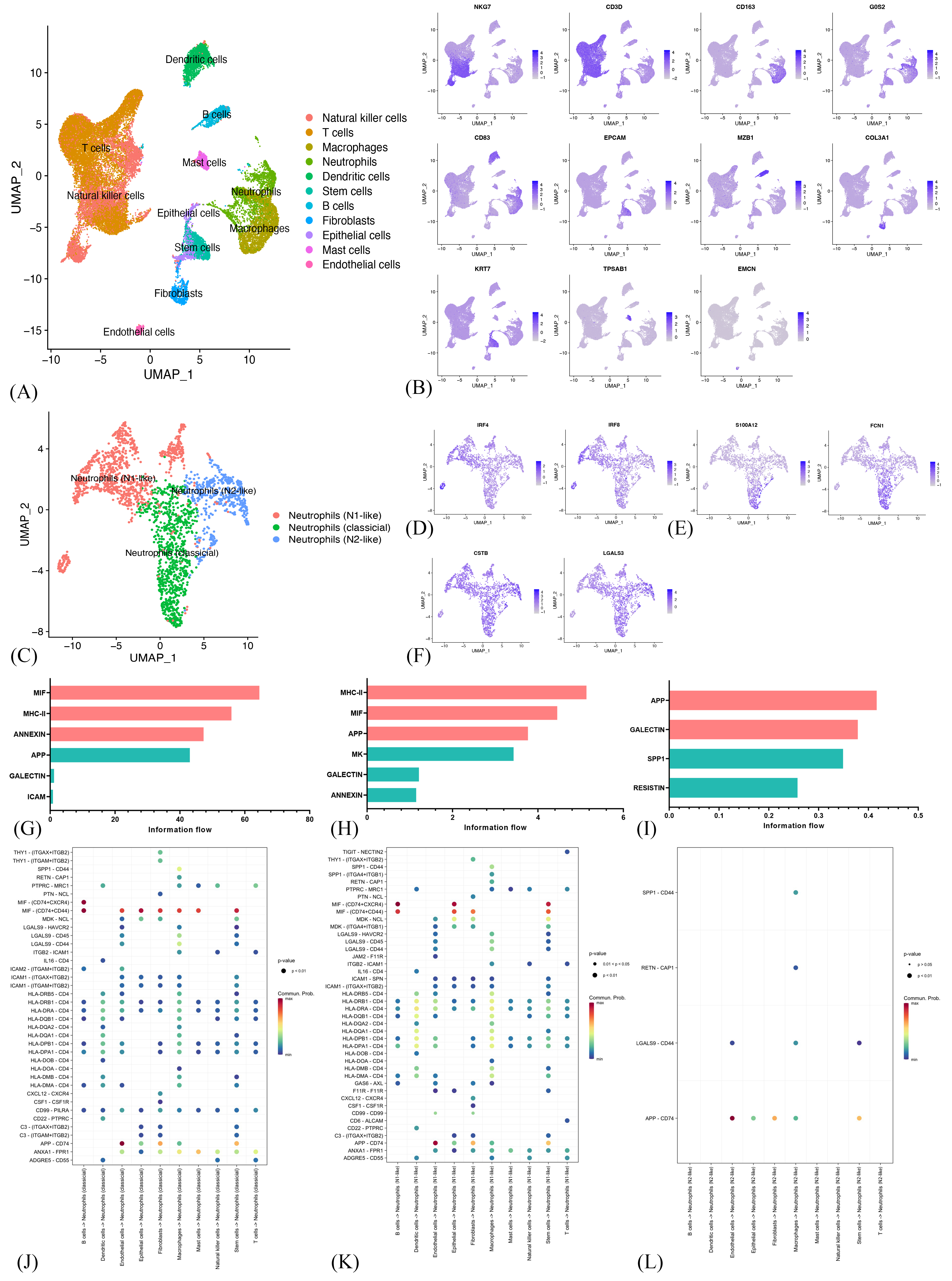

Supplement: Supplementary file 14 — Supporting information. Supplementary figure 14. External single‐cell RNA sequencing cohort of lung adenocarcinoma reveals cell composition and cell interaction networks in tumor microenvironment. Eleven major cell lineages as represented in the UMAP plot (A). Canonical gene markers to label clusters in the UMAP plot, including NKG7 for natural killer cells, CD3D for T cells, CD163 for macrophages, G0S2 for neutrophils, CD83 for dendritic cells, EPCAM for stem cells, MZB1 for B cells, COL3A1 for fibroblasts, KRT7 for epithelial cells, TPSAB1 for mast cells, and EMCN for endothelial cells (B). Classical, N1‐like, and N2‐like neutrophils as represented in the UMAP plot (C). Canonical gene markers to label three subclusters of neutrophils, including IRF4 and IRF8 for N1‐like neutrophils (D), S100A12 and FCN1 for classical neutrophils (E), and CSTB and LGALS3 for N2‐like neutrophils (F). The overall information flow of each signal pathway in classical (G), N1‐like (H), and N2‐like (I) neutrophils. Communication probabilities mediated by ligand‐receptor pairs with classical (J), N1‐like (K), and N2‐like (L) neutrophils as signal receivers. [file CTM2-13-e1155-s028.jpg]

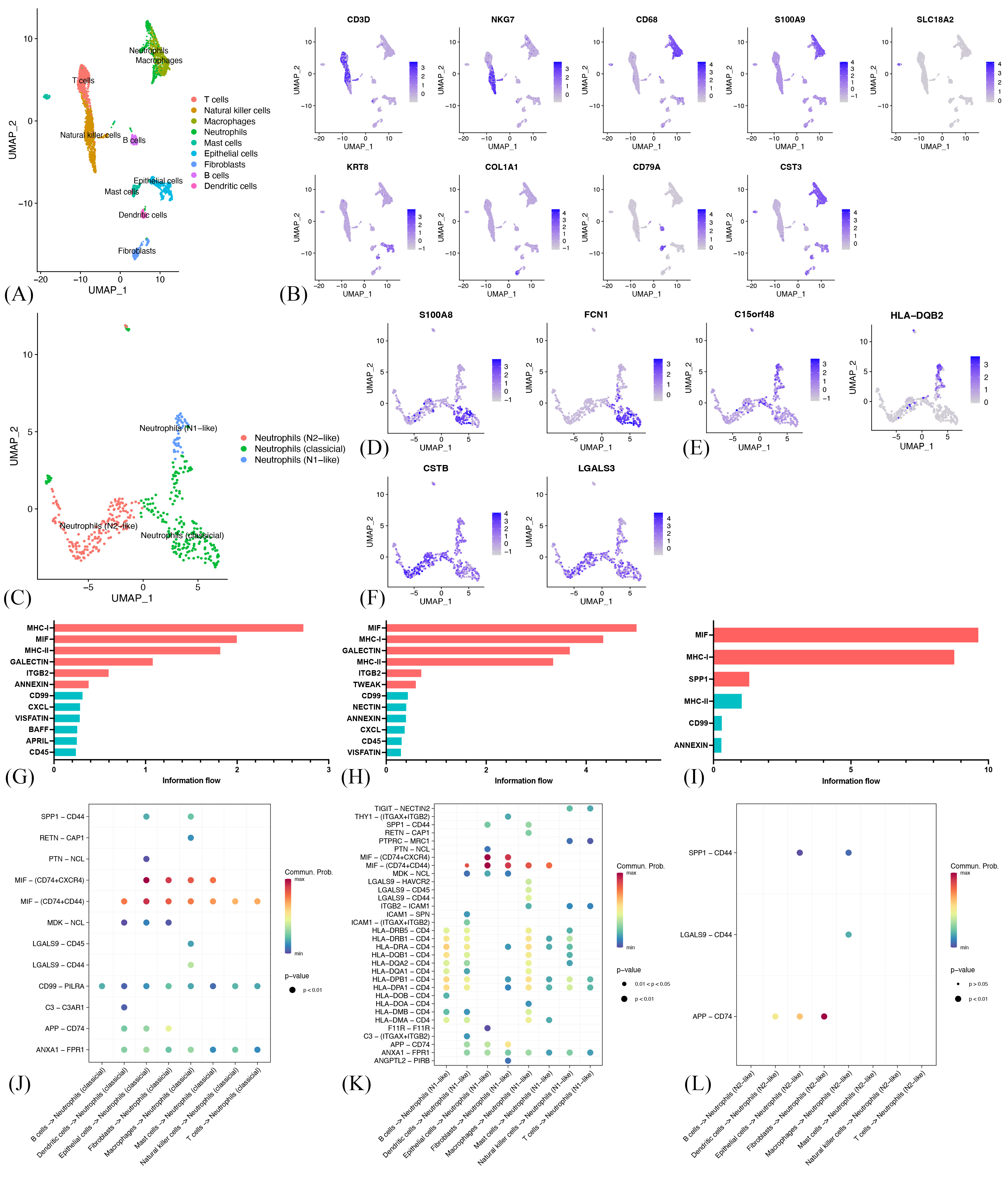

Supplement: Supplementary file 15 — Supporting information. Supplementary figure 15. External single‐cell RNA sequencing cohort of squamous cell lung cancer reveals cell composition and cell interaction networks in tumor microenvironment. Nine major cell lineages as represented in the UMAP plot (A). Canonical gene markers to label clusters in the UMAP plot, including CD3D for T cells, NKG7 for natural killer cells, CD68 for macrophages, S100A9 for neutrophils, SLC18A2 for mast cells, KRT8 for epithelial cells, COL1A1 for fibroblasts, CD79A for B cells, CST3 for dendritic cells (B). Classical, N1‐like, and N2‐like neutrophils as represented in the UMAP plot (C). Canonical gene markers to label three subsets of neutrophils, including S100A8 and FCN1 for classical neutrophils (D), C15orf48 and HLA‐DQB2 for N1‐like neutrophils (E), and CSTB and LGALS3 for N2‐like neutrophils (F). The overall information flow of each signal pathway in classical (G), N1‐like (H), and N2‐like (I) neutrophils. Communication probabilities mediated by ligand‐receptor pairs with classical (J), N1‐like (K), and N2‐like (L) neutrophils as signal receivers. [file CTM2-13-e1155-s026.jpg]

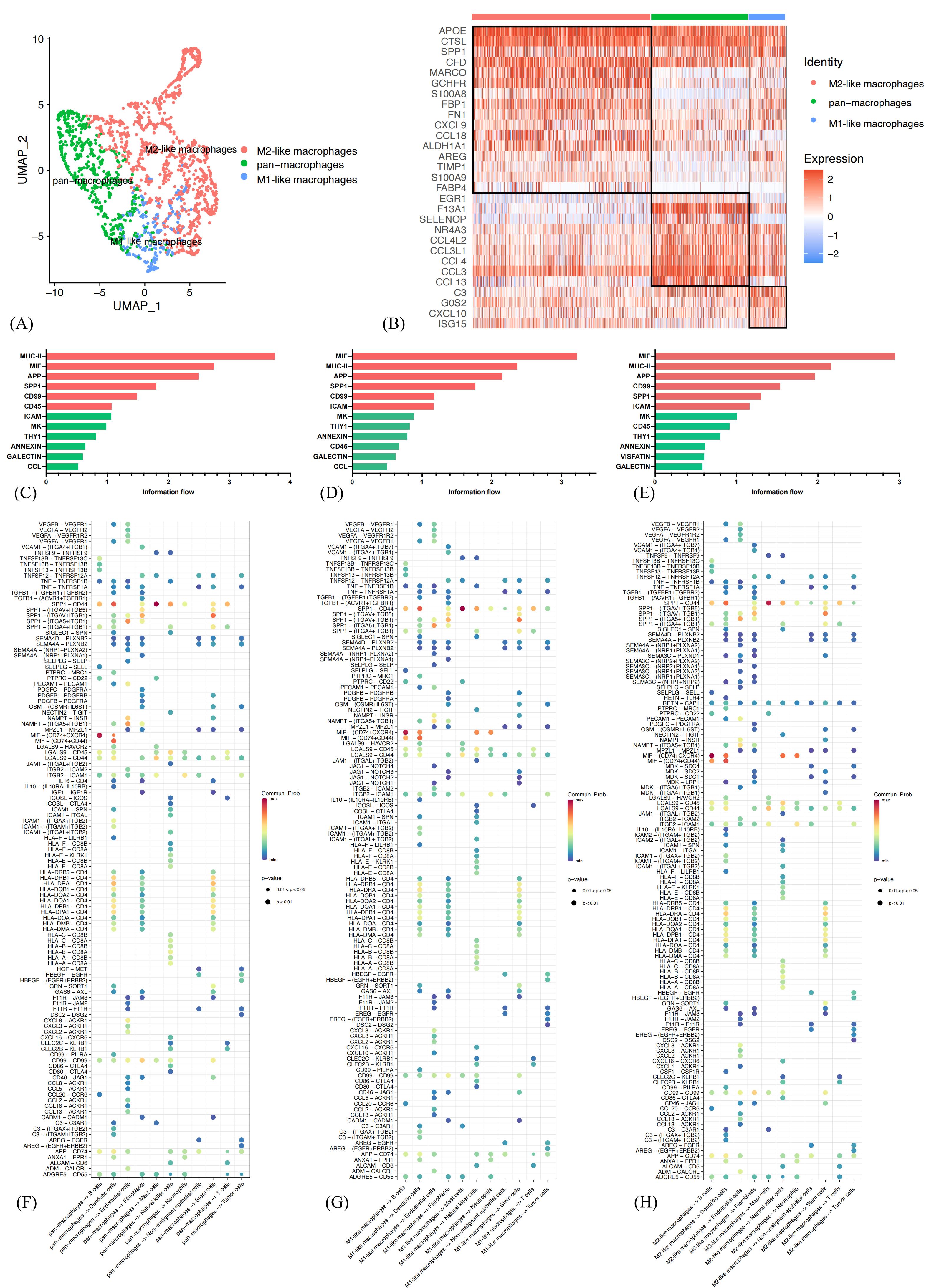

Supplement: Supplementary file 16 — Supporting information. Supplementary figure 16. Results of the CellChat analyses with macrophages as signal transmitters based on internal single‐cell RNA sequencing cohort of lung adenocarcinoma. UMAP plot (A) and heatmap of differentially expressed gene sets in macrophage subsets (B). The overall information flow of each signal pathway in pan‐macrophages (C), M1‐like (D), and M2‐like (E) macrophages. Communication probabilities mediated by ligand‐receptor pairs in pan‐macrophages (F), M1‐like (G), and M2‐like (H) macrophages. [file CTM2-13-e1155-s013.jpg]

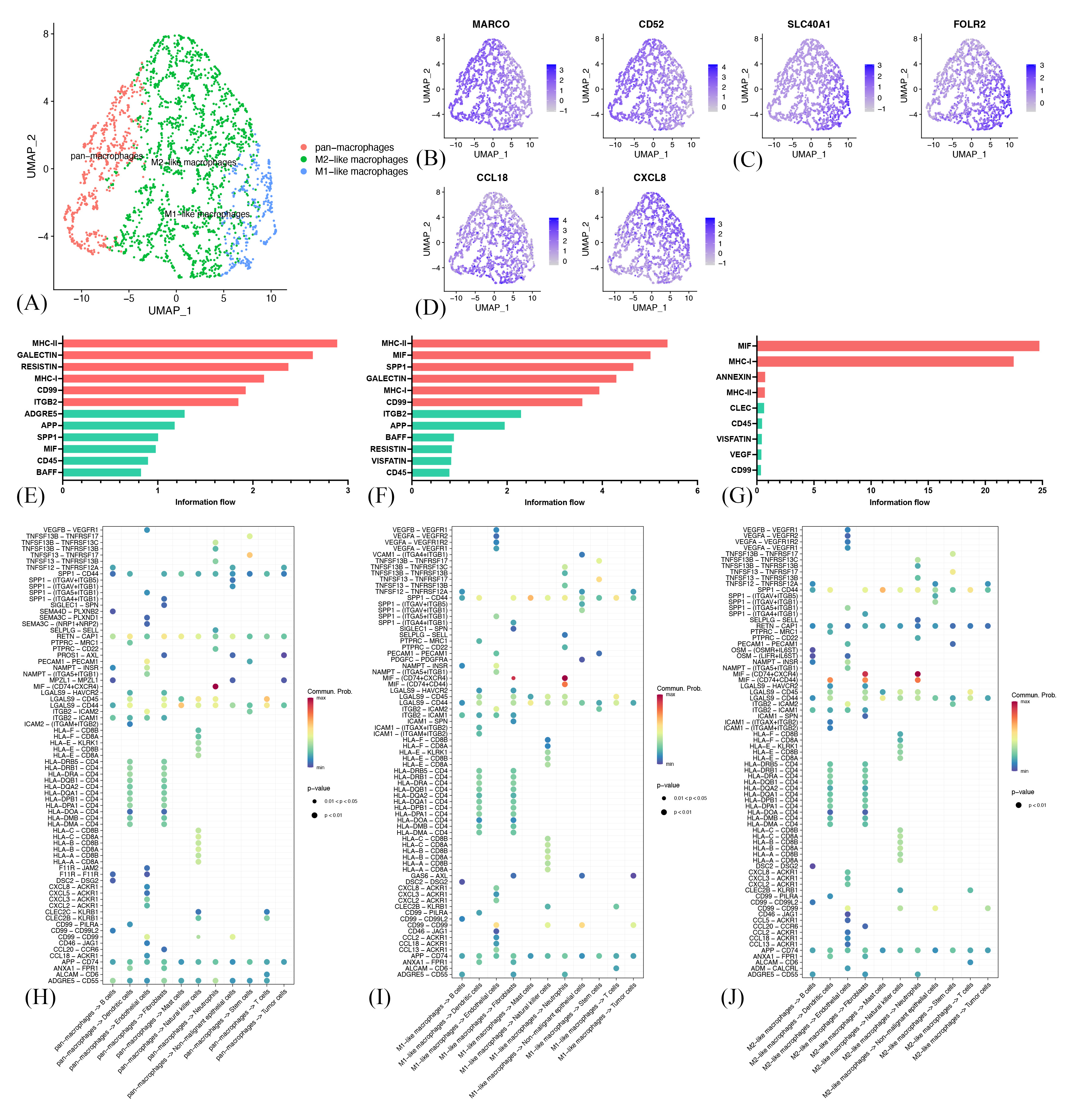

Supplement: Supplementary file 17 — Supporting information. Supplementary figure 17. Results of the CellChat analyses with macrophages as signal transmitters based on external single‐cell RNA sequencing cohort of lung adenocarcinoma. UMAP plot (A) and canonical gene markers to label three subsets of macrophages, including MARCO and CD52 for pan‐macrophages (B), SLC40A1 and FOLR2 for M1‐like macrophages (C), and CCL18 and CXCL8 for M2‐like macrophages (D). The overall information flow of each signal pathway in pan‐macrophages (E), M1‐like (F), and M2‐like (G) macrophages. Communication probabilities mediated by ligand‐receptor pairs in pan‐macrophages (H), M1‐like (I), and M2‐like (J) macrophages. [file CTM2-13-e1155-s005.jpg]

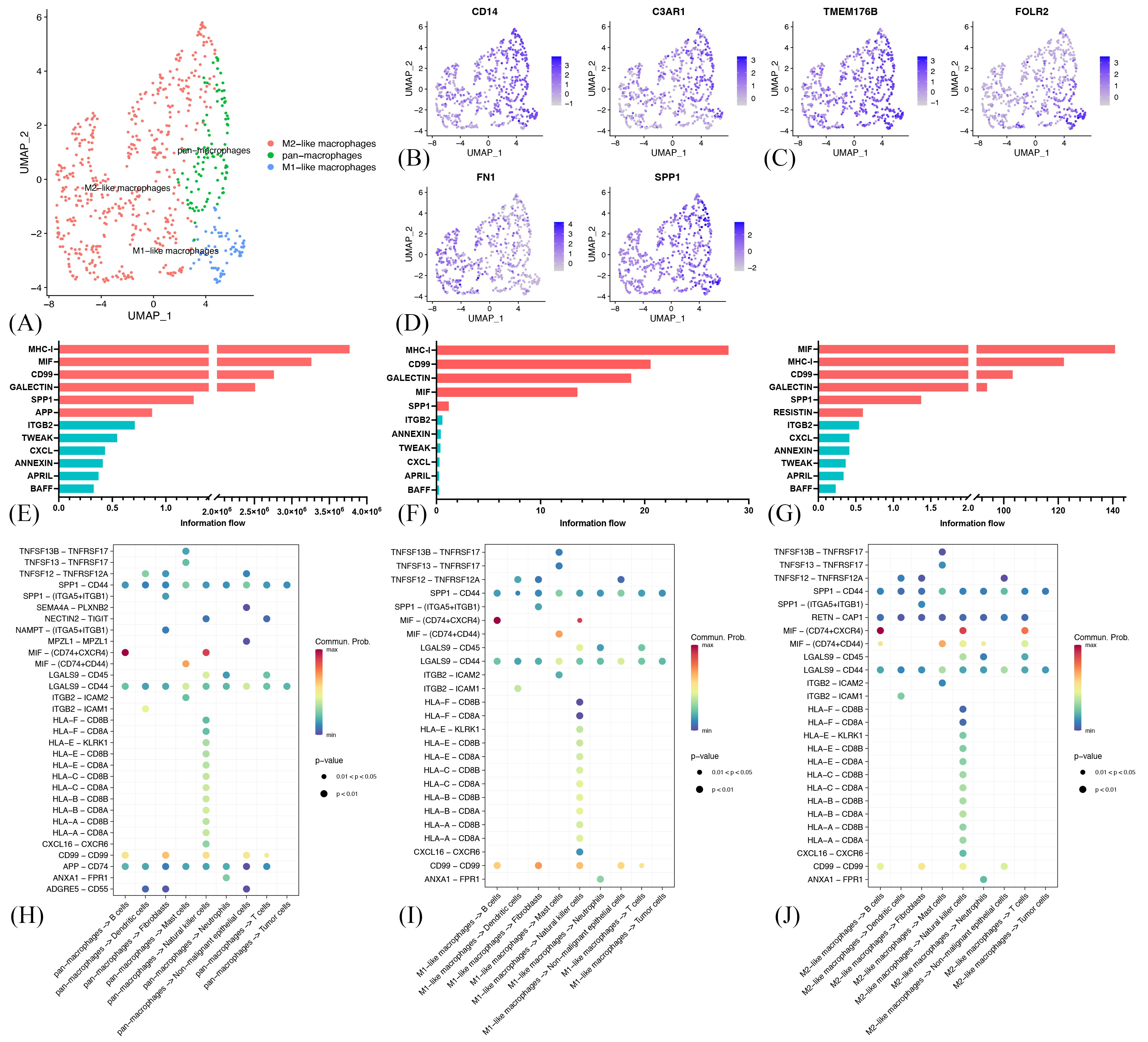

Supplement: Supplementary file 18 — Supporting information. Supplementary figure 18. Results of the CellChat analyses with macrophages as signal transmitters based on external single‐cell RNA sequencing cohort of squamous cell lung cancer. UMAP plot (A) and canonical gene markers to label three subsets of macrophages, including CD14 and C3AR1 for pan‐macrophages (B), TMEM176B and FOLR2 for M1‐like macrophages (C), and FN1 and SPP1 for M2‐like macrophages (D). The overall information flow of each signal pathway in pan‐macrophages (E), M1‐like (F), and M2‐like (G) macrophages. Communication probabilities mediated by ligand‐receptor pairs in pan‐macrophages (H), M1‐like (I), and M2‐like (J) macrophages. [file CTM2-13-e1155-s027.jpg]
