# Supplementary material for: Multiplex immunofluorescence and single‐cell transcriptomic profiling reveal the spatial cell interaction networks in the non‐small cell lung cancer microenvironment
Source: Clin Transl Med. 2023 Jan 1;13(1):e1155. doi: 10.1002/ctm2.1155 (PMC9806015; doi:10.1002/ctm2.1155)
Supplement: Supplementary file 19 — Supporting information. Supplementary table 1. Information of primary antibodies used in the multiplex immunofluorescence test. [file CTM2-13-e1155-s003.docx]

**Supplementary table 1.** Information of primary antibodies used in the multiplex immunofluorescence test.

| **Marker** | **Number** | **Clone ID** | **Company** | **Dilution** |
| --- | --- | --- | --- | --- |
| **CD4** | ZM0418 | UMAB64 | Zsbio | 1:200 |
| **CD20** | ab9475 | NA | abcam/Zsbio | 1:50/1:100 |
| **CD38** | ZM0422 | SPC32 | Zsbio | 1:400 |
| **PD-L1** | 13684s | E1L3N | CST | 1:100 |
| **CD66b** | ab214175 | NA | abcam | 1:50 |
| **CD8** | ZA-0508 | SP16 | Zsbio | 1:100 |
| **CD68** | ZM-0060 | KP1 | Zsbio | 1:100 |
| **CD163** | ZM-0428 | 10D6 | NA | NA |
| **FOXP3** | ab20034 | 236A/E7 | abcam | 1:100 |
| **CD133** | ab19898 | NA | abcam | 1:400 |

NA, no available.
