# Supplementary material for: Multiplex immunofluorescence and single‐cell transcriptomic profiling reveal the spatial cell interaction networks in the non‐small cell lung cancer microenvironment
Source: Clin Transl Med. 2023 Jan 1;13(1):e1155. doi: 10.1002/ctm2.1155 (PMC9806015; doi:10.1002/ctm2.1155)
Supplement: Supplementary file 20 — Supporting information. Supplementary table 2. Markers and corresponding cell types in multiplex immunofluorescence detection. [file CTM2-13-e1155-s004.docx]

**Supplementary table 2.** Markers and corresponding cell types in multiplex immunofluorescence detection.

| **Markers** | **Cell types** |
| --- | --- |
| CD4+ | CD4+ T cell |
| CD38+ | CD38 + T cell |
| CD66b+ | Neutrophil |
| FOXP3+ | FOXP3+ cell |
| CD20+ | CD20+ B cell |
| CD8+ | CD8+ T cell |
| PD-L1+ | PD-L1+ cell |
| CD163+ | CD163+ macrophage |
| CD68+ | CD68+ macrophage |
| CD133+ | CD133+ cell |
| CD4+ CD38+ | CD4+ CD38+ T cell |
| CD4- CD38+ | CD4- CD38+ T cell |
| CD4+ FOXP3+ | CD4+ Foxp3+ regulatory T cell |
| CD4+ FOXP3- | CD4+ FOXP3- T cell |
| CD8+ CD133+ | CD8+ T cell expressing CD133 |
| CD8+ CD133- | CD8+ T cell without expressing CD133 |
| CD68+ PD-L1- | CD68+ Macrophage without expressing PD-L1 |
| CD68+ PD-L1+ | CD68+ Macrophage expressing PD-L1 |
| CD163+ PD-L1- | CD163+ Macrophage without expressing PD-L1 |
| CD163+ PD-L1+ | CD163+ Macrophage expressing PD-L1 |
| CD68+ CD163+ | M2 Macrophage |
| CD68+ CD163- | M1 Macrophage |
| CD68+ CD163+ PD-L1+ | M2 Macrophage expressing PD-L1 |
| CD68+ CD163+ PD-L1- | M2 Macrophage without expressing PD-L1 |
| CD68+ CD163- PD-L1+ | M1 Macrophage expressing PD-L1 |
| CD68+ CD163- PD-L1- | M1 Macrophage without expressing PD-L1 |
