# Supplementary material for: Multiplex immunofluorescence and single‐cell transcriptomic profiling reveal the spatial cell interaction networks in the non‐small cell lung cancer microenvironment
Source: Clin Transl Med. 2023 Jan 1;13(1):e1155. doi: 10.1002/ctm2.1155 (PMC9806015; doi:10.1002/ctm2.1155)
Supplement: Supplementary file 21 — Supporting information. Supplementary table 3. Characteristics of the four non‐small cell lung cancer patients included in the single‐cell RNA sequencing. [file CTM2-13-e1155-s020.docx]

**Supplementary table 3.** Characteristics of the 4 non-small cell lung cancer patients included in the single-cell RNA sequencing.

| **Patient** | **Age** | **Gender** | **TNM** | **Stage** | **Histology** |
| --- | --- | --- | --- | --- | --- |
| 1 | 50 | Male | T3N2M0 | IIIB | Invasive Adenocarcinoma |
| 2 | 69 | Female | T2aN2M0 | IIIA | Invasive Adenocarcinoma |
| 3 | 52 | Male | T2bN1M0 | IIB | Invasive Adenocarcinoma |
| 4 | 62 | Male | T2aN1M0 | IIB | Invasive Adenocarcinoma |
