# Supplementary material for: Multiplex immunofluorescence and single‐cell transcriptomic profiling reveal the spatial cell interaction networks in the non‐small cell lung cancer microenvironment
Source: Clin Transl Med. 2023 Jan 1;13(1):e1155. doi: 10.1002/ctm2.1155 (PMC9806015; doi:10.1002/ctm2.1155)
Supplement: Supplementary file 23 — Supporting information. Supplementary table 5. Demographic characteristics and immune‐related risk score of the included patients. [file CTM2-13-e1155-s015.docx]

**Supplementary table 5.** Demographic characteristics and immune-related risk score of included patients.

| **Patient ID** | **Disease free survival (days)** | **Disease free survival status** | **Sex** | **Age** | **Clinical stage** | **IRRS*** |
| --- | --- | --- | --- | --- | --- | --- |
| 1 | 696 | Reccurence | Male | 65 | ⅢB | 0.998663594 |
| 2 | 2378 | Non-reccurence | Female | 68 | ⅠB | 0.742815856 |
| 3 | 1814 | Non-reccurence | Male | 82 | ⅠA | 1.075928152 |
| 4 | 225 | Reccurence | Female | 65 | ⅠA | 1.386266287 |
| 5 | 595 | Reccurence | Female | 76 | ⅠA | 1.024123425 |
| 6 | 1364 | Non-reccurence | Male | 65 | ⅡA | 1.615807004 |
| 7 | 2255 | Non-reccurence | Female | 55 | ⅠB | 0.896432143 |
| 8 | 2274 | Non-reccurence | Female | 58 | ⅠB | -0.02479798 |
| 9 | 467 | Reccurence | Male | 56 | ⅡB | -0.25574939 |
| 10 | 1845 | Reccurence | Male | 62 | ⅠB | -0.05793837 |
| 11 | 1148 | Reccurence | Male | 47 | ⅢB | 0.038613339 |
| 12 | 218 | Non-reccurence | Female | 46 | ⅠB | 0.683779769 |
| 13 | 2414 | Non-reccurence | Male | 65 | ⅢB | -0.03863814 |
| 14 | 2407 | Non-reccurence | Female | 56 | ⅡB | -0.66802349 |
| 15 | 363 | Reccurence | Female | 70 | ⅠB | 0.692443776 |
| 16 | 2408 | Non-reccurence | Male | 56 | ⅡB | -0.89840542 |
| 17 | 1100 | Reccurence | Male | 67 | ⅡA | 0.843678055 |
| 18 | 2406 | Non-reccurence | Female | 31 | ⅡB | #N/A |
| 19 | 2400 | Non-reccurence | Female | 45 | ⅠB | -0.09009517 |
| 20 | 573 | Reccurence | Male | 77 | ⅢB | 0.170218108 |
| 21 | 185 | Reccurence | Female | 32 | ⅡA | -0.01307344 |
| 22 | 840 | Reccurence | Male | 62 | ⅢA | 1.325531809 |
| 23 | 2279 | Non-reccurence | Female | 64 | ⅡA | 0.397243728 |
| 24 | 857 | Reccurence | Male | 77 | ⅡB | 0.398380712 |
| 25 | 2552 | Non-reccurence | Female | 60 | ⅡA | 0.36985247 |
| 26 | 2265 | Non-reccurence | Female | 72 | ⅡA | #N/A |
| 27 | 2271 | Non-reccurence | Male | 53 | ⅡB | 0.015854851 |
| 28 | 2239 | Non-reccurence | Male | 65 | ⅡA | -0.22310315 |
| 29 | 2386 | Non-reccurence | Male | 55 | ⅢA | 0.362761998 |
| 30 | 2351 | Non-reccurence | Female | 46 | ⅡA | 0.891564744 |
| 31 | 2378 | Non-reccurence | Female | 70 | ⅠB | #N/A |
| 32 | 2379 | Non-reccurence | Male | 53 | ⅢA | -0.31651417 |
| 33 | 1782 | Reccurence | Female | 71 | ⅠA | 0.72153333 |
| 34 | 2385 | Non-reccurence | Male | 47 | ⅡA | -0.05358055 |
| 35 | 2378 | Non-reccurence | Male | 49 | ⅠB | 0.811425339 |
| 36 | 2381 | Non-reccurence | Male | 65 | ⅠB | 0.104215647 |
| 37 | 2249 | Non-reccurence | Female | 42 | ⅡA | 0.89080207 |
| 38 | 232 | Reccurence | Male | 52 | ⅢB | -0.93579472 |
| 39 | 2254 | Non-reccurence | Male | 53 | ⅡB | 0.619056997 |
| 40 | 2244 | Non-reccurence | Female | 51 | ⅡA | 1.176584344 |
| 41 | 502 | Reccurence | Female | 61 | ⅢB | 0.831280756 |
| 42 | 2259 | Non-reccurence | Male | 54 | ⅢB | 0.562009363 |
| 43 | 581 | Reccurence | Female | 43 | ⅠA | 0.896960439 |
| 44 | 2252 | Non-reccurence | Female | 75 | ⅡA | -0.60447021 |
| 45 | 2270 | Non-reccurence | Male | 56 | ⅢB | -0.30916362 |
| 46 | 1248 | Reccurence | Male | 69 | ⅠA | 1.625958044 |
| 47 | 481 | Reccurence | Male | 41 | ⅢB | 0.521930265 |
| 48 | 2263 | Non-reccurence | Male | 69 | ⅡA | #N/A |
| 49 | 77 | Reccurence | Male | 56 | ⅡB | -0.11385599 |
| 50 | 2246 | Non-reccurence | Female | 58 | ⅡA | 0.089656699 |
| 51 | 1372 | Reccurence | Male | 64 | ⅡA | #N/A |
| 52 | 2192 | Reccurence | Female | 72 | ⅠB | 0.440867192 |
| 53 | 2240 | Non-reccurence | Female | 19 | ⅡA | 0.329925552 |
| 54 | 161 | Reccurence | Female | 55 | ⅢA | 0.836640327 |
| 55 | 2230 | Reccurence | Male | 66 | ⅡB | -0.26046034 |
| 56 | 1390 | Reccurence | Male | 62 | ⅢB | #N/A |
| 57 | 1876 | Reccurence | Male | 75 | ⅢA | -2.12629687 |
| 58 | 1800 | Reccurence | Male | 72 | ⅢB | -0.34171646 |
| 59 | 2222 | Non-reccurence | Male | 57 | ⅡA | #N/A |
| 60 | 1821 | Reccurence | Male | 56 | ⅢB | -1.68197872 |
| 61 | 1517 | Non-reccurence | Female | 38 | ⅠB | 0.350053393 |
| 62 | 2230 | Non-reccurence | Male | 45 | ⅢB | 0.201389029 |
| 63 | 2215 | Non-reccurence | Female | 69 | ⅡA | 0.231760738 |
| 64 | 1899 | Non-reccurence | Male | 44 | ⅢB | 0.049998098 |
| 65 | 2200 | Non-reccurence | Male | 62 | ⅡA | 1.073745049 |
| 66 | 1234 | Reccurence | Male | 59 | ⅡA | -0.78006966 |
| 67 | 1736 | Reccurence | Male | 51 | ⅡB | 0.183000041 |
| 68 | 2229 | Non-reccurence | Female | 58 | ⅡA | 0.670669337 |
| 69 | 2333 | Non-reccurence | Male | 66 | ⅠA | 1.955684514 |
| 70 | 2341 | Non-reccurence | Male | 73 | ⅡA | 1.097276596 |
| 71 | 2269 | Non-reccurence | Female | 53 | ⅡA | -2.22380909 |
| 72 | 2221 | Non-reccurence | Male | 41 | ⅠB | 1.217659507 |
| 73 | 1400 | Reccurence | Male | 59 | ⅢB | -1.36593158 |
| 74 | 2203 | Non-reccurence | Male | 48 | ⅡA | 1.085454532 |
| 75 | 2309 | Non-reccurence | Female | 44 | ⅡA | 0.348945163 |
| 76 | 696 | Reccurence | Female | 44 | ⅠB | 1.553962996 |
| 77 | 2190 | Non-reccurence | Male | 64 | ⅡA | -0.36728785 |
| 78 | 1361 | Reccurence | Male | 75 | ⅠA | 0.280433613 |
| 79 | 608 | Reccurence | Female | 56 | ⅢB | 0.18966264 |
| 80 | 1492 | Non-reccurence | Female | 45 | ⅠB | 0.71609636 |
| 81 | 144 | Reccurence | Female | 79 | ⅢB | 0.820623086 |
| 82 | 102 | Reccurence | Male | 51 | ⅡB | 0.052520746 |
| 83 | 587 | Reccurence | Female | 54 | ⅢB | 1.105631154 |
| 84 | 2198 | Non-reccurence | Male | 63 | ⅠB | 0.773317704 |
| 85 | 1166 | Reccurence | Male | 73 | ⅠB | 0.49162438 |
| 86 | 2307 | Non-reccurence | Female | 54 | ⅡA | 0.469718838 |
| 87 | 1988 | Reccurence | Female | 66 | ⅠB | 0.975275422 |
| 88 | 2206 | Non-reccurence | Male | 47 | ⅠB | 0.555661848 |
| 89 | 2312 | Non-reccurence | Female | 57 | ⅠB | #N/A |
| 90 | 687 | Reccurence | Male | 66 | ⅡB | -0.67729236 |
| 91 | 619 | Reccurence | Female | 64 | ⅢB | 0.305218072 |
| 92 | 1394 | Reccurence | Male | 55 | ⅡB | 0.308424013 |
| 93 | 103 | Reccurence | Male | 64 | ⅢB | #N/A |
| 94 | 176 | Reccurence | Male | 62 | ⅢB | 1.566920581 |
| 95 | 1667 | Reccurence | Male | 52 | ⅢA | 0.753243634 |
| 96 | 1913 | Reccurence | Male | 70 | ⅡA | 1.316880523 |
| 97 | 2300 | Non-reccurence | Male | 59 | ⅡA | 0.69060639 |
| 98 | 2192 | Non-reccurence | Male | 40 | ⅡA | 0.393945813 |
| 99 | 695 | Reccurence | Male | 72 | ⅡA | 1.610169769 |
| 100 | 368 | Reccurence | Female | 55 | ⅠB | 1.018926964 |
| 101 | 769 | Reccurence | Male | 74 | ⅠB | #N/A |
| 102 | 90 | Reccurence | Female | 47 | ⅢB | 1.051303956 |
| 103 | 2184 | Non-reccurence | Male | 69 | ⅡA | -0.08130718 |
| 104 | 619 | Reccurence | Male | 65 | ⅡA | 0.385788222 |
| 105 | 1456 | Non-reccurence | Female | 26 | ⅠB | 0.349330709 |
| 106 | 2179 | Non-reccurence | Male | 61 | ⅡA | 0.600754446 |
| 107 | 2179 | Non-reccurence | Female | 56 | ⅡA | 0.61790632 |
| 108 | 1792 | Reccurence | Male | 62 | ⅢB | 0.131879393 |
| 109 | 2270 | Non-reccurence | Male | 54 | ⅡB | -3.01600397 |
| 110 | 2273 | Non-reccurence | Female | 69 | ⅢB | 0.057982487 |
| 111 | 1429 | Reccurence | Male | 62 | ⅡB | #N/A |
| 112 | 2269 | Non-reccurence | Female | 39 | ⅠB | 1.341630361 |
| 113 | 2253 | Non-reccurence | Male | 75 | ⅡA | -0.73760779 |
| 114 | 1163 | Reccurence | Female | 56 | ⅡA | 0.162874214 |
| 115 | 2350 | Reccurence | Male | 55 | ⅠB | 0.656653667 |
| 116 | 977 | Reccurence | Male | 41 | ⅢB | 0.547809967 |
| 117 | 1092 | Reccurence | Male | 52 | ⅡA | 0.378794611 |
| 118 | 2078 | Reccurence | Female | 49 | ⅢB | 1.303420164 |
| 119 | 436 | Reccurence | Male | 50 | ⅡA | #N/A |
| 120 | 2489 | Reccurence | Female | 61 | ⅡA | -0.48416335 |
| 121 | 2241 | Non-reccurence | Female | 72 | ⅡA | #N/A |
| 122 | 2241 | Non-reccurence | Female | 61 | ⅡA | #N/A |
| 123 | 1184 | Reccurence | Male | 76 | ⅢB | #N/A |
| 124 | 497 | Reccurence | Female | 69 | ⅢB | 0.787612703 |
| 125 | 2227 | Non-reccurence | Female | 45 | ⅡB | -1.95802339 |
| 126 | 1819 | Reccurence | Female | 70 | ⅠB | 0.494349817 |
| 127 | 2268 | Reccurence | Female | 55 | ⅡB | 1.472006674 |
| 128 | 2231 | Non-reccurence | Male | 55 | ⅡA | 0.681685392 |
| 129 | 105 | Reccurence | Male | 63 | ⅢB | 0.642331783 |
| 130 | 175 | Reccurence | Male | 67 | ⅢB | 1.081966898 |
| 131 | 176 | Reccurence | Male | 85 | ⅢB | 0.428952222 |
| 132 | 2210 | Non-reccurence | Male | 59 | ⅡA | 0.130706658 |
| 133 | 2219 | Non-reccurence | Male | 54 | ⅡA | 0.62297544 |
| 134 | 2224 | Non-reccurence | Male | 67 | ⅡB | 0.465391589 |
| 135 | 2216 | Non-reccurence | Male | 81 | ⅠA | #N/A |
| 136 | 2093 | Non-reccurence | Male | 70 | ⅡB | -4.1343791 |
| 137 | 812 | Reccurence | Female | 56 | ⅡB | 0.401392768 |
| 138 | 30 | Reccurence | Female | 60 | ⅢB | 0.619779695 |
| 139 | 180 | Reccurence | Male | 61 | ⅡB | 1.368813714 |
| 140 | 2095 | Non-reccurence | Male | 56 | ⅡB | 0.890202851 |
| 141 | 2088 | Non-reccurence | Male | 65 | ⅢB | #N/A |
| 142 | 1082 | Reccurence | Male | 60 | ⅡA | #N/A |
| 143 | 2209 | Non-reccurence | Male | 65 | ⅡA | #N/A |
| 144 | 2074 | Non-reccurence | Male | 56 | ⅠB | #N/A |
| 145 | 254 | Reccurence | Male | 58 | ⅡB | 0.489179966 |
| 146 | 2209 | Non-reccurence | Male | 56 | ⅠB | -0.74727292 |
| 147 | 218 | Reccurence | Male | 57 | ⅡB | 1.556156098 |
| 148 | 1402 | Reccurence | Female | 61 | ⅢB | 0.553025327 |
| 149 | 707 | Reccurence | Female | 61 | ⅢB | #N/A |
| 150 | 2207 | Non-reccurence | Female | 44 | ⅡA | 0.12840785 |
| 151 | 2198 | Non-reccurence | Male | 56 | ⅡB | 0.494971864 |
| 152 | 425 | Reccurence | Male | 47 | ⅠA | 0.679797855 |
| 153 | 342 | Reccurence | Female | 42 | ⅡB | 1.019611217 |
| 154 | 1016 | Reccurence | Female | 48 | ⅡA | 0.54884547 |
| 155 | 673 | Reccurence | Female | 77 | ⅠB | 0.884480167 |
| 156 | 252 | Reccurence | Female | 50 | ⅢB | 1.07756232 |
| 157 | 1031 | Reccurence | Male | 69 | ⅢB | 0.647005478 |
| 158 | 584 | Reccurence | Male | 64 | ⅡB | #N/A |
| 159 | 436 | Reccurence | Male | 60 | ⅡB | 0.966055036 |
| 160 | 2025 | Non-reccurence | Female | 72 | ⅠA | 0.623000491 |
| 161 | 728 | Reccurence | Female | 60 | ⅡB | 0.97605108 |
| 162 | 1599 | Reccurence | Male | 68 | ⅠB | 1.216883099 |
| 163 | 2045 | Non-reccurence | Female | 74 | ⅡA | #N/A |
| 164 | 666 | Reccurence | Male | 58 | ⅡB | 0.792552946 |
| 165 | 1537 | Reccurence | Male | 60 | ⅡA | 0.985545617 |
| 166 | 2028 | Non-reccurence | Female | 56 | ⅢB | 0.073611982 |
| 167 | 2041 | Non-reccurence | Female | 54 | ⅡA | 0.208201007 |
| 168 | 2171 | Non-reccurence | Male | 56 | ⅡB | #N/A |
| 169 | 1998 | Non-reccurence | Female | 63 | ⅡA | -0.27328953 |
| 170 | 1850 | Reccurence | Female | 79 | ⅡB | #N/A |
| 171 | 558 | Reccurence | Male | 85 | ⅡA | 2.002240495 |
| 172 | 2029 | Non-reccurence | Female | 66 | ⅡB | 0.683195885 |
| 173 | 1063 | Reccurence | Male | 73 | ⅡB | 0.881150907 |
| 174 | 803 | Reccurence | Female | 58 | ⅢB | 1.272507413 |
| 175 | 2054 | Non-reccurence | Female | 34 | ⅠB | #N/A |
| 176 | 2014 | Non-reccurence | Female | 45 | ⅢB | 0.810397489 |
| 177 | 107 | Reccurence | Female | 63 | 6 | 0.364909889 |
| 178 | 2038 | Non-reccurence | Male | 65 | ⅢB | 0.619973795 |
| 179 | 2030 | Non-reccurence | Female | 72 | ⅡA | 0.679816551 |
| 180 | 2578 | Reccurence | Male | 79 | ⅢB | 0.015079798 |
| 181 | 2023 | Non-reccurence | Female | 49 | ⅠB | 0.489385339 |
| 182 | 2035 | Non-reccurence | Female | 58 | ⅠB | #N/A |
| 183 | 233 | Reccurence | Male | 60 | ⅡA | 0.674650099 |
| 184 | 2138 | Non-reccurence | Female | 71 | ⅡA | 0.846108315 |
| 185 | 177 | Reccurence | Male | 64 | ⅢB | -1.80342027 |
| 186 | 2040 | Non-reccurence | Female | 51 | ⅢB | 0.758241379 |
| 187 | 345 | Reccurence | Female | 50 | ⅢB | 0.824766447 |
| 188 | 2007 | Non-reccurence | Male | 49 | ⅠB | 0.418810796 |
| 189 | 2287 | Reccurence | Female | 44 | ⅡA | -0.18842875 |
| 190 | 1186 | Reccurence | Male | 57 | ⅢB | #N/A |
| 191 | 224 | Reccurence | Male | 61 | ⅠA | 0.3933511 |
| 192 | 2014 | Non-reccurence | Male | 76 | ⅡA | 0.676418362 |
| 193 | 2077 | Non-reccurence | Male | 61 | ⅠB | #N/A |
| 194 | 2017 | Non-reccurence | Male | 55 | ⅠB | #N/A |
| 195 | 2015 | Non-reccurence | Male | 46 | ⅡA | 0.536972761 |
| 196 | 2026 | Non-reccurence | Male | 70 | ⅢB | 0.468752517 |
| 197 | 2017 | Non-reccurence | Male | 64 | ⅡA | 0.996661663 |
| 198 | 378 | Reccurence | Male | 75 | 6 | 1.176256901 |
| 199 | 479 | Reccurence | Female | 76 | ⅢB | 0.739093457 |
| 200 | 1364 | Reccurence | Male | 62 | ⅢB | -0.06606489 |
| 201 | 507 | Reccurence | Female | 69 | ⅢB | 0.419820159 |
| 202 | 151 | Reccurence | Male | 62 | ⅢA | #N/A |
| 203 | 2014 | Non-reccurence | Male | 54 | ⅡA | #N/A |
| 204 | 49 | Reccurence | Female | 57 | ⅢB | 1.154033765 |
| 205 | 2014 | Non-reccurence | Female | 57 | ⅡA | 0.713888243 |
| 206 | 2013 | Non-reccurence | Female | 51 | ⅡA | #N/A |
| 207 | 1291 | Non-reccurence | Female | 54 | ⅡB | -3.61934014 |
| 208 | 2065 | Non-reccurence | Male | 63 | ⅡB | #N/A |
| 209 | 2010 | Non-reccurence | Female | 57 | ⅠB | 1.675784306 |
| 210 | 2009 | Reccurence | Male | 57 | ⅠA | -0.44440096 |
| 211 | 2109 | Non-reccurence | Male | 39 | ⅢA | 0.070548235 |
| 212 | 2007 | Non-reccurence | Female | 72 | ⅡB | #N/A |
| 213 | 439 | Reccurence | Male | 58 | ⅢB | 0.599903498 |
| 214 | 444 | Reccurence | Male | 75 | ⅢB | 0.656958864 |
| 215 | 2091 | Non-reccurence | Male | 53 | ⅢB | 0.382852817 |
| 216 | 1748 | Reccurence | Male | 58 | ⅡA | 1.019609964 |
| 217 | 1462 | Reccurence | Male | 68 | ⅡA | -0.18802666 |
| 218 | 882 | Reccurence | Male | 56 | ⅡA | 0.915321923 |
| 219 | 1997 | Non-reccurence | Female | 69 | ⅠA | 0.595914191 |
| 220 | 2089 | Non-reccurence | Female | 38 | ⅡA | 0.678880085 |
| 221 | 704 | Reccurence | Male | 56 | ⅢB | 0.616408995 |
| 222 | 2097 | Non-reccurence | Female | 56 | ⅡA | 0.33158972 |
| 223 | 163 | Reccurence | Male | 56 | ⅢB | 0.485056754 |
| 224 | 2098 | Non-reccurence | Female | 48 | ⅢB | 1.537551138 |
| 225 | 186 | Reccurence | Male | 78 | ⅢB | 0.428637875 |
| 226 | 2081 | Non-reccurence | Female | 57 | ⅡA | 0.261367545 |
| 227 | 2092 | Non-reccurence | Male | 60 | ⅡA | 0.742695619 |
| 228 | 409 | Reccurence | Female | 68 | ⅢB | 0.860325966 |
| 229 | 2080 | Non-reccurence | Female | 76 | ⅡA | 1.080504192 |
| 230 | 2082 | Non-reccurence | Female | 59 | ⅡA | 0.162244083 |
| 231 | 2091 | Non-reccurence | Male | 63 | ⅡA | #N/A |
| 232 | 2084 | Non-reccurence | Female | 66 | ⅡA | -0.17828578 |
| 233 | 150 | Reccurence | Female | 58 | ⅡB | 0.51105695 |
| 234 | 899 | Reccurence | Male | 61 | ⅢB | 1.419422246 |
| 235 | 730 | Reccurence | Male | 71 | ⅢB | -0.57082956 |
| 236 | 1257 | Reccurence | Female | 60 | ⅢB | 1.007636195 |
| 237 | 2079 | Non-reccurence | Male | 65 | ⅠB | 0.890990813 |
| 238 | 2063 | Non-reccurence | Male | 63 | ⅡB | 0.926563556 |
| 239 | 2071 | Non-reccurence | Female | 61 | ⅡB | 1.068184879 |
| 240 | 1201 | Reccurence | Male | 82 | ⅢB | 1.252887817 |
| 241 | 1948 | Non-reccurence | Male | 62 | ⅠB | 0.11367327 |
| 242 | 2431 | Non-reccurence | Male | 75 | ⅠB | 0.263817392 |
| 243 | 1924 | Non-reccurence | Female | 53 | ⅠB | -0.30404461 |
| 244 | 1022 | Reccurence | Male | 54 | ⅠA | 1.058196946 |
| 245 | 1763 | Non-reccurence | Female | 20 | ⅡA | 1.167629149 |
| 246 | 2054 | Non-reccurence | Female | 59 | ⅠB | 0.252426657 |
| 247 | 402 | Reccurence | Male | 66 | ⅢB | 0.349732575 |
| 248 | 612 | Reccurence | Male | 52 | ⅡB | #N/A |
| 249 | 1828 | Reccurence | Male | 72 | ⅡA | 0.676409552 |
| 250 | 2052 | Non-reccurence | Male | 68 | ⅡA | 0.291863998 |
| 251 | 2049 | Non-reccurence | Male | 58 | ⅠB | 0.745223442 |
| 252 | 2055 | Non-reccurence | Male | 72 | ⅠB | 0.052256205 |
| 253 | 2044 | Non-reccurence | Male | 51 | ⅡB | 0.263668167 |
| 254 | 2046 | Non-reccurence | Female | 54 | ⅢA | 0.108157514 |
| 255 | 345 | Reccurence | Male | 72 | ⅡB | 0.811154821 |
| 256 | 1654 | Reccurence | Female | 62 | ⅢB | 0.807445783 |
| 257 | 1408 | Reccurence | Male | 58 | ⅢA | 0.217745691 |
| 258 | 1896 | Non-reccurence | Male | 76 | ⅢB | -0.07572273 |
| 259 | 30 | Reccurence | Male | 73 | ⅡB | 0.631940325 |
| 260 | 1920 | Non-reccurence | Male | 75 | ⅢB | 0.400917901 |
| 261 | 514 | Reccurence | Female | 57 | ⅢB | 0.809919194 |
| 262 | 785 | Reccurence | Male | 80 | ⅡA | 0.81801152 |
| 263 | 1919 | Non-reccurence | Male | 31 | ⅡB | 0.030863596 |
| 264 | 374 | Reccurence | Male | 55 | ⅡA | 0.419662464 |
| 265 | 195 | Reccurence | Male | 72 | ⅢA | 0.705885319 |
| 266 | 638 | Reccurence | Male | 58 | ⅢB | 1.548954375 |
| 267 | 1083 | Reccurence | Female | 57 | ⅢB | 0.601978692 |
| 268 | 2011 | Non-reccurence | Male | 61 | ⅡB | 1.196565489 |
| 269 | 2355 | Reccurence | Male | 62 | ⅡA | 0.102523364 |
| 270 | 2164 | Reccurence | Male | 52 | ⅡB | 0.860030652 |
| 271 | 579 | Reccurence | Female | 50 | ⅢB | -0.54259658 |
| 272 | 794 | Reccurence | Male | 70 | ⅡA | 0.410243876 |
| 273 | 162 | Reccurence | Male | 71 | ⅡB | 0.838881733 |
| 274 | 1884 | Non-reccurence | Female | 61 | ⅡA | 0.029558093 |
| 275 | 1908 | Non-reccurence | Female | 57 | ⅡA | -0.26142241 |
| 276 | 2009 | Non-reccurence | Female | 72 | ⅢB | 0.956055523 |
| 277 | 1911 | Non-reccurence | Male | 42 | ⅠB | 0.222550953 |
| 278 | 1877 | Non-reccurence | Male | 72 | ⅠB | #N/A |
| 279 | 1913 | Non-reccurence | Female | 68 | ⅡA | 0.88955141 |
| 280 | 2017 | Non-reccurence | Male | 48 | ⅡA | -1.00206027 |
| 281 | 1853 | Non-reccurence | Male | 67 | ⅡA | 0.541040449 |
| 282 | 1887 | Non-reccurence | Male | 69 | ⅡA | 0.408353488 |
| 283 | 890 | Reccurence | Male | 70 | ⅡA | 0.186127815 |
| 284 | 1892 | Non-reccurence | Female | 65 | ⅡA | 1.109210602 |
| 285 | 1871 | Non-reccurence | Male | 57 | ⅡA | 0.770219638 |
| 286 | 1876 | Non-reccurence | Male | 74 | ⅡA | -0.26127755 |
| 287 | 180 | Reccurence | Male | 50 | ⅢB | 0.360211161 |
| 288 | 195 | Reccurence | Male | 69 | ⅡB | #N/A |
| 289 | 1885 | Non-reccurence | Male | 69 | ⅠB | 0.817747982 |
| 290 | 1908 | Non-reccurence | Female | 51 | ⅢA | 0.368909571 |
| 291 | 1906 | Non-reccurence | Male | 58 | ⅢB | -1.50519354 |
| 292 | 330 | Reccurence | Male | 68 | ⅢB | 1.264058458 |
| 293 | 297 | Reccurence | Male | 62 | ⅢB | #N/A |
| 294 | 1885 | Non-reccurence | Female | 56 | ⅡA | -0.00032194 |
| 295 | 508 | Reccurence | Male | 65 | ⅡA | 1.4478105 |
| 296 | 1856 | Non-reccurence | Male | 52 | ⅠB | 0.579325786 |
| 297 | 1418 | Reccurence | Female | 42 | ⅡB | -0.0969927 |
| 298 | 407 | Reccurence | Male | 32 | ⅡB | #N/A |
| 299 | 171 | Reccurence | Male | 45 | ⅡB | 1.403375179 |
| 300 | 179 | Reccurence | Male | 40 | ⅡA | 0.393883883 |
| 301 | 154 | Reccurence | Female | 49 | ⅢB | #N/A |
| 302 | 1854 | Non-reccurence | Female | 62 | ⅠB | -0.53603599 |
| 303 | 1847 | Non-reccurence | Male | 67 | ⅡB | 1.196595086 |
| 304 | 225 | Reccurence | Female | 55 | ⅡA | #N/A |
| 305 | 1841 | Non-reccurence | Female | 67 | ⅡA | 0.633334054 |
| 306 | 1861 | Non-reccurence | Female | 63 | ⅡA | 0.609720798 |
| 307 | 333 | Reccurence | Female | 38 | ⅡB | 0.298278598 |
| 308 | 827 | Reccurence | Male | 73 | ⅠB | 0.431636529 |
| 309 | 1825 | Reccurence | Male | 75 | ⅠA | 0.547771602 |
| 310 | 921 | Reccurence | Male | 54 | ⅡB | 1.341252984 |
| 311 | 267 | Reccurence | Male | 54 | ⅢB | 1.486135635 |
| 312 | 1845 | Non-reccurence | Male | 66 | ⅡB | -0.46313026 |
| 313 | 1956 | Non-reccurence | Female | 74 | ⅢB | 0.014762755 |
| 314 | 1337 | Reccurence | Male | 70 | ⅡA | 0.906069948 |
| 315 | 378 | Reccurence | Male | 75 | ⅡB | 0.691740248 |
| 316 | 1574 | Reccurence | Male | 52 | ⅠB | 0.773719783 |
| 317 | 565 | Reccurence | Female | 72 | ⅡA | 0.769459033 |
| 318 | 103 | Reccurence | Female | 38 | ⅢB | 0.528375772 |
| 319 | 1858 | Non-reccurence | Male | 59 | ⅡB | 0.72374922 |
| 320 | 1820 | Non-reccurence | Male | 77 | ⅡA | 1.046024466 |
| 321 | 1856 | Non-reccurence | Male | 56 | ⅠB | 0.207106596 |
| 322 | 450 | Reccurence | Female | 63 | ⅡA | 0.908772563 |
| 323 | 1828 | Non-reccurence | Female | 40 | ⅠB | 1.309711757 |
| 324 | 382 | Reccurence | Male | 65 | ⅢB | -0.07287566 |
| 325 | 1841 | Non-reccurence | Female | 61 | ⅢB | 0.045479708 |
| 326 | 1833 | Non-reccurence | Male | 56 | ⅡB | -2.14070368 |
| 327 | 1827 | Non-reccurence | Male | 58 | ⅢB | -4.68180088 |
| 328 | 1829 | Non-reccurence | Female | 58 | ⅡA | 0.306097105 |
| 329 | 1831 | Non-reccurence | Female | 69 | ⅡA | 0.08142896 |
| 330 | 469 | Reccurence | Female | 45 | ⅢB | 1.301169035 |
| 331 | 1102 | Non-reccurence | Male | 66 | ⅢB | 0.378360841 |
| 332 | 1832 | Non-reccurence | Male | 58 | ⅡA | 0.124109737 |
| 333 | 1849 | Non-reccurence | Female | 70 | ⅡA | -1.35032286 |
| 334 | 1833 | Non-reccurence | Female | 44 | ⅡA | -3.83019612 |
| 335 | 440 | Reccurence | Male | 52 | ⅢB | 1.22043581 |
| 336 | 364 | Reccurence | Male | 49 | ⅢB | 0.754983036 |
| 337 | 366 | Reccurence | Male | 53 | ⅡB | 0.985573249 |
| 338 | 1737 | Non-reccurence | Female | 73 | ⅡA | 0.374506549 |
| 339 | 1819 | Non-reccurence | Male | 55 | ⅠB | -1.27801012 |
| 340 | 1825 | Non-reccurence | Female | 56 | ⅡA | 0.176236808 |
| 341 | 1822 | Non-reccurence | Female | 47 | ⅠB | 0.444735314 |
| 342 | 662 | Reccurence | Female | 59 | ⅢB | #N/A |
| 343 | 1820 | Non-reccurence | Female | 69 | ⅡA | 0.366244375 |
| 344 | 704 | Reccurence | Male | 57 | ⅡA | 0.487005806 |
| 345 | 194 | Reccurence | Male | 42 | ⅡB | 1.601589847 |
| 346 | 1901 | Non-reccurence | Female | 53 | ⅠB | #N/A |
| 347 | 1902 | Non-reccurence | Male | 47 | ⅡB | 0.488180235 |
| 348 | 1898 | Non-reccurence | Female | 48 | ⅠB | 0.667110982 |
| 349 | 1881 | Non-reccurence | Female | 69 | ⅡA | -0.04577451 |
| 350 | 1889 | Non-reccurence | Male | 76 | ⅡA | #N/A |
| 351 | 2062 | Reccurence | Female | 58 | ⅡA | 0.710739004 |
| 352 | 205 | Reccurence | Male | 67 | ⅢB | 1.275317072 |
| 353 | 1173 | Non-reccurence | Male | 43 | ⅢB | 0.565772318 |
| 354 | 1121 | Reccurence | Male | 49 | ⅠB | 1.143468859 |
| 355 | 1894 | Non-reccurence | Female | 50 | ⅠB | 2.034444029 |
| 356 | 391 | Reccurence | Male | 54 | ⅢB | 0.177858333 |
| 357 | 1891 | Non-reccurence | Female | 78 | ⅠA | 0.438239707 |
| 358 | 1888 | Non-reccurence | Female | 54 | ⅢA | 0.752003503 |
| 359 | 1873 | Non-reccurence | Female | 77 | ⅡA | 0.985367256 |
| 360 | 160 | Reccurence | Male | 53 | ⅢA | 0.074327633 |
| 361 | 2074 | Reccurence | Male | 68 | ⅢB | -0.32875458 |
| 362 | 1879 | Non-reccurence | Male | 71 | ⅢB | -0.1974842 |
| 363 | 1863 | Non-reccurence | Male | 35 | ⅡA | #N/A |
| 364 | 1880 | Non-reccurence | Female | 79 | ⅡA | 0.990014057 |
| 365 | 152 | Reccurence | Male | 56 | ⅢB | 0.759323272 |
| 366 | 1853 | Non-reccurence | Male | 41 | ⅢB | #N/A |
| 367 | 1876 | Non-reccurence | Female | 55 | ⅢB | 0.842653444 |
| 368 | 1868 | Non-reccurence | Female | 47 | ⅢB | 0.648662515 |
| 369 | 1870 | Non-reccurence | Female | 71 | ⅠB | 0.169611076 |
| 370 | 48 | Reccurence | Female | 53 | ⅢB | 0.351150324 |
| 371 | 1863 | Non-reccurence | Male | 75 | ⅡB | 1.01300705 |
| 372 | 443 | Reccurence | Female | 54 | ⅢB | 1.067806131 |
| 373 | 1854 | Non-reccurence | Male | 59 | ⅡA | 0.14613266 |
| 374 | 1846 | Non-reccurence | Male | 67 | ⅡA | #N/A |
| 375 | 1849 | Non-reccurence | Male | 55 | ⅡB | 1.066616926 |
| 376 | 1847 | Non-reccurence | Female | 66 | ⅢB | -0.45329304 |
| 377 | 1743 | Non-reccurence | Female | 78 | ⅢB | 0.378510157 |
| 378 | 1847 | Non-reccurence | Female | 43 | ⅠB | -0.5085211 |
| 379 | 161 | Reccurence | Male | 77 | ⅡB | 0.891593681 |
| 380 | 876 | Reccurence | Female | 51 | ⅢB | 0.196976127 |
| 381 | 1834 | Non-reccurence | Female | 76 | ⅠA | 0.049044931 |
| 382 | 867 | Reccurence | Female | 60 | ⅡB | #N/A |
| 383 | 1847 | Non-reccurence | Female | 62 | ⅠB | 0.31258702 |
| 384 | 1848 | Non-reccurence | Male | 62 | ⅡA | 0.133338176 |
| 385 | 1845 | Non-reccurence | Female | 54 | ⅢB | 0.463380386 |
| 386 | 77 | Reccurence | Male | 57 | ⅢB | 1.108060158 |
| 387 | 592 | Reccurence | Male | 65 | ⅡB | 1.648229351 |
| 388 | 1835 | Non-reccurence | Male | 67 | ⅠB | 1.308638383 |
| 389 | 115 | Reccurence | Male | 57 | ⅢB | 0.381026678 |
| 390 | 427 | Reccurence | Female | 56 | ⅠB | 0.793679123 |
| 391 | 191 | Reccurence | Male | 56 | ⅠA | 1.209304298 |
| 392 | 1841 | Non-reccurence | Male | 59 | ⅢB | -0.76833584 |
| 393 | 277 | Reccurence | Male | 64 | ⅢB | #N/A |
| 394 | 1834 | Non-reccurence | Male | 30 | ⅡA | 0.193214535 |
| 395 | 1840 | Non-reccurence | Male | 57 | ⅢB | 0.010436832 |
| 396 | 1839 | Non-reccurence | Male | 77 | ⅠA | -0.55624749 |
| 397 | 916 | Non-reccurence | Male | 55 | ⅡA | -0.47168789 |
| 398 | 195 | Reccurence | Male | 55 | ⅢA | -2.82227478 |
| 399 | 1834 | Non-reccurence | Male | 67 | ⅡB | #N/A |
| 400 | 1836 | Non-reccurence | Female | 51 | ⅡA | -0.09063579 |
| 401 | 1352 | Non-reccurence | Female | 50 | ⅠB | #N/A |
| 402 | 1742 | Reccurence | Male | 65 | ⅢB | 0.638689327 |
| 403 | 737 | Reccurence | Female | 63 | ⅡA | -0.3042212 |
| 404 | 1073 | Reccurence | Male | 79 | ⅡA | 1.31078181 |
| 405 | 1803 | Non-reccurence | Male | 65 | ⅠB | -1.48050403 |
| 406 | 1203 | Non-reccurence | Female | 65 | ⅡA | -0.73563031 |
| 407 | 705 | Non-reccurence | Male | 47 | ⅡA | -1.55562203 |
| 408 | 235 | Reccurence | Male | 73 | ⅠB | #N/A |
| 409 | 313 | Reccurence | Male | 66 | ⅡA | 1.944483431 |
| 410 | 621 | Reccurence | Female | 57 | ⅢB | #N/A |
| 411 | 889 | Reccurence | Male | 54 | ⅠB | -0.1653849 |
| 412 | 1756 | Non-reccurence | Male | 73 | ⅡA | -6.89863177 |
| 413 | 1075 | Non-reccurence | Male | 59 | ⅢB | 2.003807163 |
| 414 | 319 | Reccurence | Female | 50 | ⅢA | 1.187940254 |
| 415 | 1827 | Non-reccurence | Female | 52 | ⅢB | 0.176846482 |
| 416 | 1787 | Non-reccurence | Male | 55 | ⅡB | #N/A |
| 417 | 693 | Non-reccurence | Female | 35 | ⅡA | 0.036497969 |
| 418 | 771 | Non-reccurence | Female | 54 | ⅡA | 0.367422892 |
| 419 | 1999 | Non-reccurence | Male | 78 | ⅡA | -0.59166219 |
| 420 | 521 | Reccurence | Male | 60 | ⅢB | 0.138471993 |
| 421 | 691 | Non-reccurence | Female | 70 | ⅡA | 0.380319905 |
| 422 | 1780 | Non-reccurence | Female | 69 | ⅡA | 0.34155462 |
| 423 | 18 | Non-reccurence | Female | 61 | ⅡB | 0.896728646 |
| 424 | 1792 | Non-reccurence | Male | 25 | ⅡA | 0.360484116 |
| 425 | 1286 | Reccurence | Female | 85 | ⅡA | #N/A |
| 426 | 1775 | Non-reccurence | Female | 64 | ⅡA | 0.658277465 |
| 427 | 418 | Reccurence | Female | 49 | ⅢB | #N/A |
| 428 | 1764 | Non-reccurence | Female | 71 | ⅡB | 0.879077796 |
| 429 | 1767 | Non-reccurence | Male | 69 | ⅡB | #N/A |
| 430 | 1063 | Non-reccurence | Female | 75 | ⅠB | 0.234782976 |
| 431 | 253 | Non-reccurence | Male | 68 | ⅡA | 0.950323516 |
| 432 | 1755 | Non-reccurence | Male | 45 | ⅡA | #N/A |
| 433 | 1756 | Non-reccurence | Male | 71 | ⅡA | #N/A |
| 434 | 709 | Reccurence | Male | 73 | ⅢB | #N/A |
| 435 | 1761 | Non-reccurence | Male | 48 | ⅡB | -0.22587169 |
| 436 | 1751 | Non-reccurence | Male | 67 | ⅡB | #N/A |
| 437 | 1752 | Non-reccurence | Male | 68 | ⅡB | 0.731086461 |
| 438 | 1756 | Non-reccurence | Male | 63 | ⅡA | #N/A |
| 439 | 441 | Reccurence | Male | 67 | ⅠB | #N/A |
| 440 | #REF! | Reccurence | Male | 56 | ⅢA | #N/A |
| 441 | 667 | Reccurence | Male | 59 | ⅢB | 2.653245934 |
| 442 | 1740 | Non-reccurence | Male | 57 | ⅢA | -0.15146697 |
| 443 | 395 | Reccurence | Male | 66 | ⅡB | 0.305268126 |
| 444 | 336 | Reccurence | Male | 63 | ⅢA | #N/A |
| 445 | 337 | Non-reccurence | Male | 69 | ⅠB | -1.06243099 |
| 446 | 955 | Non-reccurence | Male | 73 | ⅡA | -0.24042693 |
| 447 | 1890 | Non-reccurence | Female | 65 | ⅠA | #N/A |
| 448 | 1366 | Non-reccurence | Male | 44 | ⅠB | 0.767714188 |
| 449 | 1725 | Non-reccurence | Male | 67 | ⅠB | 0.082635043 |
| 450 | 774 | Reccurence | Female | 56 | ⅢA | 1.607543735 |
| 451 | 1744 | Non-reccurence | Female | 72 | ⅠB | 1.075144464 |
| 452 | 418 | Reccurence | Male | 58 | ⅢB | #N/A |
| 453 | 1737 | Non-reccurence | Male | 60 | ⅡA | #N/A |
| 454 | 1737 | Non-reccurence | Female | 66 | ⅢA | #N/A |
| 455 | 1762 | Non-reccurence | Male | 56 | ⅠB | -4.87094613 |
| 456 | 679 | Reccurence | Female | 80 | ⅢB | 1.036965695 |
| 457 | 1725 | Non-reccurence | Male | 46 | ⅠB | #N/A |
| 458 | 1720 | Non-reccurence | Male | 69 | ⅡB | #N/A |
| 459 | 1014 | Non-reccurence | Female | 61 | ⅡB | 0.395780227 |
| 460 | 1737 | Non-reccurence | Male | 63 | ⅡB | -0.45456345 |
| 461 | 1721 | Non-reccurence | Female | 51 | ⅡB | -0.12340872 |
| 462 | 1713 | Non-reccurence | Female | 64 | ⅢA | -0.38802318 |
| 463 | 1716 | Non-reccurence | Male | 23 | ⅢB | -0.14698172 |
| 464 | 1723 | Non-reccurence | Female | 68 | ⅡA | #N/A |
| 465 | 1713 | Non-reccurence | Female | 66 | ⅡA | #N/A |
| 466 | 440 | Non-reccurence | Female | 55 | ⅡA | 0.310106083 |
| 467 | 952 | Reccurence | Male | 64 | ⅢA | 1.458619712 |
| 468 | 1002 | Non-reccurence | Male | 55 | ⅢA | 0.87238627 |
| 469 | 26 | Reccurence | Female | 61 | ⅢA | #N/A |
| 470 | 138 | Reccurence | Female | 67 | ⅢA | #N/A |
| 471 | 679 | Reccurence | Female | 66 | ⅡA | #N/A |
| 472 | 1706 | Non-reccurence | Male | 43 | ⅡA | #N/A |
| 473 | 1749 | Reccurence | Male | 60 | ⅠB | 0.030435742 |
| 474 | 1807 | Reccurence | Male | 62 | ⅡB | #N/A |
| 475 | 1701 | Non-reccurence | Female | 57 | ⅠB | -0.48346837 |
| 476 | 10 | Reccurence | Male | 53 | ⅢB | 1.925147211 |
| 477 | 1701 | Non-reccurence | Male | 53 | ⅠB | 0.721444479 |
| 478 | 1705 | Non-reccurence | Female | 61 | ⅢA | #N/A |
| 479 | 1677 | Non-reccurence | Male | 67 | ⅡA | 0.199566783 |
| 480 | 572 | Reccurence | Male | 47 | ⅢB | #N/A |
| 481 | 1085 | Reccurence | Male | 74 | ⅡB | 1.792011387 |
| 482 | 1699 | Non-reccurence | Female | 66 | ⅡB | #N/A |
| 483 | 1778 | Non-reccurence | Female | 66 | ⅡA | -0.19086632 |
| 484 | 1575 | Reccurence | Male | 75 | ⅠB | #N/A |
| 485 | 1693 | Non-reccurence | Female | 63 | ⅡA | 1.4046207 |
| 486 | 1679 | Non-reccurence | Male | 72 | ⅢB | #N/A |
| 487 | 177 | Reccurence | Female | 51 | ⅢB | 1.143013375 |
| 488 | 164 | Reccurence | Male | 79 | ⅡB | #N/A |
| 489 | 1677 | Non-reccurence | Male | 52 | ⅠB | #N/A |
| 490 | 1663 | Non-reccurence | Female | 54 | ⅠB | 0.521655937 |
| 491 | 1712 | Reccurence | Male | 68 | ⅠB | #N/A |
| 492 | 953 | Reccurence | Male | 65 | ⅢB | #N/A |
| 493 | 1656 | Non-reccurence | Male | 60 | ⅠB | #N/A |
| 494 | 1469 | Non-reccurence | Female | 66 | ⅢB | #N/A |
| 495 | 1316 | Reccurence | Male | 53 | ⅢB | #N/A |
| 496 | 397 | Reccurence | Male | 52 | ⅡA | #N/A |
| 497 | 1651 | Non-reccurence | Male | 57 | ⅡB | 0.723753281 |
| 498 | 123 | Non-reccurence | Male | 77 | ⅢB | 0.65212031 |
| 499 | 1163 | Non-reccurence | Male | 77 | ⅠB | -2.99194583 |
| 500 | 1172 | Non-reccurence | Female | 48 | ⅢB | #N/A |
| 501 | 1070 | Non-reccurence | Male | 44 | ⅠB | 0.388038402 |
| 502 | 497 | Reccurence | Female | 49 | ⅢB | 0.511518323 |
| 503 | 928 | Non-reccurence | Male | 75 | ⅠB | -0.65688314 |
| 504 | 1652 | Non-reccurence | Male | 42 | ⅢB | #N/A |
| 505 | 1647 | Non-reccurence | Male | 53 | ⅠB | -2.42037012 |
| 506 | 1710 | Non-reccurence | Male | 45 | ⅠA | 0.781015664 |
| 507 | 208 | Reccurence | Male | 63 | ⅠB | #N/A |
| 508 | 1596 | Non-reccurence | Male | 72 | ⅠB | 1.504978163 |
| 509 | 921 | Non-reccurence | Male | 80 | ⅡA | 0.387987788 |
| 510 | 1129 | Non-reccurence | Female | 51 | ⅠB | 2.282461658 |
| 511 | 1813 | Non-reccurence | Male | 44 | ⅡA | 0.47366294 |
| 512 | 1504 | Non-reccurence | Male | 56 | ⅠB | -0.56983667 |
| 513 | 378 | Reccurence | Male | 53 | ⅢB | 0.693866977 |
| 514 | 904 | Non-reccurence | Male | 72 | ⅡA | #N/A |
| 515 | 1497 | Non-reccurence | Female | 65 | ⅡA | 0.31678373 |
| 516 | 514 | Reccurence | Female | 63 | ⅡB | -1.01036557 |
| 517 | 1849 | Non-reccurence | Female | 64 | ⅠB | #N/A |
| 518 | 1632 | Non-reccurence | Male | 74 | ⅡA | #N/A |
| 519 | 515 | Non-reccurence | Female | 49 | ⅢB | #N/A |
| 520 | 1022 | Non-reccurence | Male | 69 | ⅡB | 1.392148753 |
| 521 | 0 | Reccurence | Male | 58 | ⅢA | -0.09540187 |
| 522 | 158 | Reccurence | Male | 69 | ⅡA | #N/A |
| 523 | 896 | Non-reccurence | Female | 68 | ⅠA | 0.473432785 |
| 524 | 1613 | Non-reccurence | Male | 79 | ⅠB | 0.385721258 |
| 525 | 1439 | Non-reccurence | Female | 55 | ⅠB | 0.066069755 |
| 526 | 889 | Non-reccurence | Male | 57 | ⅠB | 0.225500324 |
| 527 | 504 | Non-reccurence | Female | 34 | ⅠB | #N/A |
| 528 | 1702 | Reccurence | Female | 49 | ⅡA | #N/A |
| 529 | 1225 | Reccurence | Male | 48 | ⅠA | 0.236285353 |
| 530 | 884 | Non-reccurence | Female | 64 | ⅠB | -0.25132813 |
| 531 | 1585 | Non-reccurence | Female | 53 | ⅡB | 0.507523474 |
| 532 | 1587 | Non-reccurence | Female | 79 | ⅡA | #N/A |
| 533 | 321 | Reccurence | Female | 58 | ⅡA | #N/A |
| 534 | 889 | Non-reccurence | Male | 60 | ⅡA | #N/A |
| 535 | 1596 | Non-reccurence | Female | 60 | ⅡA | 0.238592211 |
| 536 | 1474 | Non-reccurence | Male | 58 | ⅡA | #N/A |
| 537 | 1592 | Non-reccurence | Male | 63 | ⅠB | -1.28522091 |
| 538 | 1026 | Non-reccurence | Female | 40 | ⅢB | 0.705481561 |
| 539 | 297 | Reccurence | Male | 60 | ⅠB | 0.486188069 |
| 540 | 1584 | Non-reccurence | Female | 70 | ⅢB | #N/A |
| 541 | 422 | Reccurence | Male | 74 | ⅡB | #N/A |
| 542 | 1585 | Non-reccurence | Female | 59 | ⅠB | 0.427059924 |
| 543 | 1569 | Non-reccurence | Female | 62 | ⅠB | 0.374545342 |
| 544 | 1582 | Non-reccurence | Male | 74 | ⅢA | #N/A |
| 545 | 875 | Non-reccurence | Male | 79 | ⅠB | #N/A |
| 546 | 1579 | Non-reccurence | Female | 73 | ⅡA | -0.35910683 |
| 547 | 651 | Reccurence | Female | 62 | ⅢB | 0.596362591 |
| 548 | 400 | Reccurence | Male | 64 | ⅡA | -0.23254186 |
| 549 | 1569 | Non-reccurence | Female | 48 | ⅢB | 0.803702041 |
| 550 | 763 | Reccurence | Male | 43 | ⅢB | 0.815582122 |
| 551 | 1570 | Non-reccurence | Female | 69 | ⅡA | 0.038249192 |
| 552 | 671 | Reccurence | Male | 60 | ⅢA | #N/A |
| 553 | 1132 | Reccurence | Female | 54 | ⅢB | 0.913713904 |

*IRRS: Immune-related risk score, #N/A: Non-available.
