# Supplementary material for: Multiplex immunofluorescence and single‐cell transcriptomic profiling reveal the spatial cell interaction networks in the non‐small cell lung cancer microenvironment
Source: Clin Transl Med. 2023 Jan 1;13(1):e1155. doi: 10.1002/ctm2.1155 (PMC9806015; doi:10.1002/ctm2.1155)
Supplement: Supplementary file 24 — Supporting information. Supplementary table 6. Univariate Cox regression model demonstrated the prognostic effects of the immune‐related risk score model. [file CTM2-13-e1155-s014.docx]

| **Variables** | **HR (95%CI)** | **P-value** |
| --- | --- | --- |
| **Age** | 1.01 (1.00, 1.02) | 0.165 |
| **Sex** |  |  |
| Male | Ref. |  |
| Female | 0.65 (0.50, 0.85) | 0.002 |
| **Tstage** |  |  |
| T1 | Ref. |  |
| T2 | 1.11 (0.82, 1.52) | 0.507 |
| T3 | 1.60 (1.12, 2.30) | 0.011 |
| T4 | 2.59 (1.69, 3.97) | <0.001 |
| **Nstage** |  |  |
| N0 | Ref. |  |
| N1 | 2.69 (1.91, 3.79) | <0.001 |
| N2 | 3.29 (2.47, 4.38) | <0.001 |
| **Visceral pleural invasion** |  |  |
| PL0 | Ref. |  |
| PL1 | 1.39 (1.05, 1.84) | 0.021 |
| PL2 | 1.43 (0.84, 2.42) | 0.183 |
| **Vascular tumor emboli** |  |  |
| No | Ref. |  |
| Yes | 1.95 (1.49, 2.56) | <0.001 |
| **Resected lymph nodes** | 0.99 (0.98,1.01) | 0.141 |
| **Immune-related risk score** |  |  |
| Low | Ref. |  |
| High | 2.98 (2.21, 4.01) | <0.001 |

**Supplementary table 6.** Univariate Cox regression model demonstrated the prognostic effects of the immune-related risk score model.
