# Supplementary material for: Multiplex immunofluorescence and single‐cell transcriptomic profiling reveal the spatial cell interaction networks in the non‐small cell lung cancer microenvironment
Source: Clin Transl Med. 2023 Jan 1;13(1):e1155. doi: 10.1002/ctm2.1155 (PMC9806015; doi:10.1002/ctm2.1155)
Supplement: Supplementary file 25 — Supplementary table 7. Marker genes for annotation of major cell types in internal single‐cell RNA sequencing dataset of lung adenocarcinoma. [file CTM2-13-e1155-s009.docx]

**Supplementary table 7.** Marker genes for annotation of cell types in internal single-cell RNA sequencing dataset of lung adenocarcinoma.

| **Cell type** | **Marker gene 1** | **Marker gene 2** | **Marker gene 3** |
| --- | --- | --- | --- |
| **T Cells** | CD3D | GZMK | CXCL13 |
| **Natural killer cells** | NKG7 | CCL5 | GNLY |
| **Macrophages** | CD163 | LYZ | FCGR3A |
| **Neutrophils** | G0S2 | S100A9 | RSAD2 |
| **Epithelial cells** | KRT8 | KRT16 | CLDN4 |
| **Fibroblasts** | COL1A1 | COL3A1 | LUM |
| **Dendritic cells** | IFI30 | AIF1 | LST1 |
| **B cells** | CD79A | MS4A1 | BANK1 |
| **Mast cells** | MS4A2 | TMEM233 | SLC18A2 |
| **Stem cells** | TBX2 | ACAN | MCAM |
| **Endothelial cells** | EMCN | TEK | HAPLN1 |
| **Cancer cells** | MMP7 | MMP13 | HOXB2 |
