# Supplementary material for: Multiplex immunofluorescence and single‐cell transcriptomic profiling reveal the spatial cell interaction networks in the non‐small cell lung cancer microenvironment
Source: Clin Transl Med. 2023 Jan 1;13(1):e1155. doi: 10.1002/ctm2.1155 (PMC9806015; doi:10.1002/ctm2.1155)
Supplement: Supplementary file 26 — Supporting information. Supplementary table 8. Marker genes for annotation of major cell types in external single‐cell RNA sequencing dataset of lung adenocarcinoma. [file CTM2-13-e1155-s022.docx]

**Supplementary table 8.** Marker genes for annotation of major cell types in external single-cell RNA sequencing dataset of lung adenocarcinoma.

| **Cell type** | **Marker gene 1** | **Marker gene 2** | **Marker gene 3** |
| --- | --- | --- | --- |
| **T Cells** | CD3D | CD3E | CD2 |
| **Natural killer cells** | NKG7 | GZMA | KLRD1 |
| **Macrophages** | CD68 | CD163 | LYZ |
| **Neutrophils** | G0S2 | LST1 | MNDA |
| **Epithelial cells** | KRT7 | KRT18 | KRT8 |
| **Fibroblasts** | COL3A1 | COL1A1 | LUM |
| **Dendritic cells** | CD1C | CD83 | HLA-DQB1 |
| **B cells** | CD79A | IGHG1 | MZB1 |
| **Mast cells** | TPSB2 | TPSAB1 | CPA3 |
| **Stem cells** | EPCAM | TACSTD2 | IFI27 |
| **Endothelial cells** | EMCN | IGFBP4 | TM4SF1 |
