# Supplementary material for: Multiplex immunofluorescence and single‐cell transcriptomic profiling reveal the spatial cell interaction networks in the non‐small cell lung cancer microenvironment
Source: Clin Transl Med. 2023 Jan 1;13(1):e1155. doi: 10.1002/ctm2.1155 (PMC9806015; doi:10.1002/ctm2.1155)
Supplement: Supplementary file 27 — Supporting information. Supplementary table 9. Marker genes for annotation of major cell types in external single‐cell RNA sequencing dataset of squamous cell lung cancer. [file CTM2-13-e1155-s024.docx]

**Supplementary table 9.** Marker genes for annotation of major cell types in external single-cell RNA sequencing dataset of squamous cell lung cancer.

| **Cell type** | **Marker gene 1** | **Marker gene 2** | **Marker gene 3** |
| --- | --- | --- | --- |
| **T Cells** | CD3D | CD2 | CXCL13 |
| **Natural killer cells** | NKG7 | GNLY | CCL5 |
| **Macrophages** | CD163 | FCGR3A | LYZ |
| **Neutrophils** | S100A9 | S100A11 | S100A8 |
| **Epithelial cells** | KRT8 | CLDN4 | SFTPB |
| **Fibroblasts** | COL3A1 | COL1A1 | LUM |
| **Dendritic cells** | CST3 | - | - |
| **B cells** | CD79A | MS4A1 | CD79B |
| **Mast cells** | SLC18A2 | TPSAB1 | TPSB2 |
